# Systematic Review of Sex and Gender Effects in Traumatic Brain Injury: Equity in Clinical and Functional Outcomes

**DOI:** 10.3389/fneur.2021.678971

**Published:** 2021-09-10

**Authors:** Tatyana Mollayeva, Shirin Mollayeva, Nicole Pacheco, Angela Colantonio

**Affiliations:** ^1^KITE Toronto Rehabilitation Institute University Health Network, Toronto, ON, Canada; ^2^Rehabilitation Sciences Institute, Faculty of Medicine, University of Toronto, Toronto, ON, Canada; ^3^Acquired Brain Injury Research Lab, Temerty Faculty of Medicine University of Toronto, Toronto, ON, Canada; ^4^School of Occupational Therapy, Western University, London, ON, Canada; ^5^Department of Epidemiology, Dalla Lana School of Public Health University of Toronto, Toronto, ON, Canada

**Keywords:** best-evidence synthesis, gender equality, outcomes, social equity, traumatic brain injury, sex differences

## Abstract

**Background:** Although traumatic brain injury (TBI) is a leading cause of death and disability in male and female patients worldwide, little is known about the effect of sex and gender on TBI outcomes.

**Objectives:** This systematic review summarizes the evidence on the effect of sex and gender on core TBI outcomes.

**Methods:** All English-language studies from six literature databases that addressed core outcomes in adults with TBI and included sex or gender, TBI severity, and age in their analyses were considered eligible. Two reviewers extracted data, and two reviewers assessed study quality using tools recommended by the National Institutes of Health. The results were sorted according to time post-injury, injury severity, gender equity ranking of the study's country of origin, and outcomes studied. The results from the included studies were grouped based on the approach taken in reporting their respective findings.

**Results and Limitations:** Of 172 articles assessed, 58 studies were selected, comprising 1, 265, 955 participants with TBI (67% male across all studies) of all injury severities. All studies were conducted in countries with a very high or high human development index, while the Gender Inequality Index (GII) varied. While the heterogeneity across studies limited any meaningful conclusions with respect to the role of sex and gender, we did observe that as gender equality ranking improved, differences between male and female participants in outcomes would diminish. Inclusion of social equity parameters in the studies was limited.

**Conclusions and Implications:** The non-uniform findings observed bring forth the need to develop and use a comprehensive and consistent methodology in the study of sex and gender post-TBI, incorporating social equity parameters to uncover the potential social underpinnings of gender effects on health and functional outcomes.

**Systematic Review Registration:** CRD42018098697.

## Highlights

- The effects of sex and gender on outcomes in traumatic brain injury, while compelling, require refinement.
- This paper synthesized evidence pertinent to sex and gender effects on core TBI outcomes.
- Most results show no sex and gender effects on health- and disability-related outcomes.
- Research that observed sex and gender effects showed great variability in methods and findings, which preclude formation of meaningful conclusions.
- Attention to social equity in sex and gender inclusive research and practice is timely.

## Introduction

Traumatic brain injury (TBI) is a significant contributor to worldwide mortality and morbidity rates. Our understanding of risk and exposure and the disease process of TBI is still evolving, with the goal of informing prevention and rehabilitation efforts (1). Recently, emerging sex and gender trends in TBI have highlighted previously underappreciated factors relevant to the discussion of prevention and rehabilitation of TBI (2, 3). Sex refers to biological attributes of humans, including physical features, chromosomes, gene expression, hormones, and anatomy, whereas gender represents the socially constructed roles, behaviors, expressions, and identities of girls, women, boys, men, and gender-diverse people (4).

Differences have been observed in how men and women experience TBI and other health outcomes, driven by biological differences between the sexes, social differences, or both (2, 5). Some studies have captured greater adversity in men post-TBI, with men more likely to sustain an injury at work, exhibit impairment in executive function post-injury, struggle with community and home integration, be more prone to aggression, and have a higher likelihood of involvement in the justice system (2). Others have pointed that women are more likely to experience assault-related TBI at work and more likely to die from head injury in general and from head injury stemming from assault in particular (2). Women have also been found to report greater symptom severity post-TBI than men and to participate less in the workforce, reducing their hours or stopping work altogether (2). No differences have been found in other outcomes, for example, in the frequency and severity of pain and insomnia, and in the distribution of the sleep stages, or in productivity and social integration post-injury (2). In humans especially, it is difficult, if not impossible, to isolate the effects of sex from those of gender, because biology exists in the background of one's constantly changing environment; and it likewise shapes social context through expectations placed on men, women, boys, and girls on the basis of their sex (2, 4, 6–8). With the understanding that the constructs of sex and gender are interconnected, and their independent contributions to TBI outcomes are hardly feasible to disentangle given their respective vastness, going forward, we will use the term “sex/gender”.

The growing evidence showing that men and women experience TBI differently and may thus necessitate different approaches in preventive efforts and response post-injury has compelled funding agencies, the federal government, and researchers to implement requirements for the explicit consideration of sex/gender in research and clinical contexts (9–21).

To date, several systematic reviews have provided insight into sex/gender differences in TBI; most focused on concussion (mild TBI) (22–26). The applicability of the findings to civilian or military-related injuries in general, across the range of TBI severities, remains uncertain. There have also been missed opportunities to discuss sex/gender in relation to all core TBI outcomes (27). Previous reviews have also shown limited attention to social determinants in the studied outcomes, despite the fact that gender norms around the world can impose risk or protective effects in the context of TBI (28, 29). Accordingly, some studies have reported gender inequality in healthcare, stemming from differences in injury exposures and access to healthcare resources (30–35). Incorporating a study's country of origin in reviews may thus help researchers and healthcare policymakers understand larger influences to inform sex/gender-sensitive interventions.

It has been argued that sex/gender effects lose relevance when other personal factors such as age and injury severity are considered (36). The divergent views on the relevance of sex/gender in general and in TBI in particular call for a systematic approach to literature synthesis with a focus on social equity parameters that have previously received limited or no attention (2, 23–26). This review consolidates the data on sex and gender in relation to TBI outcomes through the Gender Inequality Index (GII) lens (35). The index was created by the United Nations as an indication of each nation's level of gender equality based on attainment of higher education, labor force participation, maternal mortality rate, adolescent fertility rate, and parliamentary representation (35). Along with gender equality, recent systematic review guidelines stress the importance of considering social equity parameters in health-related outcomes (37–39). Social variables (i.e., place of residence, occupation, race/ethnicity, and education) may predispose male and female persons to differing outcomes, due to initial disparities in health equity indicators, such as material deprivation, lack of comprehensive health coverage, transportation, and healthcare workers' potential biases (39–41).

The aims of this work were as follows: 1) document the core set of TBI outcomes and the reported sex/gender effects; 2) synthesize the data on the effects of sex/gender on TBI outcomes; 3) discuss the results in relation to gender equality and social equity parameters; and 4) identify pitfalls in existing studies, suggest directions for future research, and provide information to both researchers and clinicians on how they should approach the subject within their fields based on the trends uncovered.

## Methods

### Data

This review followed the Preferred Reporting Items for Systematic Reviews and Meta-Analyses-Equity (PRISMA-E) guidelines, (36) and its protocol was registered with the International Prospective Register of Systematic Reviews (PROSPERO) on June 19, 2018. (42) We collected sex-related information in compliance with the European Association of Science Editors' SAGER guidelines, (43) and we developed a comprehensive search strategy for TBI studies reporting on sex/gender in collaboration with a medical information specialist. Specifically, the search strategy included used text words and subject headings (MeSH, Emtree) related to 1) TBI and 2) sex, or 3) gender. We identified all English-language peer-reviewed studies, irrespective of the research setting, published from each database inception to February 2018 through the Cochrane Central Register of Controlled Trials, Cumulative Index to Nursing & Allied Health Literature, Embase, Ovid MEDLINE (epub ahead of print, in-process, and other non-indexed citations, as well as Ovid MEDLINE Daily), PsycINFO, and Web of Science databases. We also considered works in the identified articles' bibliographies. Finally, we performed a repeat search of all databases in September 2019 to identify any new publications. The complete search strategy is available in Supplementary Table 1. The PRISMA-E reporting checklist is available in Supplementary Table 2.

### PICOS criteria

- P (population)—adult male and female persons with TBI (mean age of the cohort greater than 18 years of age) living in any geographic region worldwide.
- I (intervention)—not applicable.
- C (comparison groups)—male and female persons with TBI.
- O (outcomes)—all TBI outcomes included in the selected studies.
- S (study types)—quantitative: cohort, cross-sectional, case series; qualitative studies. Studies had to include at least TBI severity, age, and sex in their analyses.

### Inclusion Criteria

We considered original peer-reviewed English-language studies of all designs that included 1) adults with TBI diagnoses based on clinician or specialist assessments of loss of consciousness, post-traumatic amnesia, or other clinical indicators as well as diagnostic codes indicating TBI (e.g., International Statistical Classification of Diseases and Related Health Problems); 2) adequate description of at least three key participant characteristics (age, sex, and TBI severity); and 3) sex/gender-stratified results that considered age and TBI severity. We also examined studies that considered age and injury severity, and utilized sex/gender as a covariate in statistical analyses of any core TBI outcomes. Specifically, they must have 4) quantified the magnitude of the association or 5) qualitatively discussed the implications of sex/gender.

### Exclusion Criteria

We excluded studies that 1) did not specify how a TBI diagnosis was made, 2) investigated TBI as an outcome, 3) compared TBI with other clinical populations without providing results on the effect of sex/gender in TBI separately, or 4) focused on sports-related concussions. The latter exclusion aimed to avoid redundancy, due to recently published systematic reviews on the topic (24–26). Furthermore, we excluded case reports, pediatric studies, dissertations, articles with no primary data; single-sex studies; and research that omitted TBI severity and age.

### Study Selection

**Figure 1:**
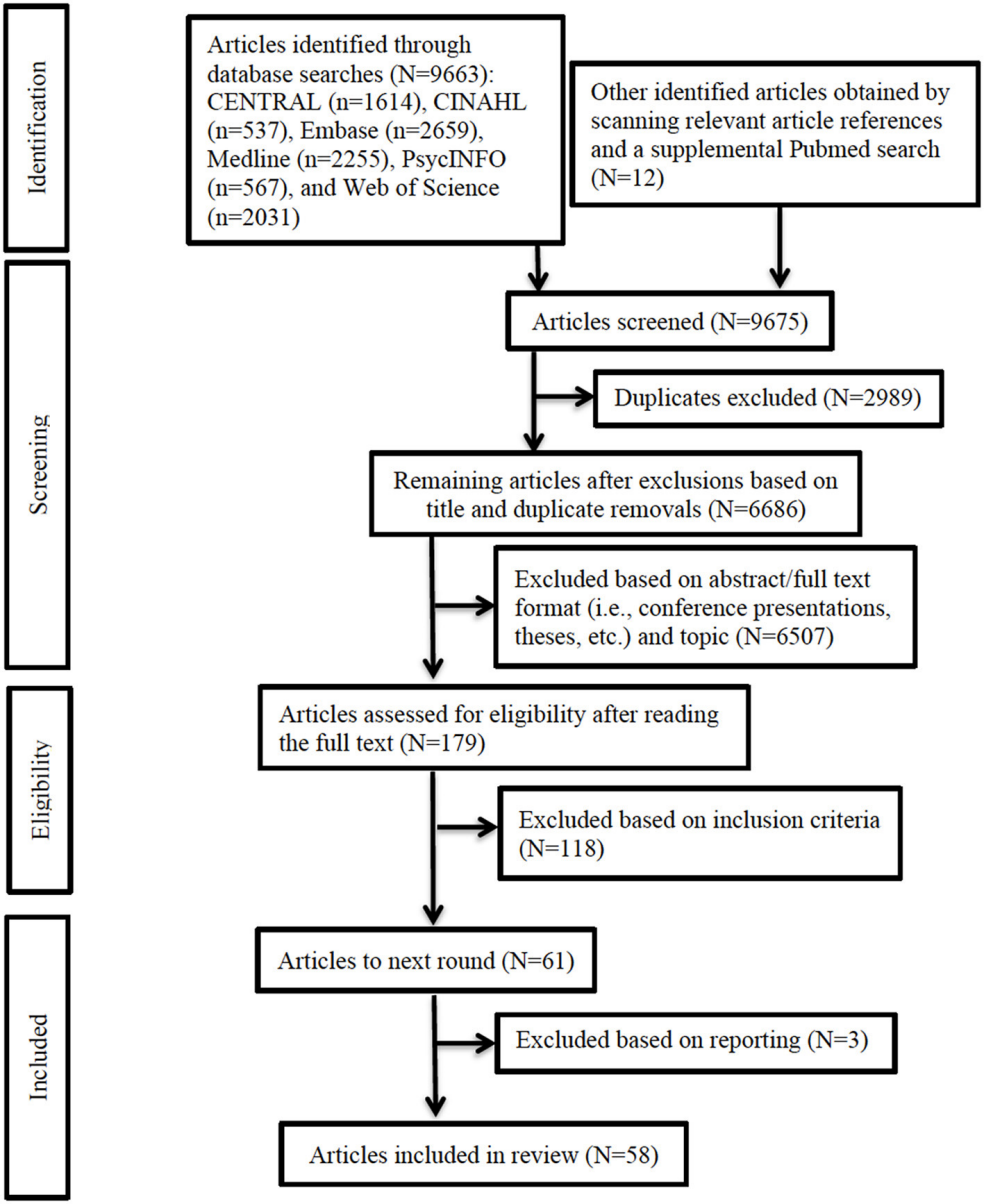
PRISMA flow diagram. Flowchart depicting study selection process and outcome. PRISMA, Preferred Reporting Items for Systematic Reviews and Meta-Analyses.

Two independent reviewers (NP and SM) assessed the study titles and abstracts for compliance with the inclusion criteria. A third reviewer (TM) randomly selected 5% of the data and assessed the quality of the two reviewers' abstraction. Differences in opinion were resolved with discussion. Then, each reviewer individually assessed the selected full texts to determine their compliance with inclusion criteria. Figure 1 depicts the entire methodological and inclusion/exclusion process, and Supplementary Table 2 lists the reasons for exclusion of 117 articles.

### Quality Assessment

Two reviewers (TM and NP) independently assessed study quality using the National Institutes of Health study quality assessment tools (44). Quantitative studies were appraised in two steps. The first step assessed items related to potential sources of bias according to the most critical criteria for external and internal design validity within cohort, cross-sectional, case–control, or intervention studies. The second step judged the presence of potential biases as “yes,” “not reported,” or “cannot determine.” The two reviewers initially met for a calibration review, in which they independently reviewed one study of each type and discussed each item on the list to clarify its meaning and interpretation. Following this, the methodological quality of each study was rated across a set of items, independently by the same two reviewers. In cases of disagreement between the two reviewers, a team discussion took place (TM, SM, and NP), following which consensus was reached in each case. We used the Scottish Intercollegiate Guidelines Network methodology to summarize the evidence, as follows: ++ (high) when all quality criteria were fulfilled, permitting one “cannot determine”; + (moderate) when most criteria were fulfilled; and – (low) when few criteria were fulfilled. Qualitative studies were appraised using the Critical Appraisal Skills Programme qualitative checklist (44, 45).

### Data Extraction and Synthesis

Two reviewers independently extracted 1) study information (authors, publication year, country, GII rank, objectives, design, sample size, outcomes studied, measures used, and how sex/gender effects was investigated), 2) participant information (age, sex, injury severity, and phase post-injury), 3) statistical approaches and controlled factors, 4) sex/gender-related findings, and 5) axes of inequality (PROGRESS indicators—place of residence; race/ethnicity/culture/language; occupation; sex/gender; religion; education; socioeconomic status; and social capital). We used a best-evidence synthesis approach (i.e., tabulation and qualitative description) to organize the findings from studies judged as having sufficient quality (46). Specifically, of the 9, 664 articles identified in our literature searches, we selected 176 for full-text review and included 58 in the final review.

All 58 studies were judged to be of moderate quality (“+”), based on the number of weaknesses identified. This does not capture the variability in quality of each study within the different domains of bias. We recognize that such a judgment of bias is somewhat arbitrary and anchors the assessment to the tool we used. The details of our evaluation of bias for each included study are presented in Supplementary Tables 3A–C. The level of agreement between the two reviewers on the quality of observational studies ranged between 100% (for items 1–4, 7–10, 12, and 13) and 85% for item 11 (Supplementary Table 3A). The most common discrepancy was in the designation of “Cannot determine” or “Unsure” vs. a definite rating. For example, there were “Yes,” “No,” “Cannot determine,” “Not applicable,” and “Not reported” options available to rate the extent of adherence to the criteria for six potential sources of biases. If an item was rated as “No,” it was regarded as a weakness. If little information was provided for item 5 (Were sample size justification, power description, or variance and effect estimates provided?) in Supplementary Table 3A, that means that there were no clear indicators to distinguish “Cannot determine” (described all components but not in detail) and “Not reported” (did not describe). Thus, we rated all studies that provide at least some information as “Yes” for a conservative evaluation. Likewise, item 11 (Were the outcome measures (dependent variables) clearly defined, valid, reliable, and implemented consistently across all study participants?) was rated “Yes” because the minimal requirement was agreed to be a standardized measure of outcome.

We organized the outcomes studied into the following categories: mortality, structural/physiological, treatment and care, medical (conditions/symptomatology), psychiatric disorders, sleep-related, cognitive functioning, functional, disability, social participation, work-related, and life satisfaction. We grouped the results according to injury severity (mild, moderate-to-severe, severe, and all severities) and post-injury phase (acute or chronic) at outcome assessment. Finally, to capture and interpret other characteristics that intersect with and contribute to sex/gender effects in the study results, we monitored the inclusion of social equity variables, including place of residence, race, ethnicity, culture, language, occupation, religion, education, socioeconomic status, social capital via human capital, and functioning within the family and/or other social groups (47). We extracted and reported all other studied variables related to the outcomes of interest.

### Presenting the Data

We anticipated limited sex/gender-specific hypotheses in the TBI literature, unequal representation of male and female persons, (48, 49) and differences in their personal and clinical characteristics (i.e., comorbidities), mechanisms, and injury severities, (50, 51) which would influence the observed associations' statistical power. We also expected that heterogeneity in the measures used, the timing of post-injury assessments, the study designs, and the quality of the studies would preclude quantitative compilation of sex/gender effects to understand the average magnitude and direction of the effects on different outcomes. As such, a meta-analysis, in its classic form, was not appropriate (52). We summarized the evidence and presented an overview of the findings across the 12 outcome categories according to injury severity and post-injury phase. We also organized and presented the results according to the approach taken to investigate sex/gender, using forest plots to depict sex/gender differences in outcomes reported as odds, risk, and hazard ratios with confidence intervals. Studies that offered β coefficients and partial R^2^ were depicted in pie charts, showing the contributions of different variables, including sex/gender, to the outcome. Finally, studies that stratified results by sex/gender, as opposed to controlling for these variables, were reported on separately.

## Results

### Study Characteristics

Supplementary Table 4 summarizes the study characteristics, population information, predictors, outcomes, statistical methods, and results. Supplementary Table 5 reports the results in succinct format and depicts the social equity parameters studied alongside sex/gender. The 58 chosen studies contained a total of 1, 265, 955 participants with TBI: 657, 009 had mild TBI (51.9%), 149, 828 had moderate TBI (11.8%), 38, 853 had severe TBI (3.1%), and the remaining 420, 265 (33.2%) were undetermined, as these participants came from studies that combined all severities. The percentage of male persons across the samples ranged from 47.4% (53) to 95% (54) with a mean 67.1% ± 9.9%. No studies reported inclusions of non-binary individuals. The participant ages ranged from 16 (55) to 85 years, (56) with a mean 46.52 ± 16.18 years across all studies. The vast majority of studies (95%) were quantitative, and two were qualitative (57, 58).

Gender Inequality Index ranks, methods, and outcome categories by injury severity.

All but two (53, 59) of the 58 studies were conducted in countries with very high human development indexes. Table 1 summarizes the GII rankings and outcomes. The GII ranking distribution varied: seven studies (60–66) were from countries with the highest gender equality rankings (GII rank <10: the Netherlands, Sweden, Norway, Finland, and Germany); and 28 (54–56, 58, 59, 67–89) were from a country with the lowest gender equality ranking (GII rank 43: United States). The remaining studies (57, 90–103) were based in Singapore, Spain, Austria, South Korea, and Canada (GII rank 10–19); Japan and Australia (29, 104–107) (GII rank 20–29); and Taiwan and China (GII rank 30–39) (53, 59).

**Table 1:** Gender Inequality Index ranks for studies investigating sex/gender's effect on outcomes of interest that considered age and TBI severity.

**Index rank**	**Studied outcome**												
	**Mortality**	**Structure/** **physiology abnormality**	**Treatment and care**	**Medical**	**Sleep-related**	**Psychiatric disorders**	**Disability**	**Work-related**	**Social participation**	**Life satisfaction**	**Cognitive functioning**	**Global functional outcome**	**OVERALL**
1–9				1ND		1ND		4ND		1ND		3ND	10 ND
10–19	4ND	1M^+^1F^+^	2M^+^ 1ND	1M+1F+1ND	2ND		1ND		1ND	1ND		1M^+^2ND	5M^+^ 2F^+^ 13ND
20–29	1M^+^ 1ND	1M+1ND								1M+	1F+ 2ND	1M+	4M^+^ 1F^+^ 4ND
30–39		1M+		1F+									1M^+^ 1F^+^
40–49	1M^+^ 3F^+^ 3ND	1M^+^2F^+^2ND	1M^+^ 1F^+^ 1ND	2M^+^2ND		1M^+^1ND	1M^+^ 1ND	1M^+^1F^+^	2M^+^ 1ND	1M^+^2ND	4M^+^ 2F^+^ 1ND	2M^+^ 3ND	17M^+^ 9F^+^ 17ND
Total studies	13	10	6	9	2	3	3	6	4	6	10	12	84

*Note. “+” indicates a more favorable outcome. M, male; F, female; ND, no difference; TBI, traumatic brain injury*.

Some studies assessed the impact of sex/gender on the outcome(s) of interest by testing the significance of sex using modeling techniques. They followed the hypothesis that if the relationship between the variables of interest (mostly TBI-related) and the outcome differs for male and female persons, sex adjustment provides an estimate of the average relationship if sex is held constant. Others tested sex as an interaction term with other variables of interest in relation to an outcome, and the rest disaggregated the data by sex, providing insights into how exposures may differ for male and female persons. Table 1 presents the outcomes grouped into 12 categories by GII, post-injury phase, and injury severity: mortality, (29, 67, 83–86, 97, 98, 101). (88, 102) structure/physiology abnormality, (56, 59, 72, 75, 95, 96, 107, 108) treatment and care, (56, 81, 102, 103) medical, (53, 54, 65, 71, 72, 92, 99, 104) sleep-related, (91, 93) psychiatric disorders, (62, 76) cognitive functioning, (55, 68, 81, 89, 100, 106, 109) global functioning, (29, 63, 65, 79, 80, 97, 101) disability, (79, 81, 94) work-related, (60, 61, 65, 66, 77) social participation, (55, 81, 90) and life satisfaction (65, 81, 82, 89, 105).

### The Approach to Capture Sex/Gender Effects

**Figure 2:**
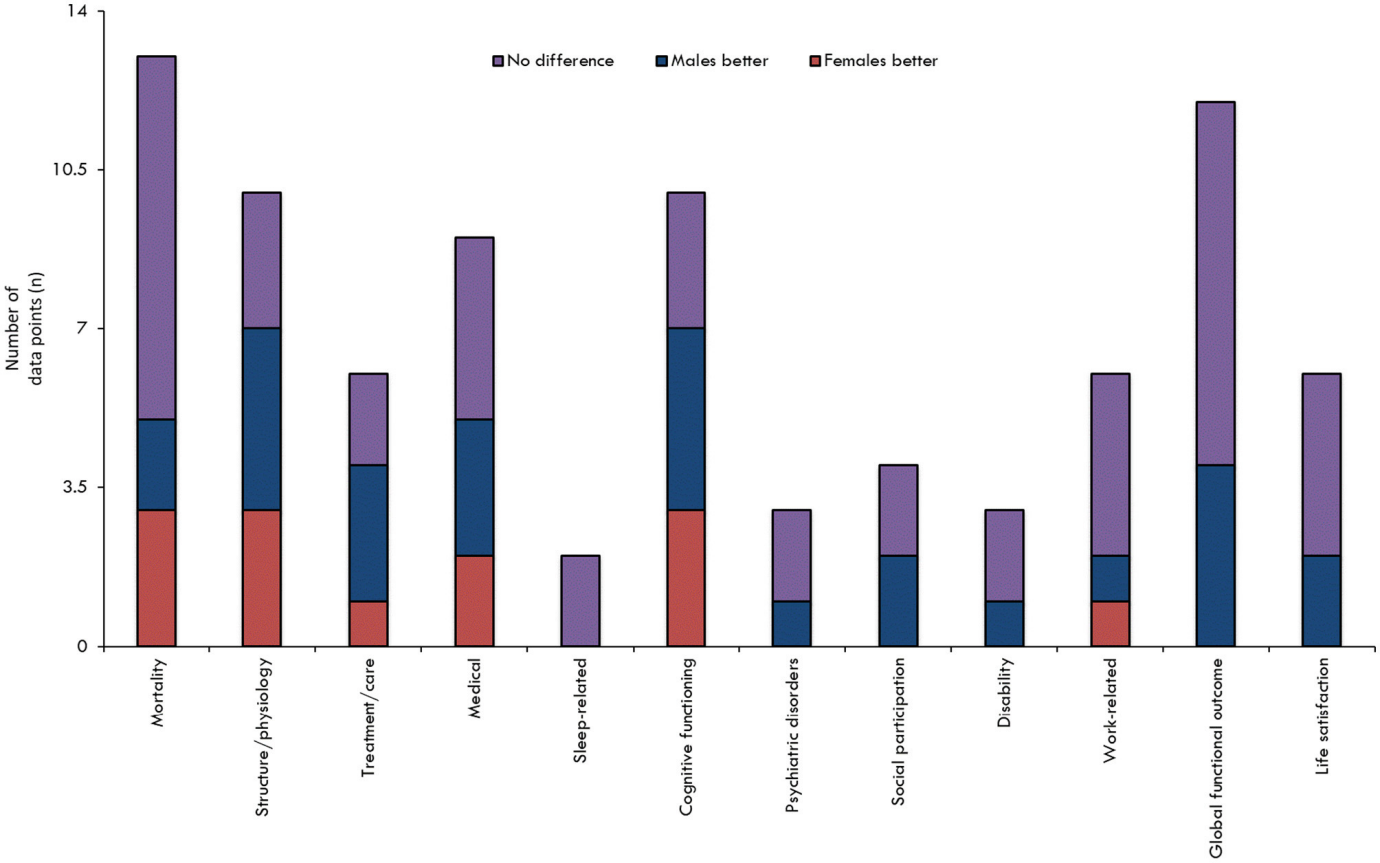
Findings overview overall, by outcome. The results are stratified according to assessed outcome. The height of the bar is only an indicator of assessment frequency at a certain point in time.

**Figure 3:**
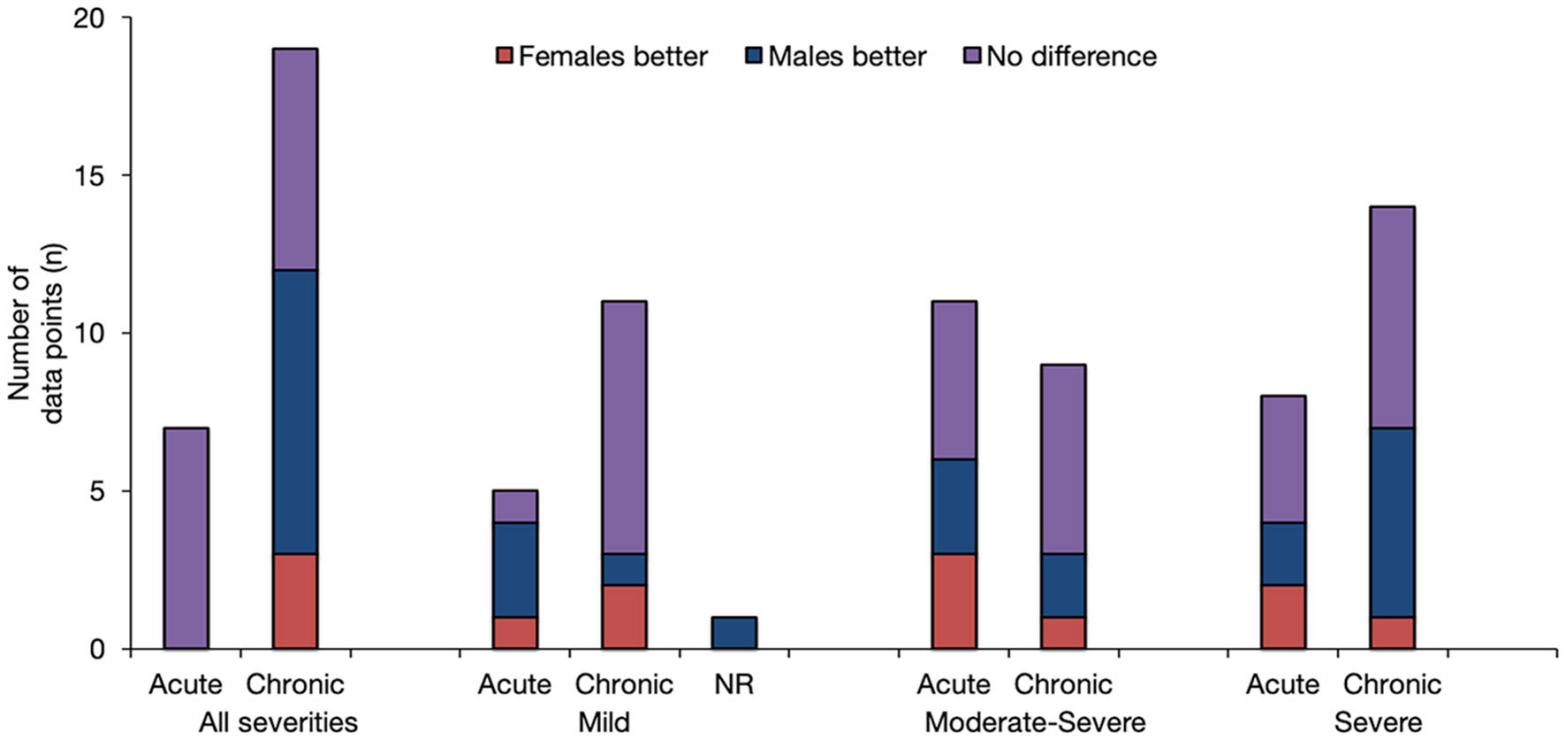
Overview of Findings by injury severity and post-injury phase. The results are stratified according to injury severity, and whether the baseline outcome assessment was conducted prior to, at, or following the 3-month post-injury period. The height of the bar is only an indicator of assessment frequency at a certain point in time.

When all the results pertaining to sex/gender and TBI outcomes were reviewed in their entirety (Figures 2, 3), it was observed that most studies that included the relevant variables observed no difference between male and female persons with respect to outcomes. The second most common result showed that male persons fared better than female persons, particularly in the chronic phase post-injury and within the more severe TBI cohorts.

**Figure 4:**
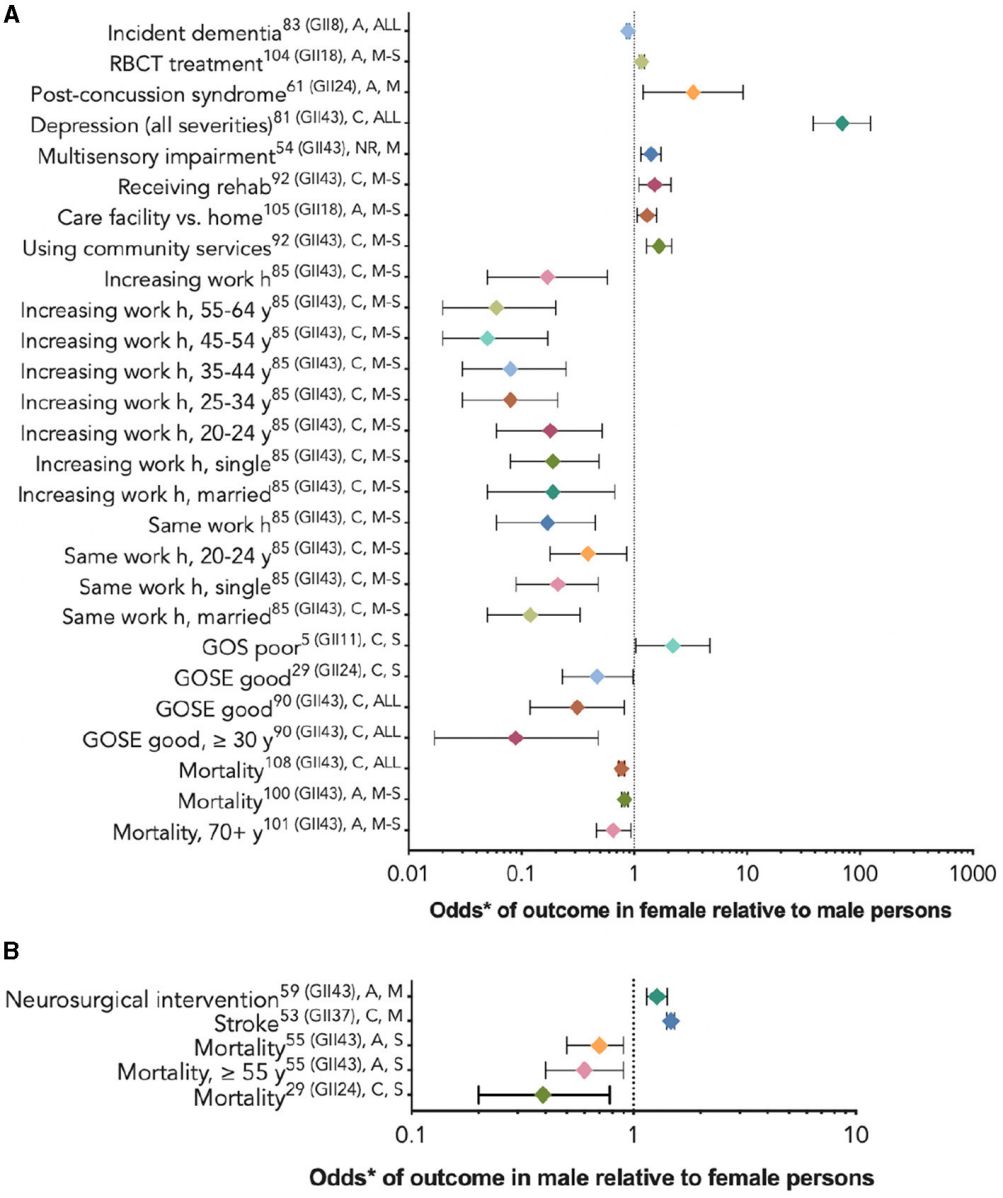
Statistically significant sex/gender-related results reported as OR/RR/HR (CI). *The RBCT treatment and dementia outcome studies reported risk ratios **(A)**, the stroke outcome studies reported hazard ratios **(B)**, and the rest reported odds ratios. **(A)** The outcome odds for female relative to male persons. **(B)** The outcome odds for male relative to female persons. The error bars represent a 95% confidence interval. The superscript letters indicate the GII rank of the country where the study was conducted (lower rank indicates lower gender inequality), the post-injury phase (A, acute; C, chronic; and NR, not reported), and injury severity (M, mild; M-S, moderate-to-severe; S, severe; and ALL, all severities). GII, Gender Inequality Index; RBCT, red blood cell transfusion.

**Figure 5:**
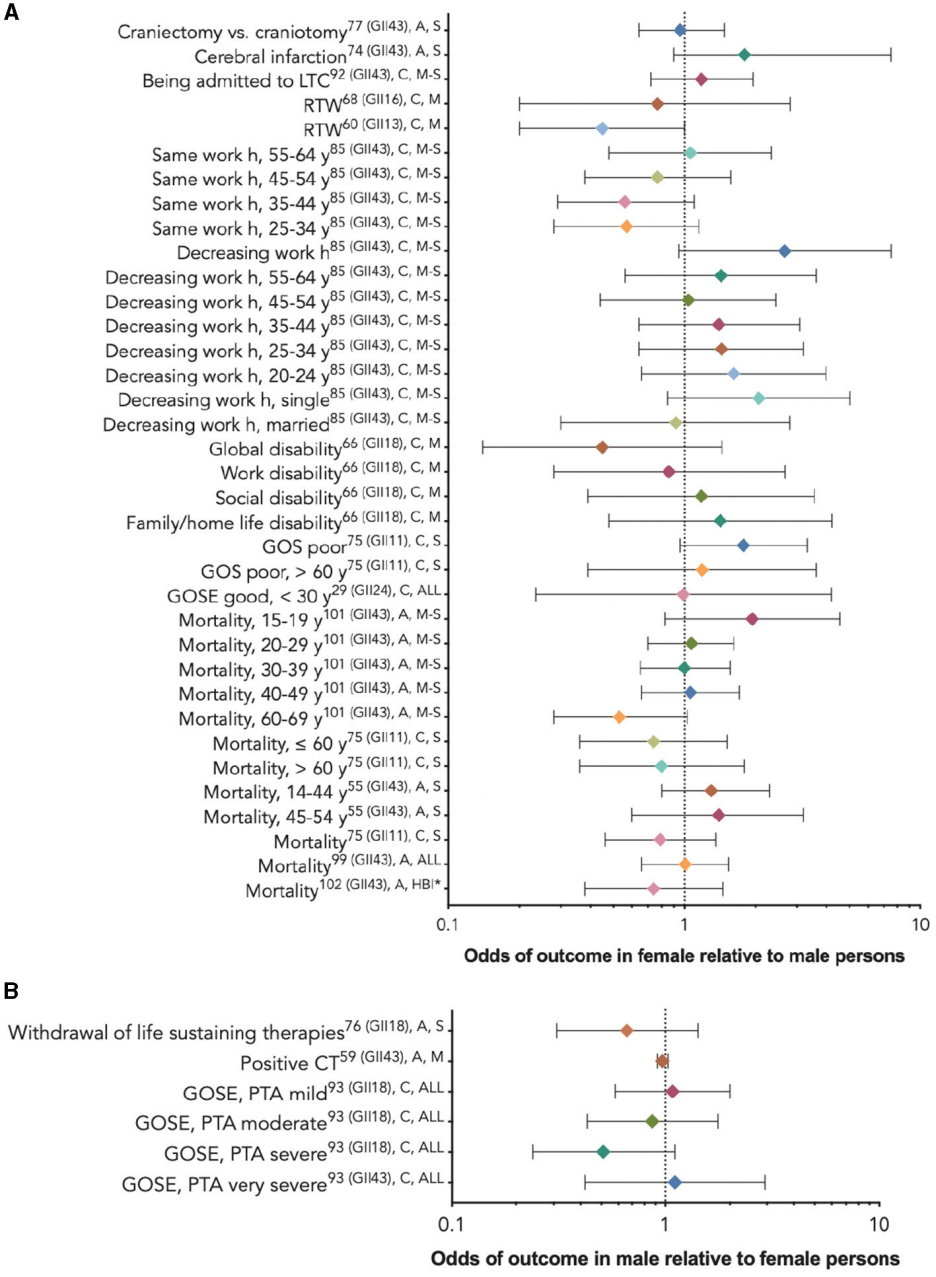
Non-statistically significant sex/gender-related results reported as OR/RR/HR (CI). **(A)** The outcome odds for female relative to male persons. **(B)** The outcome odds for male relative to female persons. The error bars represent a 95% confidence interval. The superscript letters indicate the post-injury phase (A, acute; C, chronic; and NR, not reported) and injury severity (M, mild; M-S, moderate-to-severe; S, severe; ALL, all severities; and HBI, hemorrhagic brain injury). *No reference groups in the sex/gender analysis (86).

Twenty-nine studies (29, 53–56, 60, 61, 64, 67, 71, 72, 76, 77, 80, 81, 83–86, 90, 92, 94, 95, 97–99, 102–104) used multivariate analyses to assess which variables, including sex/gender, were associated with their chosen outcomes. Nineteen studies (29, 53–56, 67, 76, 77, 80, 81, 84, 85, 94, 95, 97, 99, 102–104) compared outcomes between female persons and male persons, including a study of response to red blood cell transfusion (RBCT), (102) which reported risk ratios, a dementia outcome study, (99) and a stroke outcome study, (102) which provided hazard ratios; the rest provided odds ratios. Five studies (29, 67, 77, 95, 97) reported that the relationship between sex/gender and the outcome varied by age or injury severity. Figure 4 provides these results based on outcome category, phase, and injury severity (detailed in Table 2). The significant findings suggest that female persons with TBI are more likely to sustain a comorbid neck injury in addition to a concussion, (95) receive RBCT treatment (102) and rehabilitation services, (81) be discharged to a care facility vs. their home, (103) use community services, (103) decrease their working h or stop working after the injury, (77) and experience depression, (81) multi-sensory impairment, (54) post-concussive syndrome, (104) and poor outcomes. (80) Male persons have a greater relative risk of dementia (99) and stroke (53) and are more likely to receive neurosurgical intervention. (56) Male persons also are more likely to have favorable functional outcomes than female persons (Figure 5) (29). Further evidence is provided in Table 2, Supplementary Tables 4, 5. The statistical power of the studies that reported a difference between the sexes in the studied outcomes varied greatly (Table 2).

**Table 2:** Review findings: differences in outcomes of male and female persons after TBI.

**Outcomes**	**Female patients fare better**	**Male patients fare better**	**No difference**
Mortality	Berry et al. (84) (GII 43)^A, M−S^ (GII 18) (time to dementia (N = 72, 294) Davis et al. (85) (GII 43) 50–59 years, 70+ years^A, M−S^ (GII 18) (time to dementia (N = 13, 437) Selassie et al. (88) (GII 43)°ver 12 years post-injury, ALL (N = 33, 695)	Ottochian et al. (67) (GII43) ≥ 55 years^A, S^ (N = 557)Ponsford et al. (29) (GII 24), >60 years^C, S^ (N = 36)	Boutin et al. (102) (GII 18)^A, M−S^ (N = 7, 062) Leitgeb et al. (101) (GII 14)^A, M−S^ (N = 439) Davis et al. (85) (GII 43), 15–49 years^A, M−S^ (N = 13, 437) Côte et al. (98) (GII 18) (withdrawal of life sustaining therapies)^A, S^ (N = 720) Albrecht et al. (83) (GII 43)^A, ALL^ (N = 1, 320) Fortuna et al. (86) (GII 43)^A, ALL^ (N = 416) Ng et al. (97) (GII 11)^C, S^ (N = 672) Ponsford et al. (29) (GII 24), ≤ 60 years^C, S^ (N = 193)
Structure/function abnormality	Clond et al. (75) (GII 43) (SBP changes)^A, M−S^ (N = 3, 025) Arellano-Orden et al. (96) (GII 15) (RBCT response)^A, S^ (N = 88) Wagner et al. (108) (GII 43) (oxidative damage markers)^A, S^ (N = 68)	Wagner et al. (108) (GII 43) (DOPAC)^A, S^ (N = 68)Schönberger et al. (107) (GII 24) (viable volume)^C, ALL^ (N = 98)Wang et al. (59) (GII 36) (abnormal functional connectivity in the brain)^A, M^ (N = 54)Sutton et al. (95) (GII 18) (neck injury comorbidity 5–49 years)^A, M^ (N = 90, 175)	Kisat et al. (56) (GII 43) (+CT scan)^A, M^ (N = 24, 424) Wagner et al. (108) (GII 43) (HVA)^A, S^ (N = 68) Schönberger et al. (72) (GII 24) (lesion volume)^C, ALL^ (N = 98)
Treatment and care	Kisat et al. (56) (GII 43) (neurosurgical intervention)^A, M^ (N = 3, 476)	Brown et al. (103) (GII 18) (discharge destination)^A, M−S^ (N = 3, 480)Boutin et al. (102) (GII 18) (RBCT treatment)^A, M−S^ (N = 1, 991)Mellick et al. (81) (GII 43) (going to rehab, using community services)^C, M−S^ (N = 392)	Boutin et al. (102) (GII 18) (LOS, discharge destination)^A, M−S^ (N = 7, 062) Mellick et al. (81) (GII 43) (LTC)^C, M−S^ (N = 108)
Medical	Lee et al. (53) (GII 37) (stroke)^C, M^ (N = 24, 905) Mollayeva et al. (99) (GII 18) (incident dementia risk)°ver 5 years post-injury, ALL (N = 712, 708)	Pogoda et al. (54) (GII 43) (MSI)^NR, M^ (N = 9, 998)Meares et al. (104) (GII 24) (PCS)^A, M^ (N = 90)Mollayeva et al. (99) (GII 18) (time to dementia)°ver 5 years post-injury, ALL (N = 712, 708)	Rush et al. (72) (GII 43) (craniectomy vs. craniotomy)^A, S^ (N = 302) Tawil et al. (71) (GII 43) (cerebral infarction)^A, S^ (N = 384) Renner et al. (65) (GII 9) (pituitary insufficiency)^A, ALL^ (N = 427) Mollayeva et al. (92) (GII 18) (chronic pain)^C, M^ (N = 94)
Sleep-related			Mollayeva et al. (91) (GII 18) (insomnia)^C, M^ (N = 94) 2) Mollayeva et al. (93) (GII 18) (sleep stage deviations)^C, M^ (N = 39)
Psychiatric disorders		Glenn et al. (76) (GII 43) (all depression)^C, ALL^ (N = 41)	Glenn et al. (76) (GII 43) (moderate/severe depression)^C, ALL^ (N = 41) Harrison et al. (62) (GII 4) (schizophrenia, psychosis)^C, S^ (N = 2, 274)
Disability		Mellick et al. (81) (GII 18) (physical independence)^C, ALL^ (N = 1, 802)	Dahdah et al. (GII 43)^A/C, M−S^ (N = 5, 505) Mollayeva et al. (94) (GII 18)^C, M^ (N = 92)
Work-related	Corrigan et al. (77) (GII43, work hours)^C, M−S^ (N = 1, 257)	Corrigan et al. (77) (GII 43, stopped working)^C, M−S^ (N = 1, 417)	Renner et al. (65) (GII 9) (employment)^A, ALL^ (N = 427) de Koning et al. (60) (GII 3) (RTW)^C, M^ (N = 316) Vikane et al. (61) (GII 6) (RTW)^C, M^ (N = 151) van der Horn et al. (66) (GII 3)^C, ALL^ (N = 242)
Social participation		Gerhart et al. (55) (GII 43) (cognitive independence, mobility, occupation)^C, S^ (N = 1, 802)Mellick et al. (81) (GII 43) (CI)^C, ALL^ (N = 1, 802)	Mollayeva et al. (90) (GII 18) (CI)^C, M^ (N = 94) Gerhart et al. (55) (GII 43) (physical independence, social integration, economic self-sufficiency)^C, S^ (N = 1, 802)
Life satisfaction		Mellick et al. (81) (GII 43) (health perception)^C, ALL^ (N = 1, 802)Takada et al. (105) (GII 21) (physical function)^C, ALL^ (N = 12)	Renner et al. (65) (GII9) (living situation)^A, ALL^ (N = 427) Williamson et al. (82) (GII 43) (LS)^C, M−S^ (N = 3, 157) Saban et al. (89) (GII 43) (LS)^C, S^ (N = 287) Takada et al. (105) (GII 21) (QOL mental and role/social)^C, ALL^ (N = 12)
Cognitive functioning	Tsushima et al. (109) (GII 43), <30 years (two neuropsych measures)^C, M^ (N = nr) Saban et al. (89) (GII 43) (FIM cog)^C, S^ (N = 287) Eramudugolla et al. (86) (GII 24) (verbal ability, 20s cohort)^C, ALL^ (N = 2, 077)	Tsushima et al. (109) (GII 43) ≥ 30 years (two neuropsych measures)^C, M^ (N = nr)Gerhart et al. (55) (GII 43)^C, S^ (N = 1, 802)Myrga et al. (68) (GII 43)^C, S^ (N = 193)Mellick et al. (81) (GII 43)^C, ALL^ (N = 1, 802)	Jung et al. (100) (GII 22) (cog func)^A−C, ALL^ (N = 162) Tsushima et al. (109) (GII 43) (cog func)^C, M^ (N = 102) Eramudugolla et al. (106) (GII 24) (verbal ability, 40s and 60s cohort)^C, ALL^ (N = 4, 256)
Global functional outcome		Dahdah et al. (79) (GII 43) (FIM)^A, M−S^ (N = 5, 505)Ng et al. (97) (GII 11) (GOS, <60 years)^C, S^ (N = nr)Ponsford et al. (29) (GII 24) (GOSE)^C, S^ (N = 229)Kirkness et al. (80) (GII 43) (GOSE, 30+ years) ^C, ALL^ (N = 157)	Renner et al. (65) (GII 9) (GOS)^A, ALL^ (N = 427) Dahdah et al. (79) (GII 43) (GOSE)^C, M−S^ (N = 5, 505) Forslund et al. (63) (GII 6) (GOSE)^C, M−S^ (N = 105) Leitgeb et al. (101) (GII 14) (GOS)^C, M−S^ (N = 439) Ng et al. (97) (GII 11) (GOS, 60+ years) ^C, S^ (N = nr) Saban et al. (89) (GII 43) (FIM motor)^C, S^ (N = 287) Östberg & Tenuvuo (64) (GII 8) (GOSE)^C, ALL^ (N = 689) Kirkness et al. (80) (GII 43) (GOSE, <30 years) ^C, ALL^ (N = nr)

*A, acute; ALL, all injury severity; C, chronic; CI, community integration; CT, computed tomography; DOPAC, dihydroxyphenylacetic acid; FIM, functional independence measure; GOSE, Glasgow Outcome Scale Extended; M, mild TBI; MSI, multisensory impairments; M-S, moderate-to-severe traumatic brain injury; nr, not reported; LS, life satisfaction; LTC, long-term care; PCS, post-concussion syndrome; S, severe traumatic brain injury; SBP, systolic blood pressure; QOL, quality of life*.

**Figure 6:**
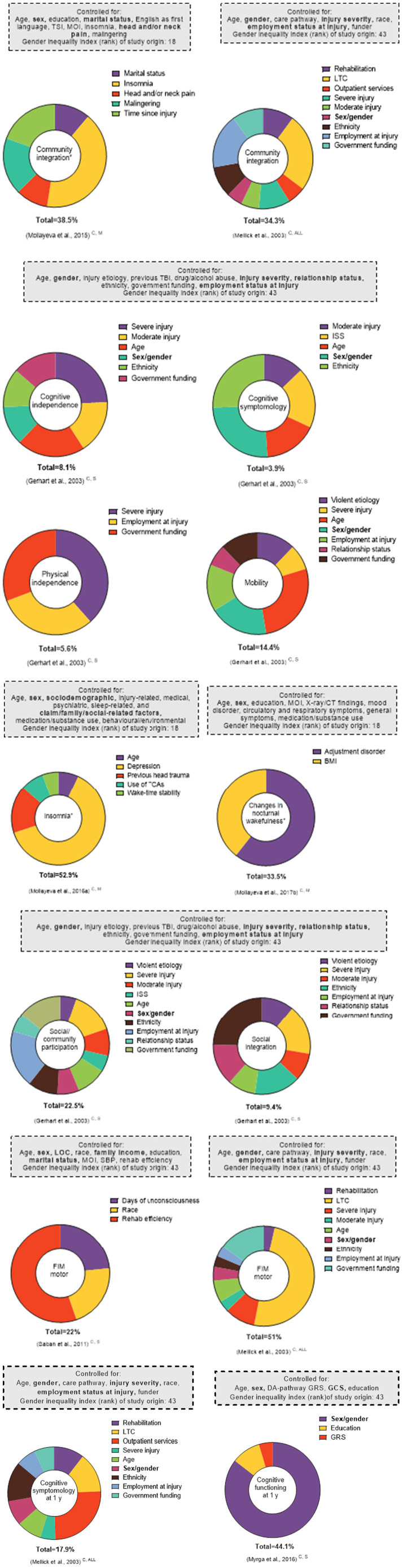
Effect sizes of explained factors/variance investigated in relation to post-TBI outcomes in multivariate regression analyses. This figure represents select findings for comparative purposes (all outcomes available in Supplementary Figure 5). All analyses included sex/gender. For the * outcomes, the proportions correspond to the partial R^2^. For all other outcomes, the proportions correspond to the β coefficients. Total corresponds to the total variance explained by the model (R^2^). Only statistically significant factors are included as explaining variance, given that the cutoff *p*-values were not consistently reported. The superscript letters indicate post-injury phase (A, acute; C, chronic; and NR, not reported) and injury severity (M, mild; M-S, moderate-to-severe; S, severe; and ALL, all severities). The bolded factors in the “Controlled for” boxes indicate those that have been linked to sex and/or gender.

Sixteen studies, among which some utilized subgroup analyses by age or injury severity, found insignificant associations between sex/gender and the outcomes of interest (29, 56, 60, 61, 64, 67, 71, 72, 77, 81, 83, 85, 86, 90, 97, 98). Figure 6 provides these results based on outcome category, phase, and injury severity (detailed in Supplementary Tables 4, 5). The insignificant findings suggest no differences between the sexes within the full TBI cohort, within certain age groups receiving craniectomies vs. craniotomies, (72) or with regard to withdrawing from life-sustaining therapies (98); obtaining positive results on a computed tomography scan (56); cerebral infarction risk (71); long-term care admission (81); return to work (60, 61); perceiving more severe work, social, or family disability (94); or post-injury mortality (67, 83, 86, 97). The statistical power of the studies that did not report a difference between the sexes in the studied outcomes varied greatly (Table 2).

### Sex/Gender's Effect via β Coefficients or Partial R^2^

Twelve studies reported the significance of sex/gender variables in the multivariate linear regression modeling of several outcomes, including medical, (92) structure/function, (73, 107) treatment and care, (102) sleep-related, (91) social participation, (55, 81, 90) disability, (79, 81, 94) cognitive functioning, (89) and life satisfaction (105). Nine studies investigating 17 outcomes, falling under structure/function, social participation, disability, cognitive functioning, and life satisfaction, (55, 68, 74, 79, 81, 89, 102, 105, 107) found significant associations between sex/gender and the outcome of interest. All but two (105, 107) stemmed from countries with the highest GII rankings. In multivariate regression analyses, the sex/gender effect size or variance explained in relation to post-TBI outcomes varied relative to the other variables considered in the modeling process. In six studies investigating 14 outcomes, (79, 89, 90, 92, 107, 108) the association between sex/gender and the studied outcome was not statistically significant after covariate adjustments, including adjustment for social equity parameters (detailed in Supplementary Tables 4, 5). Three of these studies, conducted in the United States, focused on disability at discharge from rehabilitation, (79) the motor functional independence measure (FIM) score, (89) physical independence, (55) and certain markers of oxidative damage (73). Another three, stemming from countries with intermediate GII rankings, focused on white matter and viable lesion volumes, (107) sleep architecture, (93) and chronic pain (92).

The findings highlight three possible trends (Figure 6). First, within a study (55) that investigated related outcomes (i.e., social integration, social/community participation, or mobility and physical independence) and used various explanatory social equity factors in the modeling process, the significance of the sex/gender association was either present or absent. Second, in studies that focused on related outcomes in persons with different injury severities [community integration (81, 90) and motor FIM (81, 89)] and included several social equity variables, the effect of sex/gender was again either present or absent. Third, two studies from countries with the same GII ranking, (55, 81) investigating a related outcome (cognitive symptomatology) and minimally accounting for social equity variables (employment at injury only), reported a significant sex/gender effect with respect to the studied outcome (Supplementary Figure 1).

### Sex/Gender Variable in Interaction Terms

Five studies (77, 85, 87, 95, 109) examined the interaction of sex/gender and age in relation to marital status (77) and menopausal status (85) (Supplementary Tables 3, 4). One reported a sex-by-age effect on the Trail Making A Test results, (109) another found that decreasing employment hours or stopping work post-injury was most evident for married female persons with TBI, (77) and a third study discovered an increased risk of comorbid neck injury in females between 5 and 49 years of age (not before or after), with the interaction between sex and age following a non-linear trend. (95) Another finding highlighted that the divergence in mortality was more favorable for female persons around menopause (85).

### Studies Disaggregating Data by Sex/Gender

Seven studies (59, 62, 69, 75, 87, 92, 99) examined differences between the sexes in covariates of the outcomes by stratifying their cohorts according to sex (Table 3). When observing the studied variables' effects on the outcomes of interest within the two sex groups, it is apparent that some variables exhibited less variation in the strength of their associations with certain outcomes. For example, many studied variables were associated with discrete outcomes, such as mortality, dementia, pneumonia, non-affective psychosis, and hospital readmission, in both sexes with moderate-to-severe TBI (62, 75, 87, 99). Other variables, such as insomnia severity, post-traumatic stress disorder, and age, were connected to the outcome in male or female persons only, (59, 69, 92) although these variations emerged in persons with mild TBI only.

**Table 3:** Results from studies analyzing the relationship between outcome and variables on male and female persons separately.

**Outcome**	**Male patients**	**Female patients**
Abnormal functional connectivity in resting-state brain networks (59) (GII 36)^A, M^	-Insomnia severity (+)	None
Alcohol use disorder (69) (GII 43)^NR, M^	-PTSD (+) -Age (–)	None
Chronic pain (92) (GII 18)^C, M^	-Explosion MOI (+) -Fall from height MOI (+) -Anxiety (+) -Insomnia (+) -Tension with insurer (+) **-Employment (–)** **-English not being the first language (–)**	**-More than HS education (–) -Working less than 40 h/week (–)** -Daytime sleepiness (–) -n of SRBD risk factors (+)
^*^Dementia incident (99) (GII 18)^C, ALL^	-Concussion vs. mild (+) -Comorbid SCI (+) -Younger age (+) **-Neighborhood income, higher (–)** -Cerebrovascular disease (+) -Ischemic heart disease (–) -Diseases of arteries, arterioles, and capillaries (+) -Atrial fibrillation (–) -Heart failure (+) -Tobacco smoking (+) -Hyperlipidemia (+) -Diabetes mellitus (+) -Depression (+) -Sleep disorder (+)	-Concussion vs. mild (+)-Moderate TBI vs. mild (+)** -Neighborhood income, higher (–)** -Cerebrovascular disease (+ -Ischemic heart disease (–) -Heart failure (+) -Tobacco smoking (+) -Diabetes mellitus (+) -Depression (+) -Vision impairments (–)-Sleep disorder (+)
-Pneumonia (75) (GII 43) ^A, M−S^	-ISS ≥ 16 (+) -GCS ≤ 8 (+) -SBP ≥ 160 mmHg (+) -Advanced age (+)	-ISS ≥ 16 (+) -GCS ≤ 8 (+) -SBP ≥ 160 mmHg (+)
-Hospital readmission (87) (GII 43) ^A, ALL^	-Older age (+)	-Older age (+)
Non-affective psychosis (62) (GII 4) ^C, S^	-Severe head injury (+)	-Severe head injury (+)
Mortality (75) (GII 43) ^A, M−S^	-ISS ≥ 16 (+) -GCS ≤ 8 (+) -SBP <90 mmHg (+) -SBP ≥ 160 mmHg (+) -Advanced age (+)	-ISS ≥ 16 (+) -GCS ≤ 8 (+) -SBP <90 mmHg (+) -Advanced age (+)

*Social equity parameters in bold. *

* *Results of a simplest dementia model. Models accounting for TBI severity interaction with age highlighted severe TBI leading to an increased rate of developing dementia compared with mild TBI, for younger age groups and male patients to a greater extent than for older and for female patients. (+) positive direction of association with studied outcome; (–) negative direction of association with studied outcome. A, acute; ALL, all injury severity; C, chronic; GCS, Glasgow Coma Scale; ISS, injury severity score; HS, high school; MOI, mechanism of injury; NR, not reported; PTSD, post-traumatic stress disorder; SCI, spinal cord injury; SBP, systolic blood pressure; SRBD, sleep-related breathing disorder; TBI, traumatic brain injury; GII, Gender Inequality Index*.

### Sex/Gender and Other Social Equity Variables via Progress

The reviewed literature revealed socioeconomic status, occupation, and education as the three most frequently studied social equity variables. The effect of socioeconomic status, alongside sex/gender, was assessed in 16 studies (53, 55, 56, 62, 70, 72, 77, 79, 81, 82, 87, 88, 92, 94, 95, 99); followed by education, assessed in 15 studies (54, 59, 60, 63, 64, 68, 77, 79, 82, 89, 91, 93, 94, 100, 109); race/ethnicity/culture/language, assessed in 14 studies (55, 56, 70, 72, 77, 79, 81–83, 88–90, 92, 94); occupation, examined in 12 studies (54, 55, 60, 63, 65, 77, 79, 81, 82, 90–92); social capital, considered in 11 studies (55, 63, 77, 79, 82, 89–92, 94, 105); and place of residence, considered in eight studies (53, 62, 65, 72, 81, 87, 95, 100). Religion was not investigated as a factor related to TBI outcome. PROGRESS variables alongside sex/gender effects are depicted in Supplementary Table 5.

### Qualitative Studies

We found one study investigating perceived gender inadequacy (17) and another tackling gender role/norm expectations (57) in men and women with TBI. The first was conducted in the United States (GII rank: 42) and the second in Canada (GII rank: 18). Regarding the former, the results revealed that male persons with TBI living in a residential facility expressed more feelings of gender inadequacy post-injury and were heavily focused on traditional activities before and after the injury to define and support their roles (17). In contrast, women with TBI relied more on cross-gender activities and were able to maintain more of their pre-injury activities. The Canadian study conducted interviews with men and women with TBI undergoing rehabilitation, due to a recent mild or moderate-to-severe TBI (57). The pre-injury gendered roles and responsibilities of both sexes were altered post-injury. Across injury severities, men felt emasculated, while women felt guilty, due to their hindered ability to return to their gendered pre-injury roles and responsibilities. For both sexes, this distress was central in their discussions on the lack of solutions, attempts to find solutions, feelings of helplessness, and implicit awareness of the relationship between sex/gender and their emotional distress. The need to reflect on socially constructed gender views differed between male and persons, attributed to differences in age, ethnicity, and other social equity parameters.

## Discussion

### Summary of Main Findings

Although this synthesis precludes the formation of uniform conclusions regarding the effect of sex/gender on core TBI outcomes, the results provide an opportunity for a unique and complex discussion that has clinical and research implications pertaining to biological (sex) and sociocultural (gender) considerations. The strongest emerging pattern was that differences between male and female persons in medical, work, life satisfaction, and functional outcomes were less frequent across injury severities, as the GII of the study's country of origin improved. In a world characterized by different value systems and cultures, it is of special importance to consider whether there are universal principles related to sex/gender, because if there are, we must determine how we can apply them in specific TBI contexts.

Several arguments suggested the potential role of gender inequality in widening the outcome disparities between the sexes with TBI, with significantly different cultural perspectives and social roles/responsibilities impacting the experience of injury. As a social process, gender defines women, often with emphasis on the domestic sphere and related tasks (e.g., motherhood and providing care), (110, 111) and men with an emphasis on courage, emotional restraint, risk-taking, and pursuit of success (112, 113). Thus, researching the effect of sex/gender in TBI requires a model that considers the influences of belief systems in various cultures, and social roles, responsibilities, and relationship perceptions (111, 113, 114).

Important biological considerations also emerged in this synthesis (49). Although males and females share about 99% of their genetic material, the remaining genotypic differences account for large phenotypic variations between the sexes (115, 116). One study found that females between the ages of 5 and 49 had a greater likelihood of sustaining comorbid neck injuries in acute concussion, which can reasonably be linked to phenotypic variation between the sexes (95). Similarly, the cellular changes brought about by sex hormones may explain the greater relative risk of dementia in male persons with TBI, after controlling for all other known risks and protective factors (99). However, when the data were disaggregated by sex, the risk and protective factors differed between the sexes, and female persons were found to develop dementia earlier than male persons, all other risk and protective factors equal (99). These results indicate that vulnerability to adverse outcome may depend not only on biological susceptibility but also on the degree of behavioral capacity to cope with TBI impairment. Therefore, stratifying data by sex is a natural step for advancing knowledge on sex/gender differences relevant to the studied outcomes.

### Implications for Research and Policy

Based on this review, we can make several recommendations for research and healthcare policy. First, studies examining the effect of sex/gender on various TBI outcomes should incorporate methodologies that can best capture these effects. Most studies included in this review adjusted their results for sex, several provided sex-stratified results, and a few reported the interaction effect between sex and other factors on the studied outcomes. They were primarily motivated to use and select covariates for statistical adjustment to increase the study's internal validity by correcting the data and eliminating confounders (117, 118). However, no adjustments should be undertaken until subgroup-specific rates have been studied carefully (119). Despite these caveats, this adjustment procedure dominated the reviewed studies, making it impossible to determine what was actually observed in the sex-specific strata. The pooling of data across sexes not only assumed no difference between sexes but also prevented the researchers from testing an outcome's dependency on a participant's sex (120).

Second, most studies mainly provided sex-stratified results for descriptive purposes, as a way of comparing male and female persons by examining statistical significance. However, this results in two key biases: 1) uneven sizing of each sex cohort and 2) gathering participants from convenient intake procedures in clinical settings, rather than random selection. Female persons were underrepresented in most studies, potentially due to TBI being associated with young males, and this selective attention can alter the prevalence and incidence statistics, especially for younger females. Structural barriers may also limit younger females' full participation due to childbearing and parenting, gender norms, and access to research (22). Having both variances and unequal sample sizes affects type I error rates and creates difficulty in accounting for confounding variables (121).

Third, we sought to detect sex/gender effects by reviewing the results from the literature on male and female persons with TBI, across clinical and population-based samples. In the discovery phase, we extracted data on the effect of sex/gender independent of TBI severity and age on clinical and functional outcomes, from 58 different studies' cohorts capturing 1, 265, 955 participants with TBI. In the analysis phase, we mapped the results from the observational studies that showed statistically significant differences in either direction and no differences between the sexes, regardless of their sample size, whether low count, (105) or in the thousands (99) (Table 2). Although a larger sample size enabled the researchers to uncover smaller differences, and results had narrower confidence intervals, we are unable to attribute the observed results, whether significant or not, solely to statistical power. We conclude that the binary nature of the sex/gender variable (i.e., male = man; female = women) and unexplored PROGRESS variables were skewing the results in either direction.

A researcher's role in noting and interpreting sex/gender effects is equally significant. Irrespective of whether or not a difference between the sexes was observed in the outcomes, most researchers seldom made hypotheses concerning sex/gender effects or considered the numbers needed to uncover statistically significant associations. The likelihood of missing an important difference, and therefore of making a type II error, decreases as the sample size gets larger (121). Although hundreds of patients recruited by investigators in the studies that showed no differences between male and female patients with TBI on a number of outcomes (Table 2) may seem like a substantial number, for outcomes that are likely subject to interaction between sex/gender and other social equity parameters, even larger sample sizes are likely needed, based on some reasonable assumptions regarding main effects and interactions −16 times the sample size to estimate an interaction vs. that needed to estimate the main effect (122). Thus, it is important to bear in mind that when a study fails to reject the null hypothesis with regard to differences between the sexes, this only means that there is no evidence of a difference between male and female patients under specific comparison (123). This is different from concluding that outcomes for male and female patients with TBI are the same. A solid argument to retire statistical significance as a way of interpreting results, and to draw conclusions based on a more complete interpretation and standardized reporting of results, applies here (124).

Finally, the sex/gender-binary lens of the research summarized in this review [i.e., where two biological sexes (male/female) are considered to be associated with a specific gender identity (man/woman)] is expected to contribute to the contradicting evidence on the effect of sex/gender across the studied outcomes. Emerging evidence in neuroscience and behavioral sciences challenges this binary view (124) and calls for researchers to take a more inclusive approach to better understand sex/gender and the brain, and how that relates to outcomes. However, analytical methods that would allow study of non-binary gender variability in outcomes are yet to be developed, and this effort may be seen to be redundant in the realm of personalized medicine (125–127). On a positive note, sex/gender effects have started to attract significant attention in TBI research (13, 19, 111, 126, 127); and new policies have been developed to capture these effects (128–132).

### Limitations

Despite the strengths of our synthesis, this review does have limitations. Firstly, the data was limited, with inadequate accounts of social equity and gender factors and inadequate accounts of social equity and gender factors (norms, responsibilities, and relations), which impacted interpretation of the results. Further limitations stem from the inclusion criteria, which drew studies that included sex/gender along with TBI severity and age in their analyses of TBI outcomes. This decision was made to avoid selective reporting bias in case of any differences stemming from TBI severity and age on the studied outcomes. Nonetheless, residual confounding is possible, as variables other than TBI severity and age, which were not uniformly accounted for across the studies, may have contributed to the observed non-uniform sex/gender effects in studies that focused on similar outcomes.

An ongoing challenge was the inconsistent methods used to assess sex/gender effects. Most studies did not incorporate sex and gender in their hypotheses; and thus, reporting and discussing of sex/gender effects were limited or absent altogether, making our interpretations difficult and vague. Adding to these gaps in knowledge, recent research has advocated for inclusion of sex/gender as a non-binary variable, as differences exist within male and female populations in genetics, sex hormone cycles, and developmental stages, which could create variance in TBI exposure and outcomes (128).

In this review, we did not exclude studies on the basis of insufficient power for studying sex/gender effects, as most studies did not provide sample size justifications (Supplementary Files 3A,B). As a result of this missing information, we do not know whether the reported results were truly significant or not, or whether there was collinearity between sex/gender and other studied variables included in the statistical modeling. In addition, our last search for papers took place in September 2019, and the latency between the date of the search and publication of the review may introduce bias. This latency reflects the complexity of the review. Finally, despite our attempts to collect all relevant articles through a comprehensive search of six databases working alongside an information specialist, utilizing the published methods for different databases and interfaces, it is possible that some studies were missed due to indexing failures (not all the main concepts addressed in the articles appear as descriptors) and due to the possible lack of suitable descriptors for representing the concepts of sex and gender (130).

## Conclusion

**Figure 7:**
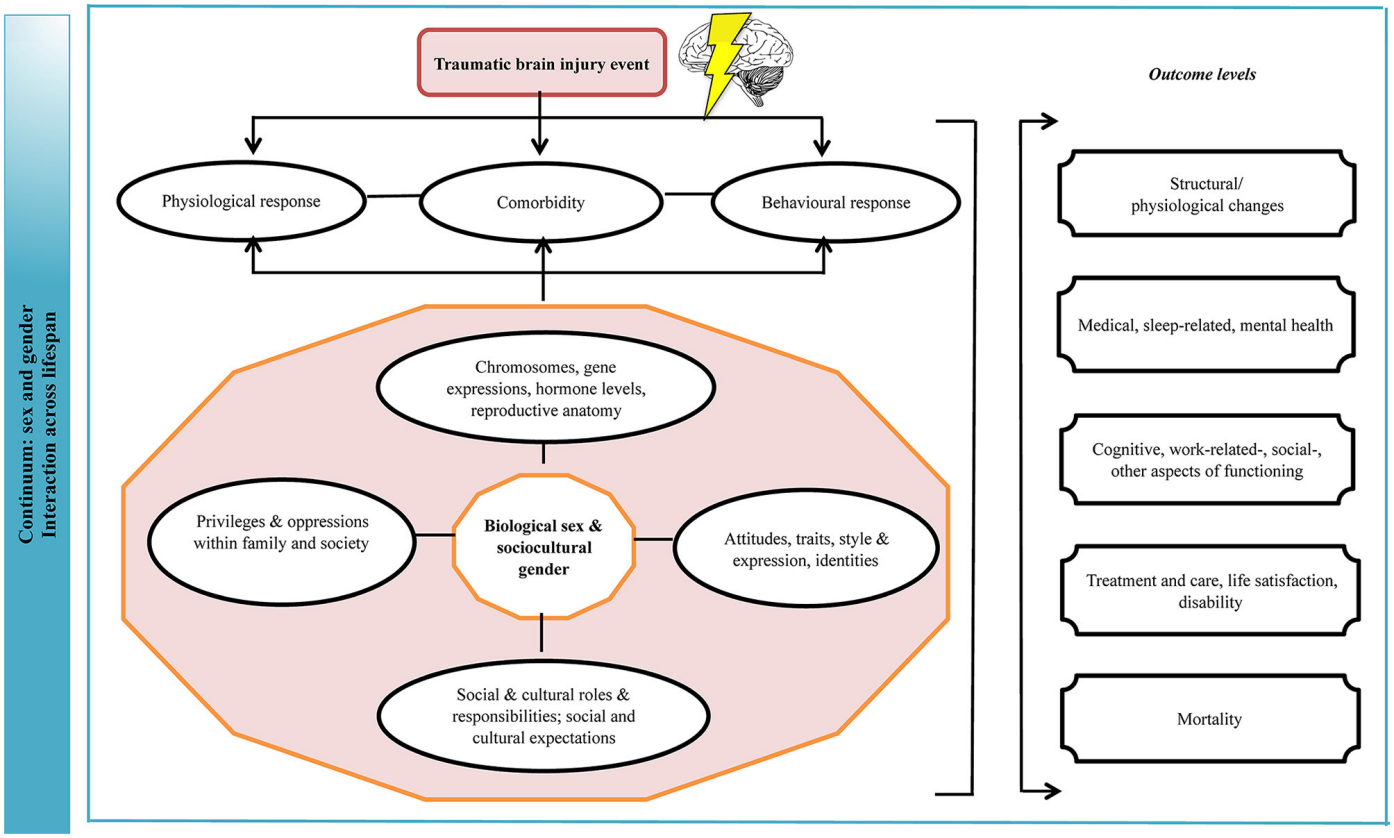
Logic model of sex and gender effects on outcomes of TBI. The implications are that biological and behavioral vulnerability to injury and gender disparities in norms and role expectations make certain groups of male and female persons be prone to a more favorable or adverse outcome due to their unique interactions between biological, behavioral, sociocultural elements preceding, at the time of, and following the injury. TBI, traumatic brain injury. This figure was modified from a previously published framework (22).

The field of TBI should view the results of this evidence synthesis as part of a still evolving body of work on the intersection of sex/gender in the health context (Figure 7). The evidence on the topic has grown over time, as attention to gender equality and social equity in different societies has increased and more research has been conducted. The data to date highlights theoretical and practical dilemmas that come with consideration of sex/gender in TBI research; and while with the current findings we cannot say definitively that there are risk and protective differences on the basis of sex/gender, the findings are compelling and support the merit of the topic. It is apparent that a healthcare system constructed on the principle of being “fair” by treating each individual the same, and thus ignoring gender norms, roles, and relations, precludes patient-centered care. Structured research centered on the topic of sex and gender and the transformative processes brought about by injury, measuring all relevant parameters and incorporating patient values, can clarify next steps for research and practice (131, 132).

## Data Availability Statement

The datasets presented in this study can be found in online repositories. The names of the repository/repositories and accession number(s) can be found in the article/Supplementary Material.

## Author Contributions

TM and AC contributed to the conception of the study. TM developed the idea, registered the review PROSPERO, designed and published the protocol, and developed study screening criteria and quality assessment criteria. TM, SM, and NP executed the study according to the protocol: NP and SM screened all abstracts, performed study quality assessment, and abstracted the data. TM guided the process. TM doubled checked all extracted data. TM, SM, and NP performed quantitative and qualitative data analyses. SM developed concept and performed visual presentation of the results. TM wrote the first draft of the review, which was reviewed by SM, NP, and AC. All authors contributed to the article and approved the submitted version.

## Funding

This work was supported by a research grant from the Canadian Institutes for Health Research Grant, Institute for Gender and Health (#CGW-126580). AC received grants from the Canadian Institutes of Health Research (CIHR) Chairs in Gender, Work and Health [grant no. CGW-126580] and Tier 1 Canada Research Chair in traumatic brain injury in underserved populations. TM was supported by the Alzheimer's Association Grant [AARF-16-442937]. The funders had no role in study design, data collection, decision to publish, or preparation of the manuscript.

## Conflict of Interest

The authors declare that the research was conducted in the absence of any commercial or financial relationships that could be construed as a potential conflict of interest.

## Publisher's Note

All claims expressed in this article are solely those of the authors and do not necessarily represent those of their affiliated organizations, or those of the publisher, the editors and the reviewers. Any product that may be evaluated in this article, or claim that may be made by its manufacturer, is not guaranteed or endorsed by the publisher.
