# Supplementary material for: Systematic Review of Sex and Gender Effects in Traumatic Brain Injury: Equity in Clinical and Functional Outcomes
Source: Front Neurol. 2021 Sep 10;12:678971. doi: 10.3389/fneur.2021.678971 (PMC8461184; doi:10.3389/fneur.2021.678971)
Supplement: Supplementary file 1 [file Data_Sheet_1.PDF]

Supplementary Figures and Tables

Supplementary Table 1: Search Strategy and Supporting Documentation 2

Supplementary Table 2: Excluded studies and reason for study exclusion. 12

Supplementary Table 3 (a) Quality assessment of observational studies. 20

Supplementary Table 3 (b) Quality assessment of case-control studies. 23

Supplementary Table 3 (c) Quality assessment of qualitative and case-series studies. 24

Supplementary Table 4: Summary of study characteristics, including details on study sample, design, methods and results on associative values of sex/gender, age and injury severity variables on clinical and non-clinical outcomes. 24

Supplementary Table 5: Summary of study characteristics, samples, and results pertaining to sex/gender and outcomes. 72

Supplementary Figure 1: Effect sizes of factors/variance explained by factors investigated in relation to post-TBI outcomes in multivariate regression analyses. 87

.... 88

Supplementary Table 1: Search Strategy and Supporting Documentation

SEARCH DETAILS

Searches conducted in Cochrane Central Register of Controlled Trials (CENTRAL), CINAHL, Embase, Ovid MEDLINE(R) (including Epub Ahead of Print, In-Process & Other Non-Indexed Citations, Ovid MEDLINE(R) Daily), PsycINFO, and Web of Science.

Search strategies included the use of textwords and subject headings (e.g. MeSH, Emtree) related to (1) traumatic brain injury and (2) sex or (3) gender. All searches were conducted from inception of the database to February 2018 and were subsequently updated in September 23 2019. Searches were limited to human studies, and English language papers when possible. Case reports were excluded when possible.

The following steps were conducted to establish the updated results to screen:

- Each database search was re-run (with minor edits if required)
- All results were exported into Endnote, and compared to the original search results form 2018. Duplicates were removed for each database (e.g. the original Embase results were compared to the updated Embase results, and duplicate records were removed to only keep the "new" records) – these results are reported below under "UPDATED SEARCH PRE-DUPLICATE REMOVAL" section.
- The remaining "new" results were then screened for duplicates internally, and against the original results as a whole. These results are listed below, under the "UPDATED NEW RESULTS POST-DUPLICATE REMOVAL" section.

UPDATED SEARCH PRE-DUPLICATE REMOVAL

TOTAL Results: 13010 (3578 NEW)

Cochrane Central Register of Controlled Trials (CENTRAL): 3425 (1924 NEW)
CINAHL: 850 (312 NEW)
Embase: 3095 (439 NEW)
Ovid MEDLINE(R): 2551 (385 NEW)
PsycINFO: 643 (77 NEW)
Web of Science: 2446 (441 NEW)

UPDATED NEW RESULTS POST-DUPLICATE REMOVAL

TOTAL Results: 2913

Cochrane Central Register of Controlled Trials (CENTRAL): 1898
CINAHL: 211
Embase: 375

Ovid MEDLINE(R): 193
PsycINFO: 27
Web of Science: 209

SEARCH STRATEGIES

Ovid MEDLINE(R) Epub Ahead of Print, In-Process & Other Non-Indexed Citations, Ovid MEDLINE(R) Daily and Ovid MEDLINE(R) <1946 to September 18, 2019>

Search history sorted by search number ascending			
**#**	**Searches**	**Results**	**Type**
1	Brain Injuries/	51277	Advanced
2	exp Brain Injuries, Traumatic/	12188	Advanced
3	exp Brain Concussion/	8145	Advanced
4	Craniocerebral Trauma/	21788	Advanced
5	tbi*2.tw,kf.	25241	Advanced
6	mtbi*2.tw,kf.	2613	Advanced
7	wrTBI*2.tw,kf.	13	Advanced
8	concuss*.tw,kf.	8744	Advanced
9	postconcuss*.tw,kf.	1296	Advanced
10	((head* or cerebr* or crani* or skull* or intracran*) adj2 (injur* or trauma* or damag* or wound* or swell* or oedema* or edema* or fracture* or contusion* or pressur*)).tw,kf.	84261	Advanced
11	((brain* or cerebr* or intracerebr* or crani* or intracran* or head* or subdural* or epidural* or extradural*) adj (haematoma* or hematoma* or hemorrhag* or haemorrhag* or bleed*)).tw,kf.	49922	Advanced
12	or/1-11	189121	Advanced
13	Sex Characteristics/	52303	Advanced
14	exp sex distribution/	62411	Advanced
15	exp Gender Identity/	18934	Advanced
16	(sex adj2 (differenc* or charact*)).tw,kf.	45043	Advanced
17	sex-related.tw,kf.	6154	Advanced
18	sex-based.tw,kf.	1458	Advanced
19	(sexes adj2 (differenc* or charact*)).tw,kf.	1880	Advanced
20	(gender adj2 (differenc* or charact*)).tw,kf.	38954	Advanced
21	gender-related.tw,kf.	4751	Advanced
22	gender-based.tw,kf.	2986	Advanced
23	(male? or men or man or masculin*).ti,kf.	286095	Advanced

24	(female? or women or woman or feminin*).ti,kf.	379569	Advanced
25	sex.ti,kf.	108081	Advanced
26	sexes.ti,kf.	1436	Advanced
27	gender*.ti,kf.	50705	Advanced
28	or/13-27	873700	Advanced
29	12 and 28	3465	Advanced
30	29 not (exp animals/ not exp humans/)	3226	Advanced
31	case reports/	2045653	Advanced
32	30 not 31	2789	Advanced
33	limit 32 to english language	2551	Advanced

Cochrane Central Register of Controlled Trials <2014 to Present>

Search history sorted by search number ascending			
**#**	**Searches**	**Results**	**Type**
1	Brain Injuries/	1613	Advanced
2	exp Brain Injuries, Traumatic/	1860	Advanced
3	exp Brain Concussion/	279	Advanced
4	Craniocerebral Trauma/	296	Advanced
5	tbi*2.tw,kw.	2784	Advanced
6	mtbi*2.tw,kw.	311	Advanced
7	wrTBI*2.tw,kw.	0	Advanced
8	concuss*.tw,kw.	560	Advanced
9	postconcuss*.tw,kw.	191	Advanced
10	((head* or cerebr* or crani* or skull* or intracran*) adj2 (injur* or trauma* or damag* or wound* or swell* or oedema* or edema* or fracture* or contusion* or pressur*)).tw,kw.	5765	Advanced
11	((brain* or cerebr* or intracerebr* or crani* or intracran* or head* or subdural* or epidural* or extradural*) adj (haematoma* or hematoma* or hemorrhag* or haemorrhag* or bleed*)).tw,kw.	6907	Advanced
12	or/1-11	15627	Advanced
13	Sex Characteristics/	1223	Advanced
14	exp sex distribution/	765	Advanced
15	exp Gender Identity/	255	Advanced
16	(sex adj2 (differenc* or charact*)).tw,kw.	5723	Advanced
17	sex-related.tw,kw.	263	Advanced
18	sex-based.tw,kw.	95	Advanced
19	(sexes adj2 (differenc* or charact*)).tw,kw.	157	Advanced

20	(gender adj2 (differenc* or charact*)).tw,kw.	3329	Advanced
21	gender-related.tw,kw.	262	Advanced
22	gender-based.tw,kw.	189	Advanced
23	(male? or men or man or masculin*).ti,kw.	228112	Advanced
24	(female? or women or woman or feminin*).ti,kw.	280614	Advanced
25	sex.ti,kw.	7565	Advanced
26	sexes.ti,kw.	19	Advanced
27	gender*.ti,kw.	11250	Advanced
28	or/13-27	337495	Advanced
29	12 and 28	3948	Advanced
30	29 not (exp animals/ not exp humans/)	3948	Advanced
31	case reports/	0	Advanced
32	30 not 31	3948	Advanced
33	limit 32 to english language	3711	Advanced
34	limit 33 to conference	286	Advanced
35	33 not 34	3425	Advanced

Embase <1974 to 2019 September 19>

Search history sorted by search number ascending			
**#**	**Searches**	**Results**	**Type**
1	exp traumatic brain injury/	46203	Advanced
2	brain injury/	86777	Advanced
3	exp concussion/	10869	Advanced
4	head injury/	44984	Advanced
5	tbi*2.tw,kw.	42494	Advanced
6	mtbi*2.tw,kw.	4505	Advanced
7	wrTBI*2.tw,kw.	17	Advanced
8	concuss*.tw,kw.	11735	Advanced
9	postconcuss*.tw,kw.	1637	Advanced
10	((head* or cerebr* or crani* or skull* or intracran*) adj2 (injur* or trauma* or damag* or wound* or swell* or oedema* or edema* or fracture* or contusion* or pressur*)).tw,kw.	106366	Advanced
11	((brain* or cerebr* or intracerebr* or crani* or intracran* or head* or subdural* or epidural* or extradural*) adj (haematoma* or hematoma* or hemorrhag* or haemorrhag* or bleed*)).tw,kw.	71026	Advanced
12	or/1-11	301995	Advanced
13	"gender and sex"/	1014	Advanced

14	gender identity/	16045	Advanced
15	(sex adj2 (differenc* or charact*)).tw,kw.	57141	Advanced
16	sex-related.tw,kw.	7545	Advanced
17	sex-based.tw,kw.	1940	Advanced
18	(sexes adj2 (differenc* or charact*)).tw,kw.	2425	Advanced
19	(gender adj2 (differenc* or charact*)).tw,kw.	57828	Advanced
20	gender-related.tw,kw.	6440	Advanced
21	gender-based.tw,kw.	3879	Advanced
22	(male? or men or man or masculin*).ti,kw.	307752	Advanced
23	(female? or women or woman or feminin*).ti,kw.	452386	Advanced
24	sex.ti,kw.	118101	Advanced
25	sexes.ti,kw.	1370	Advanced
26	gender*.ti,kw.	74437	Advanced
27	or/13-26	926480	Advanced
28	12 and 27	4734	Advanced
29	28 not ((exp animals/ or exp animal experimentation/ or nonhuman/) not exp human/)	3822	Advanced
30	limit 29 to english language	3530	Advanced
31	case study/	64526	Advanced
32	30 not 31	3505	Advanced
33	limit 32 to medline	410	Advanced
34	32 not 33	3095	Advanced

PsycINFO <1806 to September Week 2 2019>

Search history sorted by search number ascending			
**#**	**Searches**	**Results**	**Type**
1	exp traumatic brain injury/	18584	Advanced
2	head injuries/	4419	Advanced
3	tbi*2.tw.	10530	Advanced
4	mtbi*2.tw.	1775	Advanced
5	wrTBI*2.tw.	8	Advanced
6	concuss*.tw.	3207	Advanced
7	postconcuss*.tw.	840	Advanced
8	((head* or cerebr* or crani* or skull* or intracran*) adj2 (injur* or trauma* or damag* or wound* or swell* or oedema* or edema* or fracture* or contusion* or pressur*)).tw.	12246	Advanced

9	((brain* or cerebr* or intracerebr* or crani* or intracran* or head* or subdural* or epidural* or extradural*) adj (haematoma* or hematoma* or hemorrhag* or haemorrhag* or bleed*)).tw.	3424	Advanced
10	or/1-9	32573	Advanced
11	human sex differences/	109676	Advanced
12	exp gender identity/	36117	Advanced
13	(sex adj2 (differenc* or charact*)).tw.	36634	Advanced
14	sex-related.tw.	2076	Advanced
15	sex-based.tw.	525	Advanced
16	(sexes adj2 (differenc* or charact*)).tw.	450	Advanced
17	(gender adj2 (differenc* or charact*)).tw.	48456	Advanced
18	gender-related.tw.	3025	Advanced
19	gender-based.tw.	2845	Advanced
20	(male? or men or man or masculin*).ti.	80102	Advanced
21	(female? or women or woman or feminin*).ti.	114299	Advanced
22	sex.ti.	46916	Advanced
23	sexes.ti.	514	Advanced
24	gender*.ti.	50778	Advanced
25	or/11-24	347022	Advanced
26	10 and 25	719	Advanced
27	limit 26 to ("0200 book" or "0240 authored book" or "0280 edited book" or "0300 encyclopedia")	38	Advanced
28	exp case report/	22922	Advanced
29	26 not (27 or 28)	675	Advanced
30	limit 29 to english language	643	Advanced

CINAHL Search Strategy

#	Query	Limiters/Expanders	Last Run Via	Results
S28	S23 NOT S27	Search modes - Boolean/Phrase	Interface - EBSCOhost Research Databases<br>Search Screen - Advanced Search<br>Database - CINAHL with Full Text	850
S27	S24 OR S25 OR S26	Search modes - Boolean/Phrase	Interface - EBSCOhost Research Databases	384,980

			Search Screen - Advanced Search<br>Database - CINAHL with Full Text	
S26	(MH "Case Studies")	Search modes - Boolean/Phrase	Interface - EBSCOhost Research Databases<br>Search Screen - Advanced Search<br>Database - CINAHL with Full Text	22,013
S25	TI((case n1 (study or report))	Search modes - Boolean/Phrase	Interface - EBSCOhost Research Databases<br>Search Screen - Advanced Search<br>Database - CINAHL with Full Text	70,060
S24	PT case study	Search modes - Boolean/Phrase	Interface - EBSCOhost Research Databases<br>Search Screen - Advanced Search<br>Database - CINAHL with Full Text	341,976
S23	S11 AND S22	Limiters - English Language<br>Search modes - Boolean/Phrase	Interface - EBSCOhost Research Databases<br>Search Screen - Advanced Search<br>Database - CINAHL with Full Text	1,017
S22	S12 OR S13 OR S14 OR S15 OR S16 OR S17 OR S18 OR S19 OR S20 OR S21	Search modes - Boolean/Phrase	Interface - EBSCOhost Research Databases<br>Search Screen - Advanced Search<br>Database - CINAHL with Full Text	237,092
S21	TI sex or sexes or gender*	Search modes - Boolean/Phrase	Interface - EBSCOhost Research Databases<br>Search Screen - Advanced Search<br>Database - CINAHL with Full Text	44,146
S20	TI (female# or women or woman or feminin*)	Search modes - Boolean/Phrase	Interface - EBSCOhost Research Databases<br>Search Screen - Advanced Search<br>Database - CINAHL with Full Text	137,449
S19	TI (male# or men or man or masculin*)	Search modes - Boolean/Phrase	Interface - EBSCOhost Research Databases<br>Search Screen - Advanced Search<br>Database - CINAHL with Full Text	60,168
S18	gender-related	Search modes - Boolean/Phrase	Interface - EBSCOhost Research Databases	1,174

			Search Screen - Advanced Search<br>Database - CINAHL with Full Text	
S17	(gender n2 (differenc* or charact*))	Search modes - Boolean/Phrase	Interface - EBSCOhost Research Databases<br>Search Screen - Advanced Search<br>Database - CINAHL with Full Text	16,110
S16	(sexes n2 (differenc* or charact*))	Search modes - Boolean/Phrase	Interface - EBSCOhost Research Databases<br>Search Screen - Advanced Search<br>Database - CINAHL with Full Text	9,942
S15	sex-based	Search modes - Boolean/Phrase	Interface - EBSCOhost Research Databases<br>Search Screen - Advanced Search<br>Database - CINAHL with Full Text	463
S14	sex-related	Search modes - Boolean/Phrase	Interface - EBSCOhost Research Databases<br>Search Screen - Advanced Search<br>Database - CINAHL with Full Text	1,073
S13	(sex n2 (differenc* or charact*))	Search modes - Boolean/Phrase	Interface - EBSCOhost Research Databases<br>Search Screen - Advanced Search<br>Database - CINAHL with Full Text	9,942
S12	(MH "Gender Identity+")	Search modes - Boolean/Phrase	Interface - EBSCOhost Research Databases<br>Search Screen - Advanced Search<br>Database - CINAHL with Full Text	6,391
S11	S1 OR S2 OR S3 OR S4 OR S5 OR S6 OR S7 OR S8 OR S9 OR S10	Search modes - Boolean/Phrase	Interface - EBSCOhost Research Databases<br>Search Screen - Advanced Search<br>Database - CINAHL with Full Text	59,447
S10	((brain* or cerebr* or intracerebr* or crani* or intracran* or head* or subdural* or epidural* or extradural*) n1 (haematoma* or hematoma* or hemorrhag* or haemorrhag* or bleed*))	Search modes - Boolean/Phrase	Interface - EBSCOhost Research Databases<br>Search Screen - Advanced Search<br>Database - CINAHL with Full Text	15,121

S9	((head* or cerebr* or crani* or skull* or intracran*) n2 (injur* or trauma* or damag* or wound* or swell* or oedema* or edema* or fracture* or contusion* or pressur*))	Search modes - Boolean/Phrase	Interface - EBSCOhost Research Databases<br>Search Screen - Advanced Search<br>Database - CINAHL with Full Text	23,099
S8	postconcuss*	Search modes - Boolean/Phrase	Interface - EBSCOhost Research Databases<br>Search Screen - Advanced Search<br>Database - CINAHL with Full Text	1,033
S7	concuss*	Search modes - Boolean/Phrase	Interface - EBSCOhost Research Databases<br>Search Screen - Advanced Search<br>Database - CINAHL with Full Text	5,207
S6	wrTBI##	Search modes - Boolean/Phrase	Interface - EBSCOhost Research Databases<br>Search Screen - Advanced Search<br>Database - CINAHL with Full Text	8
S5	mTBI##	Search modes - Boolean/Phrase	Interface - EBSCOhost Research Databases<br>Search Screen - Advanced Search<br>Database - CINAHL with Full Text	934
S4	TBI##	Search modes - Boolean/Phrase	Interface - EBSCOhost Research Databases<br>Search Screen - Advanced Search<br>Database - CINAHL with Full Text	7,526
S3	(MH "Head Injuries")	Search modes - Boolean/Phrase	Interface - EBSCOhost Research Databases<br>Search Screen - Advanced Search<br>Database - CINAHL with Full Text	6,522
S2	(MH "Brain Concussion+")	Search modes - Boolean/Phrase	Interface - EBSCOhost Research Databases<br>Search Screen - Advanced Search<br>Database - CINAHL with Full Text	4,350
S1	(MH "Brain Injuries+")	Search modes - Boolean/Phrase	Interface - EBSCOhost Research Databases<br>Search Screen - Advanced Search<br>Database - CINAHL with Full Text	26,008

Web of Science Search Strategy

Set	Results	Search Strategy
# 17	2,446	#15 AND #4<br>**Refined by: LANGUAGES:** (ENGLISH)<br>*Indexes=SCI-EXPANDED, SSCI, A&HCI, CPCI-S, CPCI-SSH, BKCI-S, BKCI-SSH, ESCI Timespan=All years*
# 16	2,524	#15 AND #4<br>*Indexes=SCI-EXPANDED, SSCI, A&HCI, CPCI-S, CPCI-SSH, BKCI-S, BKCI-SSH, ESCI Timespan=All years*
# 15	1,366,275	#14 OR #13 OR #12 OR #11 OR #10 OR #9 OR #8 OR #7 OR #6 OR #5<br>*Indexes=SCI-EXPANDED, SSCI, A&HCI, CPCI-S, CPCI-SSH, BKCI-S, BKCI-SSH, ESCI Timespan=All years*
# 14	160,852	TI=((sex or sexes))<br>*Indexes=SCI-EXPANDED, SSCI, A&HCI, CPCI-S, CPCI-SSH, BKCI-S, BKCI-SSH, ESCI Timespan=All years*
# 13	656,247	TI=((female* OR women OR woman OR feminin*))<br>*Indexes=SCI-EXPANDED, SSCI, A&HCI, CPCI-S, CPCI-SSH, BKCI-S, BKCI-SSH, ESCI Timespan=All years*
# 12	509,179	TI=((male* OR men OR man OR masculin*))<br>*Indexes=SCI-EXPANDED, SSCI, A&HCI, CPCI-S, CPCI-SSH, BKCI-S, BKCI-SSH, ESCI Timespan=All years*
# 11	6,390	**TOPIC:** ((gender-based))<br>*Indexes=SCI-EXPANDED, SSCI, A&HCI, CPCI-S, CPCI-SSH, BKCI-S, BKCI-SSH, ESCI Timespan=All years*
# 10	7,232	**TOPIC:** ((gender-related))<br>*Indexes=SCI-EXPANDED, SSCI, A&HCI, CPCI-S, CPCI-SSH, BKCI-S, BKCI-SSH, ESCI Timespan=All years*
# 9	104,127	**TOPIC:** ((gender NEAR/2 (differenc* OR charact*)))<br>*Indexes=SCI-EXPANDED, SSCI, A&HCI, CPCI-S, CPCI-SSH, BKCI-S, BKCI-SSH, ESCI Timespan=All years*
# 8	90,428	**TOPIC:** ((sexes NEAR/2 (differenc* OR charact*)))<br>*Indexes=SCI-EXPANDED, SSCI, A&HCI, CPCI-S, CPCI-SSH, BKCI-S, BKCI-SSH, ESCI Timespan=All years*
# 7	2,175	**TOPIC:** ((sex-based))<br>*Indexes=SCI-EXPANDED, SSCI, A&HCI, CPCI-S, CPCI-SSH, BKCI-S, BKCI-SSH, ESCI Timespan=All years*
# 6	7,364	**TOPIC:** ((sex-related))<br>*Indexes=SCI-EXPANDED, SSCI, A&HCI, CPCI-S, CPCI-SSH, BKCI-S, BKCI-SSH, ESCI Timespan=All years*
# 5	90,428	**TOPIC:** ((sex NEAR/2 (differenc* OR charact*)))<br>*Indexes=SCI-EXPANDED, SSCI, A&HCI, CPCI-S, CPCI-SSH, BKCI-S, BKCI-SSH, ESCI Timespan=All years*
# 4	173,882	#3 OR #2 OR #1<br>*Indexes=SCI-EXPANDED, SSCI, A&HCI, CPCI-S, CPCI-SSH, BKCI-S, BKCI-SSH, ESCI Timespan=All years*
# 3	54,477	**TOPIC:** (((brain* or cerebr* or intracerebr* or crani* or intracran* or head* or subdural* or epidural* or extradural*) NEAR/1 (haematoma* or hematoma* or hemorrhag* or haemorrhag* or bleed*)))

		Indexes=SCI-EXPANDED, SSCI, A&HCI, CPCI-S, CPCI-SSH, BKCI-S, BKCI-SSH, ESCI Timespan=All years
# 2	105,918	**TOPIC:** (((head* or cerebr* or crani* or skull* or intracran*) NEAR/2 (injur* or trauma* or damag* or wound* or swell* or oedema* or edema* or fracture* or contusion* or pressur*)))<br>*Indexes=SCI-EXPANDED, SSCI, A&HCI, CPCI-S, CPCI-SSH, BKCI-S, BKCI-SSH, ESCI Timespan=All years*
# 1	32,625	**TOPIC:** ((tbi* OR mTBI* OR wrTBI* OR concuss OR postconcuss))<br>*Indexes=SCI-EXPANDED, SSCI, A&HCI, CPCI-S, CPCI-SSH, BKCI-S, BKCI-SSH, ESCI Timespan=All years*

Supplementary Table 2: Excluded studies and reason for study exclusion.

Title	Author (Year)	Reason for exclusion
1. Anxiety Sensitivity Mediates Gender Differences in Post-Concussive Symptoms in a Clinical Sample	Albanese et al. (2017)	No multivariate analysis performed
2. The Impact of Neurobehavioral Impairment on Family Functioning and the Psychological Well-Being of Male Versus Female Caregivers of Relatives With Severe Traumatic Brain Injury: Multigroup Analysis	Anderson et al. (2013)	Age not included in multivariate model
3. Exploring variables associated with rehabilitation length of stay in brain injuries patients	Avesani et al. (2012)	No multivariate analysis performed
4. Disability and health-related quality-of-life 4 years after a severe traumatic brain injury: A structural equation modelling analysis	Azouvi et al. (2016)	No multivariate analysis performed
5. Themes within Songs Written by People with Traumatic Brain Injury: Gender Differences	Baker et al. (2005)	Large age range which incorporates adolescent patients
6. Sex Differences in Depressive Symptoms and Their Correlates After Mild-to-Moderate Traumatic Brain Injury	Bay et al. (2009)	No multivariate analysis performed
7. Sex Differences in Outcome after Mild Traumatic Brain Injury	Bazarian et al. (2010)	Large age range which incorporates adolescent patients
8. Mothers Report More Child-Rearing Disagreements Following Early Brain Injury Than Do Fathers	Bendikas et al. (2011)	Pediatric population
9. Gender and age predict outcomes of cognitive, balance and vision testing in a multidisciplinary concussion center	Benedict et al. (2015)	No multivariate analysis performed
10. Emergency care of traumatic brain injuries in Pakistan: a multicenter study	Bhatti et al. (2015)	Severity not reported on or included in multivariate analysis
11. Effect of sex and age on traumatic brain injury: a geographical comparative study	Biswas et al. (2017)	No multivariate analysis performed

12. Gender differences in a sample of vocational rehabilitation clients with TBI	Bounds et al. (2003)	Age not included in multivariate analysis
13. Clinical Course Score (CCS): A New Clinical Score to Evaluate Efficacy of Neurotrauma Treatment in Traumatic Brain Injury and Subarachnoid Hemorrhage	Brandner et al. (2014)	No multivariate analysis performed
14. Sex Differences in Emotional Status of Traumatically Brain-Injured Patients	Burton et al. (1988)	No multivariate analysis performed
15. Sex-specific predictors of inpatient rehabilitation outcomes after traumatic brain injury	Chan et al. (2016)	Severity not included in multivariate analysis
16. Examining the epidemiology of work-related traumatic brain injury through a sex/gender lens: analysis of workers' compensation claims in Victoria, Australia	Chang et al. (2014)	No multivariate analysis performed
17. Trends in traumatic brain injury mortality in China, 2006-2013: A population-based longitudinal study	Cheng et al. (2017)	Severity not reported on or included in multivariate analysis
18. Trajectories of sleep changes during the acute phase of traumatic brain injury: A 7-day actigraphy study	Chiu et al. (2013)	Age not included in final hierarchical linear model of 24-hour total sleep time subgroup analysis of mild TBI
19. Does Sexual Dimorphism Influence Outcome of Traumatic Brain Injury Patients? The Answer Is No!	Coimbra et al. (2003)	No multivariate analysis performed
20. Predictors of Postacute Mortality Following Traumatic Brain Injury in a Seriously Injured Population	Colantonio et al. (2008)	Sex not reported in multivariate analysis
21. Gender differences in self reported long term outcomes following moderate to severe traumatic brain injury	Colantonio et al. (2010)	No multivariate analysis performed
22. Traumatic Brain Injury and Early Life Experiences Among Men and Women in a Prison Population	Colantonio et al. (2014)	No multivariate analysis performed
23. A Rapid Screen of the Severity of Mild Traumatic Brain Injury	Comerford et al. (2002)	No multivariate analysis performed
24. Sex and Age Differences in Depression and Baseline Sport-Related Concussion Neurocognitive Performance and Symptoms	Covassin et al. (2012)	Patients did not have a brain injury
25. Sex-Based Differences in Perceived Pragmatic Communication Ability of Adults With Traumatic Brain Injury	Despins et al. (2016)	No multivariate analysis performed
26. Newfound sex differences in axonal structure underlie differential outcomes from in vitro traumatic axonal injury	Dolle et al. (2018)	In vitro study
27. Olfactory Dysfunction in Patients With Head Trauma	Doty et al. (1997)	No multivariate analysis performed

28. Fatigue after traumatic brain injury: Association with neuroendocrine, sleep, depression and other factors	Englander et al. (2010)	Age not included in multivariate analysis
29. Sex Differences in White Matter Abnormalities after Mild Traumatic Brain Injury: Localization and Correlation with Outcome	Fakhran et al. (2014)	Large age range which incorporates adolescent patients
30. Prehospital management of traumatic brain injury patients- a gender perspective	Falk et al. (2015)	Selective reporting of data and fail to include detailed results or table for regression model
31. Sex-related differences in patients with severe head injury: greater susceptibility to brain swelling in female patients 50 years of age and younger	Farin et al. (2003)	No multivariate analysis performed
32. Co-morbid traumatic brain injury and substance use disorder: Childhood predictors and adult correlates	Felde et al. (2005)	Severity not included in multivariate analysis
33. Motor Recover During the Acute Period of Craniocerebral Trauma Using Kinetotherapy	Franckeviciute et al. (2008)	No multivariate analysis performed
34. Health & Economic Burden of Traumatic Brain Injury in the Emergency Department	Fu et al. (2016)	No multivariate analysis performed
35. Predictors of rapid spontaneous resolution of acute subdural hematoma	Fujimoto et al. (2014)	Age not included in multivariate analysis
36. American Community Survey: Earnings and employment for persons with traumatic brain injury	Gamboa et al. (2006)	No multivariate analysis performed
37. Variations in admissions to hospital for head injury and assault to the head Part 1: age and gender	Gilthorpe et al. (1999)	No multivariate analysis performed
38. Sexual Functioning and the Effect of Fatigue in Traumatic Brain Injury	Goldin et al. (2014)	Severity not included in multivariate analysis
39. Traumatic Brain Injury and Suicidal Ideation Among U.S. Operation Enduring Freedom and Operation Iraqi Freedom Veterans	Gradus et al. (2015)	Age and severity not included in multivariate analysis
40. Influence of Sex and Age on Inpatient Rehabilitation Outcomes Among Older Adults With Traumatic Brain Injury	Graham et al. (2010)	Severity not reported on or included in multivariate analysis
41. Female TBI patients recover better than males	Groswasser et al. (1998)	Large age range which incorporates pediatric and adolescent patients
42. Interview of women whose husbands had head injury	Gosling et al. (1999)	Rearranged marriages: marital relationship after head injury
43. Sex differences in mortality after traumatic brain injury, Colorado 1994–1998	Gujral et al. (2006)	Severity not reported on or included in multivariate analysis

44. Rehabilitation Utilization following a Work- Related Traumatic Brain Injury: A Sex-Based Examination of Workers' Compensation Claims in Victoria, Australia	Guerriero et al. (2016)	Severity not reported on or included in multivariate analysis
45. Which factors influence the activity levels of individuals with traumatic brain injury when they are first discharged home from hospital?	Hamilton et al. (2015)	Sex, age, and injury severity not included in final regression model
46. Models of Mortality and Morbidity in Severe Traumatic Brain Injury: An Analysis of a Singapore Neurotrauma Database	Han et al. (2017)	Sex not included in multivariate analysis
47. Acute predictors for mortality after severe TBI in Spain: Gender differences and clinical data	Herrera-Melero et al. (2015)	Sex not included in multivariate analysis
48. Quality of Life for Individuals with Traumatic Brain Injury: The Influence of Attachment Security and Partner Support	Hess et al. (2016)	No multivariate analysis performed
49. Development of Posttraumatic Stress Disorder After Mild Traumatic Brain Injury	Hoffman et al. (2012)	Sex, age, and injury severity not included in multivariate analysis
50. Sex Differences in Working Memory after Mild Traumatic Brain Injury: A Functional MR Imaging Study	Hsu et al. (2015)	No multivariate analysis performed
51. Psychiatric Diagnoses and Neurobehavioral Symptom Severity Among OEF/OIF VA Patients with Deployment-Related Traumatic Brain Injury: A Gender Comparison	Iverson et al. (2011)	Age not included in multivariate analysis
52. Deployment-Related Traumatic Brain Injury Among Operation Enduring Freedom/Operation Iraqi Freedom Veterans: Associations with Mental and Physical Health by Gender	Iverson et al. (2013)	Severity not reported on or included in multivariate analysis
53. Mild Traumatic Brain Injury, PTSD, and Psychosocial Functioning Among Male and Female U.S. OEF/OIF Veterans	Jackson et al. (2016)	No multivariate analysis performed
54. The influence of sex and pre-traumatic headache on the incidence and severity of headache after head injury	Jenson et al. (1990)	No multivariate analysis performed
55. Memories following physical trauma in patients treated in the ICU: Does gender and head injury make a difference?	Johansson et al. (2008)	Age not included in multivariate analysis
56. Sensation Seeking and Risk Behaviors in Young Adults With and Without a History of Head Injury	O'Jile et al. (2004)	Unclear multivariate analysis-appears severity not included
57. Wives' coping flexibility, time since husbands' injury and the perceived burden of wives of men with traumatic brain injury	Katz et al. (2005)	No multivariate analysis performed
58. Gender Differences in Agitation After Traumatic Brain Injury	Kaydan et al. (2004)	No multivariate analysis performed
59. Depression in Men and Women One Year Following Traumatic Brain Injury (TBI): A TBI Model Systems Study	Lavoie et al. (2017)	No multivariate analysis performed

60. Affective symptoms in the chronic stage of<br>61. traumatic brain injury: a study of married couples	Lin et al. (1994)	No multivariate analysis performed
62. Public attitudes towards survivors of brain injury	Linden et al. (2010)	Adolescent population
63. Gender as a Moderator of Cognitive and Affective Outcome After Traumatic Brain Injury	Liossi et al. (2009)	Severity not included in final multivariate model<br>*Participants were matched based on severity however this match was not exact
64. The Negative Impact of Anemia in Outcome from Traumatic Brain Injury	Litofsky et al. (2016)	Sex not included in multivariate analysis
65. Neuropsychological Performance and Sleep<br>66. Disturbance Following Traumatic Brain Injury	Mahmood et al. (2004)	Age not included in multivariate analysis
67. Genetic Variation in the Vesicular Monoamine Transporter: Preliminary associations with Cognitive Outcomes after Severe Traumatic Brain Injury	Markos et al. (2017)	Sex not included in multivariate analysis
68. Risk factors for chronic subdural haematoma formation do not account for the established male bias	Marshman et al. (2015)	Not all cases of chronic subdural haematoma formation were the result of a traumatic etiology
69. Mortality in Severe Traumatic Brain Injury: A Multivariated Analysis of 748 Brazilian Patients From Florianopolis City	Martins et al. (2009)	Sex not included in multivariate analysis
70. Sex differences in orbitofrontal connectivity in male and female veterans with TBI	McGlade et al. (2015)	No multivariate analysis performed
71. The Prospective Course of Postconcussion Syndrome: The Role of Mild Traumatic Brain Injury	Meares et al. (2011)	Age not included in multivariate analysis
72. Variations in admission to hospital for head injury and assault to the head Part 2: ethnic group	Moles et al. (1999)	No multivariate analysis performed
73. Does gender influence cognitive outcome after<br>74. traumatic brain injury?	Moore et al. (2010)	Age not included in multivariate analysis
75. Differences according to Sex in Sociosexuality and Infidelity after Traumatic Brain Injury	Moreno et al. (2015)	No multivariate analysis performed
76. Prognostic Value of Demographic Characteristics in Traumatic Brain Injury: Results from the IMPACT Study	Mushkudiani et al. (2007)	Severity not included in multivariate analysis
77. Inflammation and Outcome in Traumatic Brain Injury: Does Gender Effect on Survival and Prognosis?	Naghibi et al. (2017)	No multivariate analysis performed

78. Can early clinical parameters predict post-traumatic pituitary dysfunction in severe traumatic brain injury?	Nemes et al. (2016)	No multivariate analysis performed
79. Head trauma and Parkinson's disease: results from an Italian case-control study	Nicoletti et al. (2017)	Severity not reported on or included in multivariate analysis
80. Is head injury a risk factor for schizophrenia?	Nielson et al. (2002)	Age not included in multivariate analysis
81. Gender differences in executive functions following traumatic brain injury	Niemeier et al. (2007)	Age not included in multivariate analysis
82. Gender Differences in Awareness and Outcomes<br>83. During Acute Traumatic Brain Injury Recovery	Niemeier et al. (2014)	No multivariate analysis performed
84. Influence of Gender on Occurrence of Chronic Subdural Hematoma; Is It an Effect of Cranial Asymmetry?	Oh et al. (2014)	No multivariate analysis performed
85. Apolipoprotein E polymorphism and gender difference in outcome after severe traumatic brain injury	Ost et al. (2008)	No multivariate analysis performed
86. Postoperative subdural hygroma and chronic subdural hematoma after unruptured aneurysm surgery: age, sex, and aneurysm location as independent risk factors	Park et al. (2016)	Severity not reported on or included in multivariate analysis
87. Sleep Quality and Reexperiencing Symptoms of PTSD Are Associated With Current Pain in U.S. OEF/OIF/OND Veterans With and Without mTBIs	Powell et al. (2015)	Cannot confirm that all participants have experienced a TBI
88. A retrospective cohort study of comorbidity trajectories associated with traumatic brain injury in veterans of the Iraq and Afghanistan wars	Pugh et al. (2016)	No multivariate analysis performed
89. Trajectories of Life Satisfaction in the First 5 Years Following Traumatic Brain Injury	Resch et al. (2009)	Severity not included in multivariate analysis
90. Prevalence and types of sleep disturbances acutely after traumatic brain injury	Rao et al.	Analysis did not account for TBI severity and age when reporting association
91. Years of Life Lost Because of Gunshot Injury to the Brain and Spinal Cord	Richmond et al. (2008)	No multivariate analysis performed
92. Sexual Behavior After Head Injury in Indian Men and Women	Sabhesan et al. (1989)	No multivariate analysis performed
93. Traumatic Brain Injury and Post-Deployment Binge Drinking among Male and Female Army Active Duty Service Members Returning from Operation Enduring Freedom/Operation Iraqi Freedom	Sayko Adams et al. (2017)	Severity and age not included in multivariate analysis
94. Gender- and age-related role changes following brain injury	Schmidt et al. (1995)	No multivariate analysis performed

95. A Comparison of Adult Outcomes for Males Compared to Females Following Pediatric Traumatic Brain Injury	Scott et al. (2015)	No multivariate analysis performed
96. The Effect of Insurance Status, Race, and Gender on ED Disposition of Persons With Traumatic Brain Injury	Selassie et al. (2004)	Large age range which incorporates pediatric and adolescent patients
97. Sex Differences in Injury Severity and Outcome Measures After Traumatic Brain Injury	Slewa-Younan et al. (2004)	No multivariate analysis performed
98. Do men and women differ in their course following traumatic brain injury? A preliminary prospective investigation of early outcome	Slewa-Younan et al. (2008)	No multivariate analysis performed
99. Predictors of mortality in patients with isolated severe traumatic brain injury	Strnad et al. (2017)	Age not included in multivariate analysis
100. Sex Differences In Symptoms, Disability and Life Satisfaction Three Years After Mild Traumatic Brain Injury: A Population based Cohort Study	Styrke et al. (2013)	Age not included in multivariate analysis
101. Gender differences in nonlinear motor performance following concussion	Studenka et al. (2017)	No multivariate analysis performed
102. Mood Disorders after Traumatic Brain Injury in Adolescents and Young Adults: A Nationwide Population-Based Cohort Study	Tsai et al. (2014)	Population includes adolescents and adults and severity not reported on or included in analysis
103. Suicidal Ideation Following Traumatic Brain Injury: Prevalence Rates and Correlates in Adults Living in the Community	Tsaousides et al. (2011)	No multivariate analysis performed
104. The Association Between Skull Bone Fractures and Outcomes in Patients With Severe Traumatic Brain Injury	Tseng et al. (2011)	Sex not included in multivariate analysis
105. Risk Factors for Chronic Subdural Hematoma after a Minor Head Injury in the Elderly: A Population-Based Study	Tseng et al. (2014)	Unclear if head injury was a result of trauma or other causes
106. Fatigue and its relationship with physical activity in adolescents and young adults with traumatic brain injury: a cross-sectional study	van Markus-Doornbosch et al. (2017)	Adolescent and adult population not separated with respect to analysis
107. Gender associations with cerebrospinal fluid glutamate and lactate/ pyruvate levels after severe traumatic brain injury	Wagner et al. (2005)	Age not included in multivariate analysis
108. Acute Serum Hormone Levels: Characterization and Prognosis after Severe Traumatic Brain Injury	Wagner et al. (2011)	No multivariate analysis performed
109. The association between BDNF Val66Met polymorphism and emotional symptoms after mild traumatic brain injury	Wang et al. (2018)	Age not included in multivariate analysis

110. Factors predictive of outcome in posttraumatic seizures	Wang et al. (2008)	Included all ages, cannot differentiate adults
111. Traumatic Brain Injury, PTSD, and Current Suicidal Ideation Among Iraq and Afghanistan U.S. Veterans	Wisco et al. (2014)	Severity not reported on or included in multivariate analysis
112. Risk factors indicating the need for cranial CT scans in elderly patients with head trauma: an Austrian trial and comparison with the Canadian CT Head Rule	Wolf et al. (2014)	Sex not included in final multivariate model
113. Factors associated with return to work in men and women with work-related traumatic brain injury	Xiong et al. (2016)	No multivariate analysis performed
114. A retrospective analysis of postoperative recurrence of septated chronic subdural haematoma: endoscopic surgery versus burr hole craniotomy	Yan et al. (2017)	No multivariate analysis performed
115. Protection From Traumatic Brain Injury in Hormonally Active Women vs Men of a Similar Age	Yeung et al. (2011)	Age not included in multivariate analysis
116. Functional outcome after head injury: Comparison of 12–45 year old male and female hormonally active patients	Yu et al. (2012)	Age and sex not reported in multivariate analysis
117. Risk factors for intracranial injury diagnosed by cranial CT in emergency department patients with mTBI	Zhang et al. (2017)	Sex not included in final multivariate model

Supplementary Table 3 (a) Quality assessment of observational studies.

**Study**	1	2	3	4	5	6	7	8	9	10	11	12	13	14	**Score**
Albrecht et al. (2017)	Yes	Yes	Yes	Yes	Yes	No	No	Yes	Yes	No	Yes	CD	Yes	Yes	+
Arellano-Orden et al. (2011)	Yes	Yes	Yes	Yes	No	No	No	US	Yes	Yes	Yes	CD	Yes	US	+
Berry et al. (2009)	Yes	Yes	Yes	Yes	No	No	No	Yes	Yes	No	Yes	CD	Yes	Yes	+
Boutin et al. (2017)	Yes	Yes	Yes	Yes	Yes	No	No	Yes	Yes	No	Yes	CD	Yes	Yes	+
Brown et al. (2012)	Yes	Yes	Yes	Yes	No	No	No	No	Yes	No	Yes	CD	Yes	Yes	+
Clond et al. (2011)	Yes	Yes	Yes	Yes	No	No	No	Yes	Yes	No	Yes	CD	Yes	Yes	+
Corrigan et al. (2007)	Yes	Yes	Yes	Yes	No	Yes	Yes	Yes	Yes	Yes	Yes	CD	Yes	Yes	+
Cote et al. (2013)	Yes	Yes	Yes	Yes	No	No	No	Yes	Yes	No	Yes	CD	Yes	Yes	+
Dahdah et al. (2016)	Yes	Yes	Yes	Yes	Yes	Yes	Yes	US	Yes	Yes	Yes	CD	Yes	Yes	+
Davis et al. (2006)	Yes	Yes	Yes	Yes	No	No	No	Yes	Yes	No	Yes	CD	Yes	Yes	+
Eramudugolla et al. (2014)	Yes	Yes	Yes	Yes	No	Yes	Yes	Yes	Yes	Yes	Yes	CD	Yes	Yes	+
Forslund et al. (2017)	Yes	Yes	Yes	Yes	No	Yes	Yes	Yes	Yes	Yes	Yes	CD	Yes	Yes	+
Fortuna et al. (2008)	Yes	Yes	Yes	Yes	No	No	No	Yes	Yes	No	Yes	CD	Yes	Yes	+
Gerhart et al. (2002)	Yes	Yes	Yes	Yes	Yes	Yes	No	Yes	Yes	No	Yes	CD	Yes	Yes	+
Glenn et al. (2001)	Yes	Yes	Yes	Yes	No	No	No	Yes	Yes	No	Yes	CD	Yes	Yes	+
Hoffman et al. (2012)	Yes	Yes	Yes	Yes	No	No	No	Yes	Yes	No	Yes	CD	Yes	Yes	+
Jung et al. (2014)	Yes	Yes	Yes	Yes	No	No	No	Yes	Yes	No	Yes	CD	Yes	Yes	+
Kirkness et al. (2004)	Yes	Yes	CD	Yes	No	Yes	Yes	Yes	Yes	No	Yes	CD	Yes	Yes	+
Kisat et al. (2012)	Yes	Yes	Yes	Yes	No	No	No	Yes	Yes	No	Yes	CD	Yes	Yes	+
Lee et al. (2014)	Yes	Yes	Yes	Yes	No	Yes	Yes	US	Yes	No	Yes	CD	Yes	Yes	+
Leitgeb et al. (2011)	Yes	Yes	Yes	Yes	Yes	Yes	Yes	Yes	Yes	No	Yes	CD	Yes	Yes	+
Li et al. (2018)	Yes	Yes	Yes	Yes	No	No	No	Yes	Yes	No	Yes	CD	Yes	Yes	+
Meares et al. (2008)	Yes	Yes	Yes	Yes	No	Yes	Yes	Yes	Yes	No	Yes	CD	Yes	Yes	+
Mellick et al. (2002)	Yes	Yes	Yes	Yes	Yes	Yes	Yes	Yes	Yes	No	Yes	CD	Yes	Yes	+
Miles et al. (2015)	Yes	Yes	Yes	Yes	No	No	No	Yes	Yes	No	Yes	CD	Yes	Yes	+
Mollayeva et al. (2015)	Yes	Yes	Yes	Yes	No	No	No	Yes	Yes	No	Yes	CD	Yes	Yes	+

Mollayeva et al. (2016a)	Yes	Yes	Yes	Yes	Yes	No	No	Yes	Yes	No	Yes	CD	Yes	Yes	+
Mollayeva et al. (2016b)	Yes	Yes	Yes	Yes	Yes	No	No	Yes	Yes	No	Yes	CD	Yes	Yes	+
Mollayeva et al. (2017a)	Yes	Yes	Yes	Yes	No	No	No	Yes	Yes	No	Yes	CD	Yes	Yes	+
Mollayeva et al. (2017b)	Yes	Yes	Yes	Yes	Yes	No	No	Yes	Yes	Yes	Yes	CD	Yes	Yes	+
Mollayeva et al. (2019)	Yes	Yes	Yes	Yes	Yes	Yes	Yes	Yes	Yes	No	Yes	CD	N/A	Yes	+
Myrga et al. (2016)	Yes	Yes	Yes	Yes	No	Yes	Yes	Yes	Yes	Yes	Yes	Yes	Yes	Yes	+
Ng et al. (2006)	Yes	Yes	Yes	Yes	No	Yes	Yes	Yes	Yes	No	Yes	CD	Yes	Yes	+
Ostberg et al. (2014)	Yes	Yes	Yes	Yes	Yes	No	No	Yes	Yes	No	Yes	CD	Yes	Yes	+
Ottochian et al. (2009)	Yes	Yes	Yes	Yes	No	No	No	Yes	Yes	No	Yes	CD	Yes	Yes	+
Ponsford et al. (2008)	Yes	Yes	Yes	Yes	CD	Yes	Yes	Yes	CD	Yes	Yes	No	Yes	Yes	+
Pogoda et al. (2012)	Yes	Yes	Yes	Yes	No	No	No	Yes	Yes	No	Yes	CD	Yes	Yes	+
Rao et al. (2008)	Yes	Yes	Yes	Yes	No	Yes	Yes	Yes	Yes	No	Yes	CD	Yes	Yes	+
Renner et al. (2012)	Yes	Yes	Yes	Yes	No	Yes	Yes	Yes	Yes	No	Yes	CD	Yes	Yes	+
Rush et al. (2016)	Yes	Yes	Yes	Yes	Yes	No	No	Yes	Yes	No	Yes	CD	Yes	Yes	+
Saban et al. (2011)	Yes	Yes	Yes	Yes	No	No	No	No	Yes	No	Yes	CD	Yes	Yes	+
Selassie et al. (2014)	Yes	Yes	Yes	Yes	No	No	No	Yes	Yes	No	Yes	CD	Yes	Yes	+
Shonberger et al. (2009)	Yes	Yes	Yes	Yes	No	No	No	Yes	Yes	No	Yes	CD	Yes	Yes	+
Sutton et al. (2019)	Yes	Yes	Yes	Yes	No	N/A	Yes	Yes	Yes	No	Yes	CD	N/A	Yes	+
Takada et al. (2016)	Yes	Yes	Yes	Yes	No	N/A	Yes	Yes	Yes	No	Yes	No	N/A	Yes	+
Tawil et al. (2008)	Yes	Yes	Yes	Yes	No	No	No	Yes	Yes	No	Yes	Yes	Yes	Yes	+
Tsushima et al. (2009)	Yes	Yes	Yes	Yes	CD	No	No	Yes	Yes	No	Yes	CD	Yes	Yes	+
van der Horn et al. (2013)	Yes	Yes	Yes	Yes	No	No	No	Yes	Yes	No	Yes	CD	Yes	Yes	+
Vikane et al. (2016)	Yes	Yes	Yes	Yes	No	Yes	Yes	Yes	Yes	No	Yes	CD	Yes	Yes	+
Wagner et al. (2004)	Yes	Yes	Yes	Yes	No	No	No	Yes	Yes	Yes	Yes	Yes	Yes	Yes	+
Wagner et al. (2007)	Yes	Yes	Yes	Yes	No	Yes	Yes	Yes	Yes	Yes	Yes	CD	Yes	Yes	+

Wang et al. (2008)	Yes	Yes	Yes	Yes	No	No	No	Yes	Yes	No	Yes	CD	Yes	Yes	+
Wang et al. (2018)	Yes	Yes	Yes	Yes	No	No	No	No	Yes	No	Yes	CD	Yes	Yes	+
Williamson et al. (2016)	Yes	Yes	Yes	Yes	No	Yes	Yes	Yes	Yes	No	Yes	CD	Yes	Yes	+

* CD, cannot determine; ɑ, has parent study; N/A, not applicable; NR, not reported.

Criteria:

1. Was the research question or objective in this paper clearly stated?
2. Was the study population clearly specified and defined?
3. Was the participation rate of eligible persons at least 50%?
4. Were all the subjects selected or recruited from the same or similar populations (including the same time period)? Were inclusion and exclusion criteria for being in the study prespecified and applied uniformly to all participants?
5. Was a sample size justification, power description, or variance and effect estimates provided?
6. For the analyses in this paper, were the exposure(s) of interest measured prior to the outcome(s) being measured?
7. Was the timeframe sufficient so that one could reasonably expect to see an association between exposure and outcome if it existed?
8. For exposures that can vary in amount or level, did the study examine different levels of the exposure as related to the outcome (e.g., categories of exposure, or exposure measured as continuous variable)?
9. Were the exposure measures (independent variables) clearly defined, valid, reliable, and implemented consistently across all study participants?
10. Was the exposure(s) assessed more than once over time?
11. Were the outcome measures (dependent variables) clearly defined, valid, reliable, and implemented consistently across all study participants?
12. Were the outcome assessors blinded to the exposure status of participants?
13. Was loss to follow-up after baseline 20% or less?
14. Were key potential confounding variables measured and adjusted statistically for their impact on the relationship between exposure(s) and outcome(s)?

QUIPS Six Potential Sources of Bias:

1. Study Design→ 1,10, 12
2. Study Participation→ 2,34
3. Study Attrition→ 13
4. Associated Factors→ 9
5. Outcome Measures/ Confounding Account→ 11,14
6. Analysis→5,6,7,8

Summary of Scoring: In addition to crude calculation of total number of biases present out of total number of possible, each study quality was summarized qualitatively into three groups: i) "++" when all or most of the quality were fulfilled (i.e. allowing one "CD" or "NR" based six

potential sources of bias (i.e. study participation, design and attrition, associated factors, outcome measurements, confounding variables, and analyses).); ii) "+" when half items of criteria were fulfilled; iii) "-" when less than half of items of criteria were fulfilled.

Supplementary Table 3 (b) Quality assessment of case-control study.

**Study**	1	2	3	4	5	6	7	8	9	10	11	12	**Score**
Harrison et al. (2006)	Yes	Yes	No	Yes	Yes	Yes	NA	CD	Yes	Yes	CD	Yes	**8/12 (+)**

*CD, cannot determine; NA, not applicable; NR, not reported

Criteria:

1. Was the research question or objective in this paper clearly stated and appropriate?
2. Was the study population clearly specified and defined?
3. Did the authors include a sample size justification?
4. Were controls selected or recruited from the same or similar population that gave rise to the cases (including the same timeframe)?
5. Were the definitions, inclusion and exclusion criteria, algorithms or processes used to identify or select cases and controls valid, reliable, and implemented consistently across all study participants?
6. Were the cases clearly defined and differentiated from controls?
7. If less than 100 percent of eligible cases and/or controls were selected for the study, were the cases and/or controls randomly selected from those eligible?
8. Was there use of concurrent controls?
9. Were the investigators able to confirm that the exposure/risk occurred prior to the development of the condition or event that defined a participant as a case?
10. Were the measures of exposure/risk clearly defined, valid, reliable, and implemented consistently (including the same time period) across all study participants?
11. Were the assessors of exposure/risk blinded to the case or control status of participants?
12. Were key potential confounding variables measured and adjusted statistically in the analyses? If matching was used, did the investigators account for matching during study analysis?

QUIPS Six Potential Sources of Bias:

7. Study Design→ 1, 4, 6, 8, 9, 11
8. Study Participation→ 2, 5, 7
9. Study Attrition→ N/A
10. Associated Factors→ 10
11. Outcome Measures/ Confounding Account→ 12
12. Analysis→ 3

Supplementary Table 3 (c) Quality assessment of qualitative studies.

**Study**	1	2	3	4	5	6	7	8	9	10	Score
Mollayeva et al., 2020	Yes	Yes	Yes	Yes	Yes	Can't tell	Yes	Yes	Yes	Can't tell	**8/10 (+)**
Gutman et al. (1996)	Yes	Yes	Yes	Yes	Yes	Can't tell	Can't tell	Yes	Yes	Can't tell	**7/10 (+)**

Yes, Can't tell & No

Criteria:

1. Was there a clear statement of the aims of the research?
2. Is a qualitative methodology appropriate?
3. Was the research design appropriate to address the aims of the research
4. Was the recruitment strategy appropriate to the aims of the research?
5. Was the data collected in a way that addressed the research issue?
6. Has the relationship between researcher and participants been adequately considered?
7. Have ethical issues been taken into consideration?
8. Was the data analysis sufficiently rigorous?
9. Is there a clear statement of findings?
10. How valuable is the research?

Supplementary Table 4: Summary of study characteristics, including details on study sample, design, methods and results on associative values of sex/gender, age and injury severity variables on clinical and non-clinical outcomes.

Author<br>Date<br>Country<br>Gender inequality index (rank).<br>Level of evidence	(1) Objective<br>(2) Design<br>(3) Setting<br>(4) Inclusion/exclusion (IC/EC) criteria	(1) Sample size<br>(2) Attrition<br>(3) Age (mean (SD)/ range), yrs<br>(4) Sex (%M)<br>(5) Time since injury (TSI)<br>(6) Injury severity (IS)<br>(7) Assessment time points/ assessed (AT: t_1, t_2, etc.), if longitudinal<br>(8) Education	Statistical method<br>(1) Approach<br>(2) Level of sign.	1) Confounders<br>2) Medications	Results	
					(1) Outcome of interest<br>(2) Measurement(s) used<br>(3) Frequencies or scores (M vs F)	Sex, Age & IS related
Arellano-Orden et al. (2011)<br><br>Spain<br><br>0.081 (15)<br><br>Level III	(1) To assess role of sex as an independent factor in cerebral oxygenation following RBCT<br>(2) Secondary analysis of a prospective study data<br>(3) Level 1 trauma center<br>(4) IC: pts w/ sev TBI, intraparenchymal ICP & $PbrO_2$ catheter, post-resuscitation, no bleeding, Hb ≤95 g/l, AP >75 mm Hg w/ no or low-dose vasoactive drugs), under	(1) N= 88<br>(2) 0%<br>(3) Age (M;F): 32.8 ± 13.9; 45.5 ± 19.4<br>(4) Sex: 79.5%<br>(5) N.R<br>(6) IS: sev TBI<br>(7) AT:<br>BL: pre-transfusion set (time not specified)<br>t_1: end of transfusion<br>t_2: post-transfusion h 1<br>t_3: post-transfusion h 2<br>t_4: post-transfusion h 3<br>t_5: post-transfusion h 4<br>t_6: post-transfusion h 5<br>t_7: post-transfusion h 6<br>(8) N.R	(1) Kolmogorov–Smirnov test Student's t test MANOVA<br>(2) P≤ 0.05	(1) $PbrO_2$, Sex, Age, Weight, CPP, ISS, & Hb<br>(2) N.R	(1) Cerebral Oxygenation<br>(2) LICOX IMC System<br>(3) $PbOR_2$ Levels following RBCT (M; W):<br>BL: 23.9 ± 9.6; 24.8 ± 9.0<br>t_1: 26.4 ± 9.1; 29.8 ± 11.3<br>t_2: 26.4 ± 9.6; 32.1 ± 12.2<br>t_3: 26.3 ± 9.0; 32.3 ± 13.1<br>t_4: 27.2 ± 9.8; 32.6 ± 13.1<br>t_5: 26.2 ± 9.5; 30.8 ± 11.0<br>t_6: 26.4 ± 9.5; 30.0 ± 10.2<br>t_7: 26.2 ± 9.5; 29.6 ± 11.8	Sex & Age related to the ↑ in $PbrO_2$ suggesting effect of RBCT on<br><br>Cerebral Oxygenation, as measured by PbrO2, is ↑ in F> M<br><br>Age inversely related to the PbrO2 increment in both groups

	ventilation, PaO_2/ FiO_2 ratio>250, &temperature <38▫C<br>(5) EC: Unstable pts (frequent changes in PaO_2, FiO_2 conc, or ventilatory regimen) or surgery w/in few h					
Clond et al. (2011)<br><br>USA<br><br>0.203 (43)<br><br>Level III	(1) To explore assoc btwn sex & SBP in trauma pts w/ Pneumonia & Mortality as outcomes<br>(2) Retrospective comparative study<br>(3) Los Angeles County Trauma System Database<br>(4) IC: ≥ 14 y.o, AD btwn 2003-2008<br>(5) EC: <14, dead on arrival, AIS >5, missing data on: age, sex, AIS, ISS< GCS or blood alcohol status	(1) N=3025 TBI pts (24184 non-TBI)<br>(2) N/A<br>(3) Age **(M;F)**<br>TBI: 44.8 ±21.6; 40.7±17.3<br>Non-TBI: 38.6±15.9; 40.7±19.33<br>(4) Sex (TBI; non-TBI): 79.9%; 76.7%<br>(5) N.R<br>(6) IS:<br>ISS **(M;F)**:<br>TBI: 21.3±11.1; 22.2±11.6<br>Non-TBI: 7.84±7.93; 7.84±8.41<br>AIS head **(M;F)**:<br>TBI: 3.76 ±0.79; 3.77±0.80<br>Non-TBI: 0.49±0.82; 0.48±0.80<br>(7) N/A<br>(8) N.R	(1) MRM Student's t test Wilcoxon rank sum test<br>(2) $P<0.05$	(1) Age, ISS, GCS & SBP<br>(2) N.R	(1) Pneumonia & Mortality<br>(2) Medical records<br>(3) Frequency of Pneumonia (M; F): 8.1% (197/2418); 7.2% (44/607)<br><br>Frequency of Mortality: (M; F): 10.3% (250/2418); 12.2% (74/607)	In M, Age, ISS≥16, GCS≤8 & SBP≥160 were SS predictors of pneumonia<br><br>In F, ISS≥16, GCS≤8 & SBP≥160 were SS predictors of pneumonia<br><br>In M, Age, ISS≥16, GCS≤8, SBP<90 & SBP≥160 were all SS predictors of mortality<br><br>In F Age, ISS≥16, GCS≤8 & SBP<90 were predictors of mortality

Glenn et al. (2001)<br><br>USA<br><br>0.203 (43)<br><br><br>Level IV	(1) To investigate depression and its assoc w/ subject characteristics in outpts w/ TBI<br>(2) Cross-sectional study of non-consecutive pts<br>(3) Outpt clinic<br>(4) IC: ≥16 y.o, RLAS score V or ↑ at time of study participation, no other sign neurologic diagnoses<br>EC: NR	(1) N= 41<br>(2) N/A<br>(3) 43.6±15.2<br>(4) Sex: 66% M<br>(5) TSI (mos): 41.4±58.9<br>(6) IS:<br>Mild: 56%<br>Mod: 17%<br>Sev: 27%<br>(7) N/A<br>(8) N.R	(1) ANOVA OR<br>(2) p<.05	(1)Age, Gender, Violent etiology, mTBI, Antidepressant & Stimulant use<br>(2) Med Use:<br>Antidepressants: 32%<br>Psychostimulants: 24%<br>Dopaminergic: 27%	(1) Depression<br>(2) BDI-II<br>(3) N.R	Gender & mTBI SS associate w outcome<br>Age was NS
Harrison et al. (2006)<br><br>Sweden<br><br>0.048 (4)<br><br>Level III	(1) To investigate assoc btwn previous HI, SCZ & Non-Affective Psychoses<br>(2) Retrospective comparative study (prognostic)<br>(3) Swedish data records<br>(4) IC: NR<br>EC: died, emigrated or Dx of SCZ or other Non-Affective Psychosis <16 y.o, missing values for ≥1 variables, implausible values for birth	(1) N (SCZ; Non-affective psychosis):<br>Cases: 748; 1,526<br>Controls: 14,960; 30,520<br>(2) N/A<br>(3) Age (SCZ; Non-affective psychosis):<br>16-20: 501; 845<br>21-25: 386; 793<br>26+: 87; 171<br>(4) Sex (SCZ; Non-affective psychosis): 67%; 55.4%<br>(5) TSI (SCZ, non-affective psychosis):<br><3yrs: 20.37%; 24.43%<br>4-6: 27.78%; 14.5%<br>7-9: 7.4%; 15.26%<br>10-13: 14.81%; 11.44%<br>14+: 29.64%; 34.37%<br>(6) IS: Sev HI	(1) D/O al LRM<br>(2) P<0.05	(1) Sex, Sev head inj, No inj, Yr of birth, Highest parental income, Highest parental education, Highest parental occupation, Area of birth, Family Hx of psychosis, Apgar score at 5min, Gestational age, Paternal age, Birthweight & Birth length<br>(2) N.R	(1) SCZ & Non-Affective Psychosis<br>(2) Medical records<br>(3) Frequency of SCZ (M;F): 67.0% (501/748); 33.0% (247/748)<br><br>Frequency of Non-Affective Psychosis (M;F): 55.4% (845/ 1526) ; 44.6% (681/1526)	No diff in M & F for SCZ & for other Non-Affective Psychoses<br><br>Interaction w/sex & Sev HI SS predictors of Non-Affective Psychosis not SCZ

		(7) N/A<br>(8) N.R				
Lee et al. (2014)<br><br>Taiwan<br><br>0.164 (37)<br><br>Level II	(1) To evaluate if TBI pts have a ↑ risk of Stroke compared to general population<br>(2) Retrospective cohort study<br>(3) National Health Insurance program database<br>(4) IC: hospitalized for Stroke & diagnosed w/ mTBI<br>EC: pts w/ mTBI & any type of Stroke diagnosed before January 1, 2007, diagnosed w/ Hemorrhagic Stroke during F/U period, previous TBI	(1) N= 24905 (w/mTBI); 719811 (w/o mTBI control)<br>(2) N/A<br>(3) Age (mTBI; control): 46.1±20.1; 43.5±16.3<br>(4) Sex (mTBI; control):47.4%; 48.5%<br>(5) TSI mTBI; (mean F/U yr): 1.94±1.18<br>(6) IS: mTBI<br>(7) N/A<br>(8) N.R	(1) Pearson's chi-square<br>t test<br>Cox proportional HR model<br>(2) p<0.05	(1) Age, Sex, Urbanization level, SES, Diabetes, Hypertension, Coronary Artery Dx, Hyperlipidemia, Hx of alcohol intoxication, Malignancies, Heart Failure, Atrial Fibrillation, Smoking, Obesity, Epilepsy, Peripheral Artery Dx & CCI<br>(2) N.R	(1) Stroke<br>(2) Medical records<br>(3) N.R	HR of Ischemic Stroke:<br>Age: (HR= 1.07; 95%CI =1.06-1.07; P= <0.001)<br>Sex: (HR= 1.46; 95% CI= 1.40-1.52; P= <0.001)<br>Older age, Sex (M) & Severity assoc w/ ↑ risks of Ischemic Stroke in TBI
Meares et al. (2007)<br><br>Australia<br><br>0.120 (24)<br><br>Level IV	(1) To examine predictors of acute PCS<br>(2) Case-control study<br>(3) Level 1 trauma hospital<br>(4) IC: hospital AD w/in 24h of inj, initial assessment not > than 14d, btwn 18-65 y.o, IQ >70, not subject of forensic investigation, no	(1) N= 90 mTBI; 85 controls<br>(2) N/A<br>(3) Age (mTBI; controls): 34.7±13.7; 34.6±12.1<br>(4) Sex (mTBI; controls): 67.8%; 64.7%<br>(5) TSI: w/in 14d post-inj<br>(6) ISS (mTBI; controls): 5.4±6.2; 5.5±5.3<br>GCS (mTBI):<br>13: 2.2%<br>14: 12.1%<br>15: 85.7%<br>Duration of PTA (mTBI):	(1) LRM<br>(2) P<0.05	(1) Age, Sex, IQ, mTBI, CLTR, SDMT-oral, SEQRT1, ASDS-DISS, ASDS-total, AXIS-1, SUBS & Pain<br>(2) Med Intake:<br>Opioid Analgesics: 56.7%<br>Marijuana: 25.6%	(1) PCS<br>(2) PCSC & MINI<br>(3) N.R	Sex (F) was a SS predictors of acute PCS while Age & mTBI were not

	evidence of pre-existing cog impairment, sufficient English language comprehension, no Psychosis, no physical inj as a result of self-harm, medically able to participate, not being interstate or overseas visitor, not pregnant<br>EC: trauma related lesion present on CT scan regarded as mod TBI	<5min: 36.7%<br>6-60min: 22.2%<br>61min-12h: 23.3%<br>>12h-24h: 17.8%<br>(7) N/A<br>(8) Education duration in yrs (mTBI; controls): 11.5±2.8; 11.5±2.5				
Miles et al. (2015)<br><br>USA<br><br>0.203 (43)<br><br>Level IV	(1) To examine impact of mTBI on PTSD symptoms, frequency of AUD & influence of PTSD on AUD in veterans<br>(2) Case series<br>(3) Veteran medical center<br>(4) IC: N.R<br>EC: Veterans who were not given the TBI screen	(1) N= 1278<br>(2) N/A<br>(3) Age: 29.75±7.71<br>(4) Sex: 88.7%<br>(5) N.R<br>(6) IS: mTBI<br>(7) N/A<br>(8) Education<br>High School/GED: 32.3%<br>College (1-3yrs): 55.7%<br>Completed College: 12.0%	(1) t test<br>LRM<br>(2) P<0.05	(1) Age, Sex, Severity & PTSD<br>(2) N.R	(1) AUD<br>(2) Medical records & clinical interview<br>(3) Frequency of AUD (M; F): 25.9% (97/374); 3.6% (1/28)	In M, PTSD was a SS predictor of AUD;<br>Age, mTBI were NS<br><br>In F, neither mTBI or PTSD were SS predictors for AUD (possibly due to ↓ frequency of F)
Mollayeva et al. (2016)<br><br>Canada<br><br>0.098 (18)	(1) To evaluate the presence of Insomnia in works w/ delayed recovery from TBI and its relationship w/ other factors	(1) N=94<br>(2) N/A<br>(3) Age: 45.20±9.94<br>(4) Sex: 61.7%<br>(5) TSI: 509d (median=197d)<br>(6) IS: mTBI	(1) Spearman's correlation<br>ANOVA<br>Residual plots<br>Stepwise MRM<br>(2) P≤0.10 &	(1) Variables w/in: Sociodemographic, Claim-related, Family-related, Social, Inj-related, Medical,	(1) Insomnia<br>(2) ISI<br>(3) ISI Score (M;F): 17.09 ± 6.40; 17.83 ± 5.68	Age, Previous head trauma were SS predictors of outcome in final model<br>Sex NS

Level IV	(2) Cross-sectional<br>(3) Worker's Safety & Insurance Board Clinic at Toronto Rehab-UHN<br>(4) IC: ≥18y.o, injured at work<br>EC: N.R	(7) N/A<br>(8) Education:<br>≤High school: 36%<br>High school-college, professional diploma: 34%<br>≥University: 27%	P≤0.05 (final model)	Psychiatric, Comorbid sleep D/Os, Other clinical, Med/Substance effects & Behavioral<br><br>(2) Med Intake:<br>Antihistamines: 26%<br>Benzodiazepines: 14%<br>Narcotic analgesics: 24%<br>TCA: 29%<br>SSRI: 16%		
Mollayeva et al. (2016)<br><br>Canada<br><br>0.098 (18)<br><br>Level IV	(1) To examine the relationship btwn Insomnia & perceived Disability in workers w/ mTBI/Concussion<br>(2) Cross-sectional<br>(3) Worker's Safety & Insurance Board Clinic at Toronto Rehab-UHN<br>(4) IC: ≥18y.o, injured at work, diagnosed w/ mTBI/Concussion<br>EC: N.R	(1) N=92 (58 Marked/Extreme Disability & 34 Mild/Mod Disability)<br>(2) N/A<br>(3) Age (Marked/Extreme; Mild/Mod): 45.60±9.70; 44.40±10.40<br>(4) Sex (Marked/Extreme; Mild/Mod): 65.2%; 52.9%<br>(5) TSI: median 196d (138-412d)<br>(6) IS: mTBI/Concussion<br>(7) N/A<br>(8) Education (Marked/Extreme; Mild/Mod):<br>≤High school: 36.2%; 38.2%<br>> High school: 62.1%; 58.8%<br>Missing: 1.7%; 2.9%	(1) Two-sided t test<br>Chi-squared tests<br>Spearman's correlation<br>ANOVA<br>Residual plots<br>Stepwise MRM<br>(2) P≤0.05	(1) Insomnia, Age, Sex, Depression, Pain, Anxiety & Work status<br>(2) N.R	(1) Perceived Disability<br>(2) ISI, PHQ-9, P-VAS, HADS-A<br>(3) Frequency of Global Disability-Marked/Extreme (M;F): 65.2% (38/58); 34.8% (20/58)<br><br>Mild/Mod (M;F): 52.9% (18/34); 46.1% (16/34)	Sex & Age NS associate w/ Global, Work, Social or Family/ Home Disability

Mollayeva et al. (2017)<br><br>Canada<br><br>0.098 (18)<br><br>Level IV	(1) To examine sex diff in construct of chronic pain in pts with delayed recovery from concussion/mTBI<br>(2) Cross-sectional<br>(3) Worker's Safety & Insurance Board Clinic at Toronto Rehab-UHN<br>(4) IC: pts w/ concussion/ mTBI Dx<br>EC: w/o brain injury Dx, brain injury due to electrocution, mod-sev TBI	(1) N=94<br>(2) N/A<br>(3) Age: 45.20±9.94<br>(4) Sex: 62%<br>(5) TSI: median 197d (139-416)<br>(6) IS: mTBI<br>(7) N/A<br>(8) Education:<br>≤High school: 36%<br>High school-college, professional diploma, university or higher: 64%	(1) Spearman's correlation<br>ANOVA<br>Residual plots<br>Stepwise MRM<br>(2) P≤0.05	(1) Variables w/in: Cultural/Social, Behavioral/Environ mental, Psychological/ Pathopsychologivc al, Physiological/ Brain inj related & Physical/ Med effect<br><br>(2) Med Intake<br>Antihistamines: 26%<br>Benzodiazepines: 13%<br>Narcotic analgesics: 22%<br>Tricyclic antidepressants: 29%<br>SSRI: 15%<br>Recreational substance use: 12%	(1) Pain Severity<br>(2) P-VAS<br>(3) Pain Severity Score (M;F): 16.33±6.23; 15.14±6.62	Sex, Age NS associate w pain severity<br><br>Sex-specific multivariate models perform better:<br><br>Fully adj final generic model explained 34.5% of variance of pain severity<br>M-specific fully adj model explained 60% of variance of pain<br>F-specific fully adj model explained 46.2% of variance
Mollayeva et al. (2017)<br><br>Canada<br><br>0.098 (18)<br><br>Level III	(1) To examine deviation in sleep stage distribution in persons w/ mTBI<br>(2) Case-control study<br>(3) Neurology services of Toronto Rehab-UHN<br>(4) IC: mTBI Dx & PSG data	(1) N= 39<br>(2) N/A<br>(3)Age: 47.54 ± 11.30<br>(4)Sex: 56%<br>(5) TSI: 799 (9008/135)<br>(6) IS: mTBI<br>(7) N/A<br>(8) Education<br>≤ High school: 38%<br>> High school: 62%	(1) Kolmogorove-Smirnov test<br>Spearman's correlation coefficient<br>ANOVA<br>MRM<br>(2) P≤0.05	(1) Adjustment D/O, Symptom load, Body mass index, Tension w/ employer or insurer, RDI in REM, SSRI use, Insomnia, Cog D/O, Education> high school, Mechanism of inj, RDI in non REM,	(1) Change in Nocturnal Wakefulness, N1, N2, N3 (stage 3&4) & REM in ref to age (per 5-year ↑) & sex specific norms<br>(2) PSG<br>(3) Nocturnal Wakefulness (M &W vs. pop norms):<br>M: 12.69 ± 14.13<br>W: 12.15 ± 9.49<br><br>N2 (M &F vs. pop norms):	Sex NS associate with outcome<br><br>Age & LOC and/or PTA SS for change in N3(stage 3) & N3 (stage 4) respectively

	EC: pts w/o brain inj, inj due to electrocution, no evidence of sleep D/O requiring PSG, mod-sev TBI, sleep efficiency during PSG of <30			Cluster B personality trait, Musculoskeletal D/O, LOC and/or PTA, Wakefulness proceeding PSG, Benzodiazepines use & REM periods<br>(2) Med Intake (d preceding PSG):<br>Benzodiazepines: 18%<br>Narcotic analgesics: 28%<br>TCA: 36%<br>SSRIs: 23%	M: -2.97 ± 14.99<br>F: -5.77 ± 9.27<br><br>REM (M &F vs. pop norms):<br>M: -6.38 ± 5.73<br>F: -9.96 ± 7.45	
Mollayeva et al. (2019)<br><br>Canada<br><br>0.098 (18)<br><br>Level II	(1) To evaluate whether the risk of incident of dementia was related to severity & extent of CNS trauma differently in M & F<br>(2) Retrospective cohort w multiway sensitivity analyses<br>(3) Institute for Clinical Evaluative Sciences Database<br>(4) IC: Adults ≥18y.o, Ontario residents, Dx of CNS trauma in an ED or acute care<br>EC: first TBI before Apr 1, 2003,	(1) N= 712,708 (32,834 Dementia; 679,874 No Dementia)<br>(2) N/A<br>(3) Age at first TBI:<br>Total: 44.00 (27.00-64.00)<br>Dementia: 82.00 (76.00-87.00)<br>No Dementia: 42.00 (26.00-63.00)<br>(4) Sex (Total; Dementia; No Dementia): 59.44%; 40.63%; 60.35%<br>(5) N.R<br>(6) IS (n of total pts):<br>Mild: 443,456<br>Moderate: 7,365<br>Severe: 17,390<br>Unspecified: 244,497<br>(7) N/A<br>(8) N.R	(1) Univariate & multivariable Cox regression models<br>HR<br>Fine and Gray competing risk regression model<br>(2) $P<0.05$	(1) TBI severity, SCI, sex, age, income, cerebrovascular D/O, ischemic heart D/O, D/O vessels, atrial fibrillation, heart failure, obesity, tobacco smoking, hyperlipidemia, diabetes, depression, vision impairments, hearing loss, sleep D/O<br>(2) N.R	(1) Dementia onset<br>(2) Medical records<br>(3) Frequency of Dementia (M; F): 40.63%; 60.35%	Sex/gender associate with dementia RR (male sex at greater risk)<br><br>Age interacted with TBI severity to ↑RR (younger pts greater RR)<br><br>Severe TBI and concussive injury ↑ RR<br><br>Sex-stratified model: female patients develop dementia earlier than male patients all other risk factors being equal

	dementia Dx < 1 yr prior to TBI & <3 yrs post first TBI, pts w/ delirium					
Podoga et al. (2012)<br><br>USA<br><br>0.203 (43)<br><br>Level III	(1) To identify the co-occurrence of MSI & examine characteristics that were potentially predictive of MSI (sensory, auditory & visual impairments)<br>(2) Case-control study<br>(3) Veteran medical center<br>(4) IC: no brain inj preceding employment or post-deployment, met mTBI Hx based on VA-DOD clinical practice guidelines<br>EC: N.R	(1) N= 13746 (9998 mTBI; 3748 no mTBI)<br>(2) N/A<br>(3) Age (mTBI; no mTBI):<br>18-24: 23.7%; 16.1%<br>25-29: 35.9%; 30.0%<br>30-39: 23.8%; 26.3%<br>≥40: 16.6%; 27.6%<br>(4) Sex (mTBI; no mTBI): 95.0%; 91.6%<br>(5) N.R<br>(6) IS: mTBI<br>(7) N/A<br>(8) N.R	(1) Chi-square test<br>Independent t-test<br>LRM<br>(2) P<0.05	(1) Age, Sex, Branch of service, Rank, Etiology of inj, mTBI Hx, PTSD & Depression<br>(2) N.R	(1) MSI<br>(2) Self-report<br>(3) N.R	Age, Sex (F) & mTBI Hx SS predictive of MSI (both models)
Sutton et al. (2019)<br><br>Canada<br><br>0.098 (18)<br><br>Level III	(1) To study sex diff in rate of neck inj comorbidity in pts with a concussion<br>(2) Retrospective comparative study<br>(3) National Ambulatory Care Reporting System<br>(4) IC: Pts identified w/ ICD S06.0 code & those	(1) N=90,175 (8,134 MVC related)<br>(2) N/A<br>(3) Mean age N.R<br>(4) Sex (All concussions; MVC related): 58.13%; 53.2%<br>(5) N.R<br>(6) IS: mTBI<br>(7) N/A<br>(8) N.R	1) MLRM<br>2) OR<br>3) P<0.05	1) Sex, Age, Mechanism of injury, Income, Rurality, Sports injury, Fiscal year of ED, DC, Intention of Inj<br>2) N.R	1) Comorbid neck injury<br>2) Medical records<br>3) Frequency of comorbid neck inj (M; F):<br>4) All concusions: 58.13%; 41.87%<br>5) MVC: 53.2%; 46.8%	For the All concussions:<br>M group age ranges 15-19, 20-24, 25-29, 30-34, 45-49, 50-54, 55-59, 60-64 & 65-69 were SS<br>F group age ranges 15-19, 20-24, 25-29, 35-39, 40-44, 45-49, 50-54, 55-59, 60-64,

	w/ mild inj severity (<3 AIS)<br>EC: Superficial inj (S10), open wounds (S11) amputation (S18)					65- 69 & 70-74 were SS<br><br>For the MVC concussion:<br>M group age ranges 15-19, 20-24 & 30-34 were SS<br><br>F group age ranges 15-19, 20-24, 25-29, 45-49, 50-54, 55-59 & 60-64 were SS
Tawil et al. (2007)<br><br>USA<br><br>0.203 (43)<br><br>Level IV	(1) To define the incidence & Mortality impact of PTCI & risk factors assoc w/ PTCI<br>(2) Case series<br>(3) Trauma/critical care center<br>(4) IC: sev TBI, GCS <9, AIS >2, survive beyond 24h<br>EC: pts w/ CT evidence of diffuse cerebral infarction b/c of Hypoperfusion, primary CVA, or Strokes secondary to angiographic complications	(1) N=384 Total (31/ 384= PTCI only)<br>(2) N/A<br>(3) Age (PTCI): 36 (11–90)<br>(4) Sex (All TBI including PTCI): 75%<br>(5) N.R<br>(6) IS: sev TBI<br>(7) N/A<br>(8) N.R	(1) Chi-square test<br>Student's t test<br>LRM<br>(2) P< 0.05	(1) Sex, Age, ISS, TRISS, RTS, AD GCS, Brain AIS, Craniotomy, BCVI, Factor VIIa<br>(2) N.R	(1) PTCI<br>(2) CT scan, MRI & Cerebral angiogram<br>(3) N.R	Sex, Age, ISS, RTS, AD GCS, & Brain AIS NS associate w PTCI
van der Horn et al. (2013)	(1) To investigate the relation of Postconcussive symptoms, Anxiety,	(1) N= 242 (51 Minor; 121 Mild; 70 Mod-Sev)<br>(2) N/A	(1) Spearman correlation coefficient<br>Chi-square test	(1) GCS score, Postconcussive symp, Anxiety & Depression	(1) RTW<br>(2) RTW Questionnaire	Sex, Age NS<br>IS, Postconcussive symp, Anxiety, & Depression were all

Netherlands<br><br>0.044 (3)<br><br>Level IV	& Depression w/ vocational outcome in pts w/ TBI<br>(2) Case series<br>(3) Level 1 trauma center<br>(4) IC: pts w/ TBI admitted to University Medical Centre Gronnigen<br>EC: <15 y.o, previous TBI	(3) Age (Minor, Mild, Mod-Sev): 42.1±16.8; 40.3±17.3; 35.2±18.8<br>(4) Sex(Minor, Mild, Mod-Sev): 61%; 78%; 73%;<br>(5) N.R<br>(6) IS: 51 Minor; 121 Mild; 70 Mod-Sev<br>(7) N/A<br>(8) N.R	Binary LRM<br>MLRM<br>(2) P< 0.05	(2) N.R	(3) Frequency of RTW (Complete RTW reported*):<br>Minor (M;F): 82%; 50%<br>Mild (M;F): 72%; 55%<br>Mod-Sev (M;F): 44%; 47%<br>Total (M;F): 66%; 51%	SS predictive of RTW (explained variance 45%) adj for Age & Sex
Wang et al. (2008)<br><br>Taiwan<br><br>0.164 (37)<br><br>Level IV	(1) To study the clinical relevance of PTA & other neurologic complications that occur after TBI & the potential prognostic factors of Posttraumatic Seizures<br>(2) Case series<br>(3) Acute care teaching medical hospital<br>(4) IC: seizure after head trauma<br>EC: Hx of TBI, pre-existing Epilepsy, gunshot wounds or penetrating HI, neurologic D/Os w/ various neurologic deficits & death w/in 1 mo	(1) N=170 (134= Excellent outcome; 36= Non-excellent outcome)<br>(2) Attrition :0%<br>(3) Age (Excellent; Non-Excellent): 40.5 ±24.7; 44.6±20.9<br>(4) Sex (Excellent; Non-Excellent): 71.6%; 83.3%<br>(5) N.R<br>(6) IS (Excellent; Non-Excellent) (N):<br>Mild: 19; 6<br>Mod: 10; 1<br>Sev: 105; 29<br><br>Clinical features-<br>PTA: 94; 23<br>Brief unconsciousness: 72; 20<br>GCS at ad: 10.5±3.9; 10.4±3.7<br>(7) BL: EMD<br>F/U: 1mo-204 mos post-inj<br>(8) N.R	(1) Chi-square test<br>Fisher's exact test<br>Student's t test<br>Wilcoxon rank sum test<br>Log-rank test<br>Stepwise LRM<br>(2) P <0.10	(1) Age, Sex, Clinical features, Severity, GCS, Neuroimaging, Onset of seizure, Subtype of seizure, Surgical intervention & Duration of hospitalization<br>(2) Med Intake: most pts in were treated w/ anticonvulsant meds after 1st acute symptomatic seizure	(1) Outcomes of Posttraumatic Seizures (based on seizure frequency)<br>(2) CT scan & Medical evaluation<br>Note: by Engel et al (score<5-excellent; 5-12-non-excellent)<br>(3) Frequency of Excellent Outcome (M;F): 71.6% (96/134); 28.4% (38/134)<br><br>Frequency of Non-Excellent Outcome (M;F): 83.3% (30/36); 16.7% (6/36)	Age, Sex & Severity NS associate w seizure frequency

Eramudugolla et al. (2014)<br><br>Australia<br><br>0.120 (24)<br><br>Level IV	(1) To examine a range of TBI variables as predictors of cog outcome over 8 yrs in an adult pop, & interactions w/ APOE genotype, sex, & age cohorts<br>(2) Case series<br>(3) PATH Project<br>(4) IC: extensive PATH criteria reported in another article<br>EC: Hx of cerebrovascular events or Epilepsy at BL	(1) N= 6333<br>20s cohort: 2077<br>40s cohort: 2124<br>60s cohort: 2132<br>(2) Attrition (Wave 1; Wave 2): 6.5%; 16%<br>(3) Age (20; 40; 60 cohort): 22.61±1.51; 42.62±1.49; 62.51±1.51<br>(4) Sex(20; 40; 60 cohort): 46.7%; 46.1%; 50.5%<br>(5) N.R<br>(6) IS (20; 40; 60 cohort):<br>Mild: 8.5%; 6.0%; 3.1%<br>Mod/Sev: 90.0%; 91.4%;<br>(7) N/A?<br>(8) Education (20; 40; 60 cohort): 14.21±1.50; 14.43±2.28; 13.90±2.74<br>Note: Note: TBI ensured by pt self-report	(1) Linear mixed-effects models<br>(2) P< 0.05	(1) Sex, Age, Severity & Frequency<br>(2) N.R	(1) Cog functioning<br>(2) CVLT, STW, SDMT, DST & Simple Reaction Time<br>(3) Reaction Time T-Score (M; F): ~57.5; ~54	Cog performance ↑ for young F (in 20s) > M; pattern reversed in middle (40s) & old age (60s)
Gerhart et al. (2003)<br><br>USA<br><br>0.203 (43)<br><br>Level III	(1) To examine characteristics, circumstances of inj, tx pathways, & outcomes of persons w/ TBI as a result of violence in comparison to other TBI survivors<br>(2) Retrospective comparative study<br>(3) State wide hospital records<br>(4) IC: ≥16 y.o, resident of state,	(1) N= 1802 (112= Violent TBI; 1689= Other TBI)<br>(2) N/A<br>(3) Age (Violent; Other):<br>16-24: 30.4%; 26.8%<br>25-34: 25.9%; 18.1%<br>35-44: 21.4 %; 18.7%<br>45-54: 6.3%; 12.7%<br>55-64: 7.1%; 7.0%<br>65+: 8.9%; 16.6%<br>(4) Sex (Violent; Other): 83.0%; 66.7%<br>(5) TSI: 1 yr post-inj<br>(6) IS:<br>Sev (GCS 3–8): 17.0%; 12.7%	(1) Chi-square test<br>ANOVA<br>LRM<br>(2) P< 0.01	(1) Violence, Severity, ISS, Age, Sex, Ethnicity, Working status, Relationship status, Gov funding<br>(2) Pre-ink Drug Intake:<br>Total: 4,1%<br>Violently Incurred TBI: 7.8%<br>All Other TBIs: 3.9%	(1) Cog symptomatology, Functional & Societal participation<br>(2) SIP-AB subscale & CHART-SF<br>(3) N.R	SIP-AB SS: Mod inj, ISS, Age, & Sex (F)<br><br>CHART-SF- Physical independence predictive factors: Sev inj<br><br>CHART-SF Cog independence predictive factors: Sev inj, Mod inj, Age & Sex (W)

	hospitalized w/ newly incurred TBI<br>EC: no documentation of an eligible TBI (according to CDC criteria), did not meet age or residency criteria	Mod (GCS 9–12): 7.1%; 7.0%<br>Mild (GCS 13–15): 75.9%; 80.3%<br>(7) N/A<br>(8) N.R				CHART-SF Mobility: Sev inj, Age & Sex (W)<br><br>CHART-SF Occupation SS: Sev inj, ISS, Age & Sex (W)<br><br>CHART-SF Social integration SS: Sev &Mod inj<br><br>CHART-SF Economic self-sufficiency SS: Mod inj & Age<br><br>CHART-SF total SS: Sev inj, Mod inj, ISS, Age & Sex (W)
Jung et al. (2014)<br><br>Korea<br><br>0.067 (10)<br><br>Level IV	(1) To explore the relationship in demographic & clinical variables, neurocognitive, & functional outcome that could be used as prognostic factor for old aged pts w/ TBI<br>(2) Case series<br>(3) Multiple hospitals<br>(4) IC: received hospital or ambulant tx for brain inj	(1) 162 (85= Junior-ES; 77= Senior-ES)<br>(2) N/A<br>(3) Age :<br>55-59: 20.4%;<br>60-64: 32.1%;<br>65-69: 24.1%<br>70-74: 13.6%<br>75-80: 9.9%<br>Mean: 60.16±3.08; 70.38±4.37<br>(4) Sex: 71.0%<br>(5) TSI: 20.90± 34.92 mos<br>(6) IS:<br>LOC: 64.8%<br>(7) N/A	(1) ANOVA<br>MLRM<br>LRM<br>(2) P<0.05	(1) Sex, Age, Education, Residence, Cause of visit, LOC, Comorbidity, Operation, MMSE, Attention, Confrontational naming, Visuospatial functioning, Verbal memory, Visual memory, Generative naming	(1) Functional & Neurocognitive outcomes<br>(2) SNSB, MMSE, DST, Korean version of Boston Naming Test, ROCF, Stroop Test & CDR<br>(3) Mean Neurocognitive Scores (M;F):<br>MMSE<br>Low score: 19.03±6.52; 14.21±7.31<br>Standard score:<br>-4.71±4.16; -5.98±4.49<br>Attention: -1.25±1.58; -1.65±2.66	Sex SS effect on MMSE score, Confrontational naming, Generative naming & CDR<br><br>Age SS effect on Verbal memory<br><br>LOC SS effect on CDR<br><br>For functional outcome: Age, Sex & LOC NS

	EC: pre-injury neurological D/Os, cannot complete did neurocognitive function tests due to TBI	(8) Education: 6.01±4.54 yrs		& Inhibitory control<br>(2) N.R	Confrontational naming: -1.81±2.28; -3.13±1.87<br>Visuospatial functioning: -4.72±4.23; -5.10±4.82<br>Verbal memory: -2.01±1.20; -2.23±0.96<br>Visual memory: -1.69±1.16; -1.94±0.83<br>Generative naming: -1.59±0.94; -1.95±0.71<br>Inhibitory control: -2.84±1.60; -3.39±1.54<br>CDR score: 1.51±0.91; 1.82±1.10	
Mellick et al. (2003)<br><br>USA<br><br>0.203 (43)<br><br>Level IV	(1) To identify the factors that determine the pathways of care people w/ TBI follow after acute care DC, & to identify diff in outcome based on those pathways<br>(2) Case-control study w/ 1 yr look back<br>(3) Statewide, population-based Colorado TBI Registry & F/U System<br>(4) IC: ≥16 y.o, DC from acute care in 1996- 1997, eligible for inclusion in the statewide, population-based TBI	(1) N= 1802 (weighted sample corrected for non-respondents)<br>(2) Attrition: 41.9%<br>(3) Age: 40.6±19.9<br><25: 27.0&<br>25-44: 37.3%<br>45-64: 19.4%<br>>65: 16.2%<br>(4) Sex: 67.7%<br>(5) N.R<br>(6) IS:<br>GCS-<br>Mild: 80.0%<br>Mod: 7.0%<br>Sev: 13.0%<br>(7) AT:<br>BL: U/S<br>F/U: 12-18 mos post-inj<br>(8) N.R	(1) Chi-square test<br>ANOVA<br>LRM<br>(2) P < 0.05	(1) Severity, Age, Sex, Minority, Working, Gov funding & Pathway of care<br>(2) N.R	(1) Pt Care Pathway, Physical independence, Community integration, Cog symptomology & Health perception<br>(2) Interview, FIM, CHART, SIP, HSQ-12<br>(3) Frequency of Care Pathways (M;F):<br>Inpt rehab then home/community: 7.2%; 8.8%<br>Inpt rehab then home/community w/ outpt services: 8.7%; 7.2%<br>Inpt rehab then to LTC: 2.2%; 3.5%<br>LTC- no inpt rehab: 2.8%; 4.7%<br>Directly to home/community: 67.0%; 59.1%	Sex SS associate w Rehab Services, Physical independence, Community integration, Cog symptomatology & Health perception<br><br>Age SS factor for all pathways of care, Physical independence & Cog symptomology<br><br>Sev inj SS factors for all outcomes of interest; Mod inj severity SS for Rehab, LTC, Physical independence &

	F/U system, sev TBI or were in a 20% random sample w/ milder inj, consented to participate in a 1 yr post-inj F/U survey<br>EC: Homebound nursing services & visits to physicians				Directly to home/community w/ outpt services: 12.0%; 16.7%<br><br>(% w/ respect to total pop-based on weighted frequency)	Community integration
Mygra et al. (2016)<br><br>USA<br><br>0.203 (43)<br><br>Level III	(1) To investigate whether sex & DA-gene interactions influence cog post-TBI<br>(2) Prospective study of non-consequtive patients<br>(3) Inpt & outpt clinics at level 1 trauma center<br>(4) IC: non-penetrating TBI, AD GCS ≤8 (sev), CT w/ evidence of Intracranial inj, 16-75 y.o<br>EC: N.R	(1) N -Variant (Grouping):<br>ANKK1 = 181 (Hetero=66; Homo=115)<br><br>DRD2= 173 (C/C=96; G-Carr=77)<br><br>COMT= 171 (Val Car=121; Met/Met= 50)<br><br>VMAT= 182 (G-Carr=86; C/C=96)<br><br>DAT= 174 (10/10=75; 9-Carr=99)<br>(2) N.R<br>(3) Age:<br>ANKK1<br>Hetero: 33.30 ± 13.45<br>Homo: 32.61 ± 13.26<br>DRD2<br>C/C: 33.95 ± 13.51<br>G-Carr: 31.64 ± 13.06<br>COMT<br>Val Carr: 32.47 ± 13.41<br>Met/Met: 33.30 ± 13.14<br>VMAT	(1) Mann-Whitney<br>ANOVA<br>Kruskal-Wallis, t tests<br>Chi-square test as appropriate.<br>Spearman or Pearson correlation<br>Multivariate model<br>(2) $P \leq 0.05$	(1) Age, Sex, GCS, Education & GRS<br>(2) N.R	(1) Cog<br>(2) D-KEFS, COWAT, TMT, DST, WAIS, CVLT-II, ROCF & Stroop Interference<br>(3) N.R	sex-specific risk allele assignment/weighting noted:<br><br>SS sex × gene interaction observed at 6 & 12 mos for ANKK1 rs1800497 & COMT rs4680 DRD2 rs6279 & VMAT genotypes independently assoc w/ cog at 6 mos, w/ trends for a sex × gene interaction at 12 mos

		G-Carr: 33.43 ± 14.01<br>C/C: 32.23 ± 12.65<br>DAT<br>10/10: 32.65 ± 12.69<br>9-Carr: 33.10 ± 13.97<br>(4) Sex: (unclear) % W<br>ANKK1<br>Hetero: 19.69%<br>Homo: 18.26%<br>DRD2<br>C/C: 14.58%<br>G-Carr: 22.08%<br>COMT<br>Val Carr: 20.67%<br>Met/Met: 12.00%<br>VMAT<br>G-Carr: 20.93%<br>C/C: 16.67%<br>DAT<br>10/10: 17.33%<br>9-Carr: 20.20%<br>(5) TSI: w/in 24h post-inj<br>(6) IS:<br>GCS: 7 (all groups)<br>(7) AT:<br>BL: 6mos<br>F/U: 12 mos<br>(8) Education:<br>ANKK1<br>Hetero: 12.56 ± 1.82<br>Homo: 12.76 ± 1.86<br>DRD2<br>C/C: 12.93 ± 2.00<br>G-Carr: 12.50 ± 1.71<br>COMT<br>Val Carr: 12.68 ± 1.81				

		Met/Met: 12.64 ± 1.98<br>VMAT<br>G-Carr: 12.72 ± 1.76<br>C/C: 12.69 ± 1.93<br>DAT<br>10/10: 12.56 ± 1.70<br>9-Carr: 12.87 ± 1.85				
Tsushima et al. (2009)<br><br>USA<br><br>0.203 (43)<br><br>Level IV	(1) To investigate possible sex diff in neuropsychological functioning in pts following mTBI<br>(2) Case series<br>(3) Private medical center<br>(4) IC: sustained a mTBI, confusion or disorientation, LOC ≤30; PTA <24h, focal signs, seizure & intracranial lesion, GCS score 13–15 >30 mins post-inj, not be due to drugs, alcohol or med, caused by other inj or tx for other inj (e.g. systemic inj, facial inj or intubation), caused by other problems or penetrating Craniocerebral inj<br>EC: N.R	(1) N= 102<br>(2) N/A<br>(3) Age (M; W): 42.08±15.23; 41.85±14.07<br>(4) Sex: 60.78%<br>(5) TSI in mos **(M;F)**: 22.37±20.42; 25.39±23.88<br>(6) IS: mTBI<br>(7) N/A<br>(8) Education **(M;F)**: 13.49±2.19; 14.45 ±3.21 yrs	(1) T tests MANOVA<br>(2) P ≤0.05	(1) Age, Education, Mos post-inj & Sex<br>(2) N.R	(1) Neuropsychological outcome<br>(2) Halstead-Reitan Neuropsychological Test Battery, WAIS-III, WMS-III, Wide Range Achievement 2 & 3, Dynamometer, Purdue Pegboard & Grooved Pegboard<br>(3) Neuropsychological Scores (M;F):<br>Finger Tapping Dominant 33.84±12.66; 35.30±9.16<br>Finger Tapping Non-dominant 32.50±12.90; 37.20±12.62<br>Speech Sounds Perception 10.36±7.58; 7.26±4.42<br>Seashore Rhythm 4.98±3.18; 4.68±2.77<br>Tactual Performance Time 16.69±8.54; 17.89±7.27<br>Tactual Performance Memory 7.60 ±1.99; 7.16±2.80	NS effect of Sex, Mos post-inj or Sex-by-mos post-inj on outcome<br><br>SS Age & Sex-by-age effect

					Tactual Performance Location 3.49±2.35; 3.47± 2.23<br>TMT A 34.79±15.31; 39.92±29.80<br>TMT B 86.69±39.32; 82.15±39.32<br>Category 33.79±18.88; 41.42±24.7	
Corrigan et al. (2007)<br><br>USA<br><br>0.203 (43)<br><br><br>Level II	(1) To determine whether there are sex diff in employment 1 yr after TBI<br>(2) Prospective cohort study, secondary data analyses<br>(3) Acute care hospitals in South Carolina & TBIMS rehab centers<br>(4) IC: TBI, informed consent, ≥16 y.o, inpt rehab, working<br>EC: N.R	(1) N= 3444 (327=↑ h; 1257= same h; 443=↓ h; 1417= stop working)<br>(2) N/A<br>(3) Age (↑ h; same h; ↓ h; stop working): 30.2±12.0; 37.2±12.7; 34.4±12.1; 35.6±12.4<br>55-64 (%): 4.9; 11.0; 7.5; 9.4<br>45-54 (%):10.1; 20.9; 15.6; 15.4<br>35-44 (%):17.1; 23.9; 25.3; 25.8<br>25-34 (%):21.7; 22.0; 22.1; 23.6<br>20-24 (%):26.6; 15.5; 20.5; 18.1<br>18-19 (%):19.6; 6.8; 9.0; 7.6<br>(4) Sex (↑ h; same h; ↓ h; stop working): 74.0%; 70.5%; 67.0%; 75.0%<br>(5) TSI: 1 yr post-inj<br>(6) IS (↑ h; same h; ↓ h; stop working):<br>AIS (%)-<br>5: 6.7; 10.3; 9.5; 15.7<br>4: 45.9; 49.4; 50.1; 54.6	(1) Chi-square test<br>Multivariable model<br>Polychotomous logistic regression<br>(2) P <0.05	(1) Age, Sex, Race, Education, Marital status, Payer group, AIS, CT group, LOS, Preinj employment<br>(2) N.R	(1) Employment status<br>(2) Interview<br>(3) Frequency of Employment Status (M;F):<br>↑ h: 74.0% (242/327); 26.0% (85/ 327)<br><br>Same h: 70.5% (886/1257); 29.5% (371/1257)<br><br>↓ h: 67.0% (297/443); 33.0% (146/443)<br><br>Stop working: 75.0% (1063/1417); 25.0% (354/1417)	↑h: Age (55-64, 45-54, 35-44), Sex, AIS 5, Sex-by-age & Sex-by-marital status SS<br><br>Same h: Age (45-54, 25-34, 20-24), Sex, Sex-by-age (20-24 only), & Sex-by-marital status SS<br><br>↓h & Still working: Age (55-64) SS

		3: 22.0; 16.1; 19.6; 14.4<br>2: 25.4; 24.2; 20.8; 15.4;<br><br>CT group-<br>Unknown: 9.5; 13.9; 9.3; 9.7<br>Abnormal: 68.5; 65.0; 68.0; 76.7<br>Normal: 22.0; 21.2; 22.8; 13.6;<br>(7) N/A<br>(8) Education (%) (↑ h; same h; ↓ h; stop working):<br>College +: 46.5; 36.3; 44.5; 32.8<br>12 or GED: 37.3; 34.4; 35.9; 36.8<br><12y: 14.4; 26.6; 18.1; 27.3<br>Unknown: 1.8; 2.8; 1.6; 3.0				
Dahdah et al. (2016)<br><br>USA<br><br>0.203 (43)<br><br><br>Level II	(1) To compare pt functional outcomes following TBI & to determine factors that influence those outcomes<br>(2) Prospective cohort study, secondary data analyses<br>(3) 19 TBIMS rehabilitation centers<br>(4) IC: pts aged ≥ 16 yrs, mod- sev TBI caused by blunt or penetrating Inj<br>EC: rehab AD occurring ≥30 d	(1) N=5505<br>(2) N.R<br>(3) Age: 42±20<br>(4) Sex: 72%<br>(5) TSI: 14±7d<br>(6) IS: mod-sev TBI<br>PTA duration (d): 22±18<br>(7) AT:<br>BL: DC<br>F/U: 1yr<br>(8) Education:<br>High school diploma or less: 60%	(1) Logistic regression model<br>(2) P≤0.01	(1) Functional status at AD, Sex, Marital status, Race, CT scan, Insurance, Geo unit quality score, LOS, Mechanism of TBI, Education, Employment, Alcohol or drug use, CCI & Duration of PTA<br>(2) N.R	(1) Functional outcomes<br>(2) DRS, FIM & GOS-E<br>(3) N.R	DRS DC & 1Yr: Longer duration PTA was SS predictor of outcome- Abnormal CT & Sex NS<br><br>FIM DC & 1Yr: Sex & Longer duration PTA SS predictors of outcome- Abnormal CT SS only at FIM 1 Yr<br><br>GOS-E 1Yr: Longer duration PTA SS predictor of outcome- Abnormal CT & Sex NS

	post-inj, inpt rehab course exceeding 90 d, died during inpt rehab, concomitant neurologic Dxs, rehab AD before 2003					
Forslund et al. (2017)<br><br>Norway<br><br>0.053 (6)<br><br>Level IV	(1) To assess longitudinal trajectories of overall Disability after mod-sev TBI & examine whether the trajectories could be predicted by sociodemographic & inj characteristics<br>(2) Case series<br>(3) Trauma center<br>(4) IC: 16-55 y.o. residence in eastern Norway, AD w/ ICD-10 Dx w/in 24h of inj, presence of mod-sev TBI w/ GCS 3-12 at AD or before intubation<br>EC: previous neurological D/Os/inj, assoc SCI, previously diagnosed sev psychiatric or substance abuse D/O, unknown address or incarceration	(1) N= 105<br>(2) Attrition: 11%<br>(3) Age: 30.9±11.2<br>(4) Sex: 78.1%<br>(5) TSI: Acute phase<br>(6) IS:<br>GCS<br>Mod (9-12): 32.4%<br>Sev (3-8): 67.6%<br><br>ISS<br>Score 0-29: 46.6%<br>Score 30+: 55.3%<br><br>PTA<br>0-18d: 59.7%<br>19+d: 40.3%<br><br>CT Head Marshall Score:<br>Score 1-2: 46.6%<br>Score 3+: 53.4%<br>(7) At:<br>BL: Acute phase<br>t_1: 1yr post-inj<br>t_2: 2 yrs post-inj<br>t_3: 5 yrs post-inj<br>(8) Education<br>≤12 yrs: 56.7%<br>>12 yrs: 43.3%	(1) HLM<br>(2) P<0.05	(1) Time, Sex, Age, Relationship status at AD, education, Employment status Pre-inj, Prior occupation, Acute GCS score, Cause of inj, PTA length, CT Head Marshall Score & ISS<br>(2) N.R	(1) Overall Disability<br>(2) GOS-E<br>(3) N.R	↑ GOS-E trajectories (↓ Disability) predicted by younger age at inj & shorter PTA & interaction of time*PTA – Sex, ISS, CT Head inj score NS

Kirkness et al. (2004)<br><br>USA<br><br>0.203 (43)<br><br>Level III	(1) To examine the interaction of sex & age in relation to outcome at 3 & 6mos post-inj in pts w TBI<br>(2) Prospective comparative study<br>(3) Level 1 trauma center<br>(4) IC: ≥16 y.o, AD to ICU at level 1 trauma center w/ Dx of TBI, & ICP & CPP monitoring<br>EC: bilateral fixed pupils, impending death	(1) N= 157<br>(2) 0%<br>(3) Age (M; W): 36.3±17.4; 39.9±20.7<br>(4) Sex: 79.0%<br>(5) TSI: 3 mos post-inj<br>(6) IS **(M;F)**:<br>ISS: 28.6 ±9.8; 30.1 ±9.1<br>AIS head score (=5): 31%; 52%<br>(7) AT:<br>BL: 3mos post-inj<br>F/U: 6 mos post-inj<br>(8) N.R	(1) Student's t test<br>Chi square test<br>Fisher's exact test<br>Mann-Whitney U test<br>MLRM<br>(2) P<0.05	(1) Sex, AIS, GCS & Age<br>(2) N.R	(1) Functional outcome<br>(2) GOS-E & FSE<br>(3) Mean Scores:<br>GOS-E 3mos (younger M; older M; younger F; older F): 3.75±1.6; 3.66 ±1.7; 3.88±1.5; 2.76 ±1.4<br><br>GOS-E 6mos (younger M; older M; younger F; older F): 4.83±2.1; 4.09±2.2; 4.56±1.8; 2.71±1.3)<br><br>FSE 3mos (younger M; older M; younger W; older F): 21.5±6.8; 21.8±7.4; 22.1±7.3; 26.6 ±4.1<br><br>FSE 6mos (younger M; older M; younger F; older W): 17.0 ±9.1; 19.9 ±9.4; 18.1±8.3; 26.2 ±4.3	GOSEGB-by-sex: SS predictors of outcome were AIS head*GCS-PR*Age & AIS head*GCS-PR-M* Age<br><br>GOSEGB-by-sex for subjects < 30: NS predictors of outcome<br><br>GOSEGB-by-sex for subjects ≥ 30 y.o: SS predictors include: Uncontrolled, AIS head*GCS-PR & AIS head*GCS-PR-M<br><br>Interaction of Sex & Age (Younger M, Older M, Younger F) SS predictors were: Uncontrolled, AIS head*GCS-PR & AIS head*GCS-PR-M

Leitgeb et al. (2011)<br><br>Austria<br><br>0.078 (14)<br><br>Level III	(1) To investigate sex diff in outcome after TBI<br>(2) Retrospective comparative study<br>(3) 17 medical centers<br>(4) IC: isolated TBI, survived at least until AD to ICU<br>EC: no additional inj with an AIS>2	(1) N= 439<br>(2) Attrition: 3.2%<br>(3) Age (M; W): 50.4±21.5; 61.4±22.6<br>(4) Sex: 69.5%<br>(5) TSI: 6mos post-inj<br>(6) IS (M;F):<br>AIS Head: 4.16±0.81; 4.21±0.93<br>ISS: 21.5±13.7; 23.2±17.9<br>GCS: 5.58± 2.83; 5.17±2.47<br>(7) AT:<br>BL: TOI (recorded by paramedics)<br>F/U: 6mos post-inj<br>(8) N.R	(1) Two-tailed t test<br>Fisher's exact test<br>Chi-square test<br>LRM<br>(2) P<0.05	(1) Age, Pupil abnormality, ISS, AIS Head, GCS, CT, Inj mechanism, Presence of Subdural Hematoma & Sex<br>(2) N.R	(1) ICU survival, Hospital survival & Favourable outcome<br>(2) Medical records, GOS & CT<br>(3) Frequency of Hospital Mortality (M;F): 32.5% (99/305) ; 39.6% (53/134)	Sex NS predictor of Mortality or Favorable outcome<br><br>Age & IS (ISS, AIS, CT) were SS associate wof Mortality & Favourable outcome
Rao et al. (2008)<br><br>USA<br><br>0.203 (43)<br><br>Level IV	(1) To assess the prevalence & risk factors for sleep disturbances in the acute post-TBI period<br>(2) Case series<br>(3) Trauma unit at Hopkins & the Brain Injury unit at Kernan Hospital, University of Maryland<br>(4) IC: first-time CHI w/in 3 mos of trauma, ability to provide consent personally, ≥18 y.o<br>EC: prior TBI, open-HI, Hx of Brain Illness	(1) N=54<br>(2) Attrition: 0%<br>(3) Age: 43.2±17.7<br>(4) Sex: 59.0%<br>(5) TSI: w/in 3 mons of trauma<br>(6) IS:<br>GCS 13-15 (mTBI): 65%<br>GCS 9-12 (mod TBI): 11%<br>GCS ≤8 (sev TBI): 19%<br>(7) AT:<br>BL: w/in 2 wks post-inj<br>F/U: 1-3 mos post-inj<br>(8) Education: 13.2±3.1	(1) Repeated measures ANOVA<br>MRM<br>(2) P< 0.05	(1) Age, Sex, MDGMC, GADGMC, GMHR, Body inj & GCS<br>(2) N.R	(1) Change in sleep domains<br>(2) MOS<br>(3) N.R	Sex SS predictive of SOBHA<br>Age SS predictive of Snoring & Daytime Sleepiness<br>No severity diff identified

Mollayeva et al. (2015)<br><br>Canada<br><br>0.098 (18)<br><br>Level IV	(1) To describe Community Integration & its assoc factors in insured Ontario workers w/ PCS<br>(2) Cross-sectional<br>(3) Rehab hospital<br>(4) IC: Workers w/ mTBI or concussion Dx<br>EC: w/o brain inj or inj due to electrocution, mod-sev TBI	(1) N= 94<br>(2) N/A<br>(3) Age: 45.2±9.94<br>(4) Sex: 62%<br>(5) TSI: 197 (416-139)d post-inj<br>(6) IS: mTBI<br>(7) N/A<br>(8) Education:<br>≤High school: 36%<br>High school-college, prof. diploma: 34%<br>≥University: 27%	(1) Spearman's correlation<br>Bivariate RM<br>MLRM<br>(2) P≤0.05	(1) Variables w/in: Sociodemographic, Clinical, Claim-related, Family-related, Social & Inj-related<br>(2) N.R	(1) Community Integration<br>(2) Medical records & CIQ<br>(3) CIQ Score (M; F): 13.96±4.67; 15.56±6.01	Sex & Age NS predictors of outcome
Ng et al. (2006)<br><br>Singapore<br><br>0.068 (11)<br><br>Level II	(1) To investigate if there are possible sex diff in relation to outcome following closed sev TBI in predominately Asian population<br>(2) Prospective cohort study<br>(3) Neurosurgical ICU<br>(4) IC: sev TBI, admitted btwn April 1999- December 2004<br>EC: N.R	(1) N= 672<br>(2) N/A<br>(3) Age **(M;F)**: 43.0±18.6; 53.7±22.8<br>(4) Sex: 78.1%<br>(5) TSI: 6mos post-inj<br>(6) IS: sev TBI<br>(7) N/A<br>(8) N.R	(1) Two-sample t-test<br>LRM<br>(2) P< 0.05	(1) Sex, Age, MI, Pupil abnormality & GCS score-PR<br>(2) N.R	(1) Mortality & Functional Outcome<br>(2) Medical records & GOS<br>(3) Frequency of Mortality (M;F): 73.75% (177/240); 26.25% (63/240)<br>Total: 35.7% (240/672)<br><br>Frequency of Poor Outcome (M;F): 70.9% (73/103); 29.1% (30/103)<br>Total: 15.3% (103/432)<br><br>Frequency of Good Outcome (M;F): 83.6% (275/329); 16.4% (54/329)<br>Total: 48.9% (329/ 432)	Sex NS predictor when variables (presence of multiple injuries, PR pupil abnormalities & GCS) were controlled for<br><br>F ≤ 60 y.o were SS more likely to have a poorer outcome even after controlling for variables<br>*unsure how much detail to go into about age, GCS

Ostberg et al. (2014)<br><br>Finland<br><br>0.056 (8)<br><br>Level IV	(1) To investigate whether smoking is assoc w/ outcome from TBI<br>(2) Cross sectional comparative study<br>(3) Neurological outpt clinic of university hospital<br>(4) IC: info about severity of inj (GCS or PTA), age ≥15 y.o<br>EC: uncertain TBI Dx, non-traumatic Neurological D/Os	(1) N= 689 (295=smokers; 392= non-smokers)<br>(2) N/A<br>(3) Age (at TBI): 39.8±14.9<br>Age (at survey): 49.1±15.1<br>(4) Sex: 65.3%<br>(5) TSI: 9.3± 8.4 yrs<br>(6) IS:<br>GCS-<br>Mild: 27.5%<br>Mod: 26.3%<br>Sev: 21.0%<br><br>PTA-<br>Mild: 37.5%<br>Mod: 26.3%<br>Sev: 23.8%<br>Very Sev: 12.4%<br>(7) N/A<br>(8) Education (yrs): 12.2±3.7	(1) T test<br>Chi-square test<br>Fischer's exact test<br>ANOVA<br>Kruskal-Wallis test<br>MLRM<br>(2) P< 0.05	(1) PTA, Age, Education, Smoking & Sex<br>(2) N.R	(1) Overall Functional outcome following TBI based on GOS-E (smokers vs non-smokers)<br>(2) Questionnaire & GOSE-E<br>(3) Frequency of Smokers (M;F): 75.9% (224/295); 24.1% (71/295)<br><br>Frequency of Non-smokers (M;F): 57.1% (224/392); 42.9% (168/ 392)	Age at TBI 41-55, 18-25 comparison SS predictor only for PTA sev cohort<br><br>Sex NS predictor at any PTA level
Ponsford et al. (2007)<br><br>Australia<br><br>0.120 (24)<br><br>Level III	(1) To compare 6mo survival & functional outcome in M vs W in a cohort of pts w/ sev TBI & Hypotension<br>(2) Retrospective comparative study; secondary analyses of RCT data<br>(3) 12 hospitals<br>(4) IC: Sustained blunt head trauma, any stage during prehospital care,	(1) N= 229<br>(2) N/A<br>(3) Age (M; F): 33.8±16.3; 43.3±23.1<br>(4) Sex: 65.9%<br>(5) TSI: 6mos post-inj<br>(6) IS: sev TBI<br>(7) N/A<br>(8) N.R	(1) Mann-Whitney U test<br>Student's t-test<br>MLRM<br>Post hoc Analyses<br>(2) P < 0.05	(1) Sex, Age, GCS at accident scene, Inj type, & Use of hypertonic saline<br>(2) N.R	(1) Survival & Functional outcome<br>(2) Medical records & GOS-E<br>(3) Pt Outcomes (M;F):<br>ED-<br>SBP (mmHg): 120 (95-140); 115 (84-130)<br>GCS: 3 (3-3); 3 (3-3)<br>Survival: 86%; 77%<br><br>ICU-<br>Serum sodium conc (mmol/L): 145±4.6; 146±5.8	After controlling for GCS, Age & Cause of injury, F SS ↓ rate of survival & good outcome at 6mos- appeared to reflect the ↓ rate of initial survival- those F surviving had similar outcomes to M<br><br>Sex SS effect on survival & good outcome

	GCS <9, Hypotension (SBP<100 mmHg), ≥18 y.o<br>EC: Penetrating trauma, pregnant, no intravenous access, serious premorbid Dx identified on a medical bracelet, peripheral oedema, in close proximity to the receiving hospital, absent sinus rhythm or cardiac arrest				GCS DC: 11 (10-14); 11 (10-14)<br>ICP: 13 (8-17); 10 (6-19)<br>CPP: 70±16; 74±17<br>↓ PaO_2/ FiO_2 ratio: 132 (73-250); 126 (68-200)<br>Inotropic support (d): 0.5 (0-3); 0.8 (0-3)<br>Mechanical ventilation (d): 4 (0.5-10); 4 (0.5-10)<br><br>Hospital DC-<br>LOS (d): 13 (1-26); 8 (0.5-20)<br>Survival: 59%; 39%<br>At 6mos-<br>GCS: 15 (15—15); 15 (15-15)<br>GOS-E: 3.4 ±2.4; 2.5±2.1<br>FIM: 110 ±27; 106±31<br>Return to work-<br>Same job: 16%; 3.8%<br>Modified: 14%; 12%<br>Not working: 70%; 85%	Sex-by-age only SS effect for >60
Renner et al. (2012)<br><br>Germany<br><br>0.066 (9)<br><br>Level III	(1) To examine the independent assoc of sex w/ IS, clinical course, pituitary dysfunction & outcome after TBI<br>(2) Retrospective comparative study<br>(3) 3 neurological rehab centers<br>(4) IC: mild-sev HI, 18–90 y.o	(1) N= 427<br>(2) N/A<br>(3) Age (W; M): 55.1±18.9; 46.4±18.6<br>18-39:20.4%; 38.2%<br>40-59: 34.3%; 34.5%<br>60-90: 45.4%; 27.3%<br>(4) Sex (W;F): 74.7%<br>(5) TSI: N.R<br>(6) IS (W; M):<br>Sev (GCS 3-8): 69.6%;<br>Mod (GCS 9-12): 13%; 13.6%	(1) Wilcoxon rank sum test<br>Fisher exact<br>Chi-square test<br>MLRM<br>(2) P<0.05	(1) Sex, Age, Severity, Pre-inj living situation & Employment<br>(2) N.R	(1) Functional, Rehab LOS, Pituitary Dz, Living situation & Employment status<br>(2) GOS & Clinical assessment<br>(3) Frequency of GOS (W; M):<br>GOS II: 10.4%; 5.6%<br>GOS III: 21.9%; 21.5%<br>GOS IV: 45.8%; 40.3%<br>GOS V: 21.9%; 32.6%	Sex NS predictive factor of course & outcome of TBI<br><br>Age SS predictor for employment but not for GOS or living situation<br><br>Severity SS for GOS & employment but not living situation

	EC: sev liver, heart & kidney Dx,<br>Prior HI, Stroke or sign substance and/or alcohol abuse, impending death	Mild (GCS 13-15): 17.4%; 19.1%<br>(7) N/A<br>(8) N.R			Unknown: 0% both<br><br>Rehab LOS in d (W; M)-<br>Mean±SD: 68.7 ±95.7; 64.6±64.3<br>Median: 45; 46<br><br>Pituitary insufficiency (F; M)-<br>Corticotroph deficiency: 3.7%; 3.1%<br>Gonadotroph deficiency: 0.9%; 14.1%<br>Thyreotroph deficiency: 6.5%; 3.5%<br>Somatotroph deficiency: 2.8%; 4.7%<br>Living situation (W; M)-<br>Lived alone: 7%; 6.8%<br>Lived w/ partner: 30%; 29.9%<br>Lived w/ care giver: 19%; 18.1%<br>Continuing inpt care: 44%; 45.2%<br>Unknown: 0% both<br>Employment status-<br>Employed: 15%; 26.6%<br>Unemployed: 1.7%; 2.9%<br>Student: 5%; 8.2%<br>Homemaker: 10%; 0%<br>Early retirement: 28.3%; 28.8%<br>Regular retirement: 40%; 33.5%<br>Unknown: 0% for both	

					*% as total numbers varied based on outcome variable	
Saban et al. (2011)<br><br>USA<br><br>0.203 (43)<br><br>Level III	(1) To compare perceived LS & perceived functional status 1 yr following sev TBI<br>(2) Retrospective comparative study<br>(3) TBIMS national multicenter longitudinal TBI outcomes database<br>(4) IC: 16-50 y.o, enrolled btwn yrs 1998-2008, diagnosed w/ sev TBI, LS data available at 1 yr post-inj, unconscious for at least 28d consecutively but regained consciousness w/in 1 yr	(1) N=287<br>(2) N/A<br>(3) Age (M; W): 27.06±8.97; 26.30±9.49<br>(4) Sex: 71.7%<br>(5) TSI: 1 yr post-inj<br>(6) IS: sev TBI<br>(7) N/A<br>(8) Education (M; W):<br>High school ≤: 58.5%; 41.0%<br>> high school: 25.5%; 38.6%	(1) t tests<br>Chi-square test<br>Multiple ordinary least squares RM<br>(2) P ≤ 0.05	(1) Sex, Age, Unconsciousness, Race, Family income, Education, Marital status, Cause of inj, ED SBP, Rehab efficiency, 1yr FIM motor, 1yr FIM cog<br>(2) N.R	(1) LS, functional outcome<br>(2) SWLS, FIM<br>(3) Mean Scores:<br>SWLS (M; F): 20.67±7.62; 18.49±7.71<br><br>FIM motor: (M; F): 4.33±0.315; 4.29±0.407<br><br>FIM cog (M; F): 3.34±0.237; 3.36±0.223<br><br>Total FIM (M; F): 4.66±0.251; 4.63±0.295	Sex did not SS influence perceived LS or motor function 1 yr after sev TBI<br>F had SS better cog outcomes compared to M 1 yr after sev TBI<br><br>Age & Unconscious was a SS predictive factor for SWLS 1 yr but not for motor or cog functioning
Takada et al. (2016)<br><br>Japan<br><br>0.116 (21)<br><br>Level IV	(1) To investigate Functional Ability & Social Participation of pts w/ TBI<br>(2) Qualitative study<br>(3) University-based level-1 trauma hospital<br>(4) IC: admitted to the trauma hospital	(1) N=12<br>(2) N/A<br>(3) Age at Inj/Assessment :<br>1. 19/22; 2. 36/38; 3. 61/65; 4. 39/45; 5. 37/39; 6. 22/23; 7. 27/32; 8. 30/32; 9. 30/34; 10. 33/39; 11. 27/31; 12. 38/42;<br>(4) Sex: 50%; (5) TSI:<br>1. ~3yrs post-inj<br>2. ~2 yrs post-inj	(1) N/A<br>(2) N/A	(1) N/A<br>(2) N.R	(1) Occupation status, Psychosocial troubles & Suitability of family support<br>(2) Interviews & Steps for Coding and Theorization method<br>(3) Frequency of Occupation Change: 75% (9/12)	The qualitative results indicated that social participation after<br>a TBI was influenced by the lack of support for each social stage, life Hx before TBI, post-inj cog function &

	for TBI, defined by ICD-10 classification, received rehab tx during acute phase of inj, 18-65 y.o, TBI sustained >1yr before time of study, Modified Rankin Scale score 0-3 & living in the community<br>EC: sev post-TBI aphasia, pre-existing Hx of cog impairment, psychiatric D/Os, mod physical impairments &/or living outside of the Kanagawa prefecture	3. ~4 yrs post-inj<br>4. ~6yrs post-inj<br>5. ~2yrs post-inj<br>6. ~1yr post-inj<br>7. ~5yrs post-inj<br>8. ~2yrs post-inj<br>9. ~4yrs post-inj<br>10. ~6yrs post-inj<br>11. ~4yrs post-inj<br>12. ~4yrs post-inj<br>(6) IS:<br>GCS:<br>1. 13; 2. 14; 3. 13; 4. 14; 5. 7; 6. 6; 7. 10;<br>8. 3; 9. 6; 10. 8; 11. 14; 12. 8<br>(7) N/A<br>(8) Education (yrs):<br>1. 15; 2. 14; 3. 14; 4. 12; 5. 12; 6. 9; 7. 14; 8. 12; 9. 14; 10. 16; 11. 16; 12. 9			Frequency of Psychosocial trouble: 42% (5/12)<br><br>Frequency of Suitability: 17% (2/12)	understanding & support from the family
De Koning et al. (2017)<br><br>Netherlands<br><br>0.044 (3)<br><br>Level II	(1) To study return to RTW after mTBI at several intervals after injury and to predict RTW on basis of occupational factors in addition to demographic, personality, & inj-related factors at 6 & 12 mos after inj<br>(2) Prospective cohort study<br>(3) 3 level 1 trauma centers	(1) N= 316 (nRTW=50; pRTW=53; cRTW=213)<br>(2) N.R<br>(3) Age (nRTW; pRTW; cRTW): 49.1±11.0; 44.6±13.8; 44.7±12.1<br>(4) Sex (nRTW; pRTW; cRTW): 66%; 47%; 69%<br>(5) TSI: 6mos<br>(6) IS:<br>ISS (nRTW; pRTW; cRTW): 12.2±9.0; 9.8±6.3; 6.7±4.4<br>CT abnormalities (nRTW; pRTW; cRTW): 23%; 25%; 12%	(1) Post-hoc analyses LRM<br>(2) P<0.05	(1) Age, Sex, Education level, MVA, ISS excluding head, GCS, Occupational category, Workload (h/wk), HISC (n of complaints), HADS Depression<br>(2) N.R	(1) RTW<br>(2) RTW Questionnaire<br>(3) Frequency of nRTW (M;F): 66% (33/50); 34% (17/50)<br><br>Frequency of pRTW (M;F): 47% (25/53); 53% (28/53)<br><br>Frequency of cRTW (M;F): 69% (147/213); 31% (66/213)	77% of pts had completely resumed work after 12mos<br><br>Sex, Age & Severity NS for 6mos or 12mos RTW

	(4) IC: pts w/ mTBI admitted to emerg<br>EC: previous TBI or psychiatric Dx requiring hospital AD, inability to be F/U, & substance abuse	GCS score (nRTW; pRTW; cRTW): 14.3±0.8; 14.4±0.6; 14.6±0.6<br>PTA (nRTW; pRTW; cRTW): 84%; 90%; 89%<br>LOC (nRTW; pRTW; cRTW): 80%; 85%; 82%<br>(7) AT:<br>BL: 6mos post-inj (for this study)<br>F/U: 12mos post-inj<br>(8) Median Education (nRTW; pRTW; cRTW): 5(2-7); 5(2-7); 5(2-7)				
van der Horn et al. (2013)<br><br>Netherlands<br><br>0.044 (3)<br><br>Level III	(1) To investigate the relation of Postconcussive complaints, Anxiety, & Depression w/ vocational outcome in pts w/ TBI<br>(2) Retrospective comparative study<br>(3) Level 1 trauma center<br>(4) IC: pts w/ TBI admitted to University Medical Centre Gronnigen<br>EC: <15 y.o, previous TBI	(1) N= 242 (51 Minor; 121 Mild; 70 Mod-Sev)<br>(2) N/A<br>(3) Age (Minor, Mild, Mod-Sev): 42.1±16.8; 40.3±17.3; 35.2±18.8<br>(4) Sex(Minor, Mild, Mod-Sev): 61%; 78%; 73%;<br>(5) N.R<br>(6) IS: 51 Minor; 121 Mild; 70 Mod-Sev<br>(7) N/A<br>(8) N.R	(1) Spearman correlation coefficient<br>Chi-square test<br>Binary LRM<br>MLRM<br>(2) P< 0.05	(1) GCS score, Postconcussive complaints, Anxiety & Depression<br>(2) N.R	(1) RTW<br>(2) RTW Questionnaire<br>(3) Frequency of RTW (Complete RTW reported*):<br>Minor (M;F): 82%; 50%<br>Mild (M;F): 72%; 55%<br>Mod-Sev (M;F): 44%; 47%<br>Total (M;F): 66%; 51%	(1) MLRM:<br>GCS: (OR=1.58; CI 1.17-2.13; P<0.01)<br><br>(2) MLRM: (only SS reported)<br>Postconcussive complaints:<br>(OR=8.45; CI 3.06-23.36; P<.001)<br>Anxiety: (OR=3.44; CI 1.36-8.74; P<0.01)<br>Depression:<br>(OR=3.28; CI 1.17-9.16; P<0.05)<br><br>(3) IS, Postconcussive complaints, Anxiety, & Depression were all SS predictive of

						RTW (explained variance 45%) when adj for Age & Sex
Vikane et al. (2016)<br><br>Norway<br><br>0.053 (6)<br><br>Level III	(1) To predict RTW at 12mos for pts who either were sick-listed or were at risk to be sick-listed w/ PCS at 6-8wks after inj<br>(2) Prospective comparative study; secondary analyses of RCT data<br>(3) 2 university hospitals<br>(4) IC: 16-55 y.o, hospitalized acutely at Department of Neurosurgery for mTBI, sick-listed or at risk to be sick-listed w/ persistent PCS<br>EC: current major Psychiatric Dx, head trauma, or other Dxs w impact on work; unemployment last 6mos, not Norwegian language, Dx w/ substance abuse in medical records, or lack of informed consent	(1) N= 151<br>(2) N/A<br>(3) Age: 32 (min=16, max=55)<br>(4) Sex: 61%<br>(5) TSI: 6-8wks post-inj<br>(6) IS: mTBI<br>(7) N/A<br>(8) Education (↑ education >13 years): 43%	(1) LRM<br>(2) P<0.05	(1) Age, Sex, Sick-listed in last yr, Intracranial inj, Postconcussion symptoms, PTSD, Anxiety, Depression, Expectation, Subjective health complaints, Widespread pain, Headache, Neck pain, Back pain, GOS-E, Sick-listed at 2mos<br>(2) N.R	(1) RTW<br>(2) RTW Questionnaire<br>(3) N.R	LRM (fully adj): Sex, Age, or Intracranial inj NS associate wof RTW

Gutman et al. (1995)<br><br>USA<br><br>0.203 (43)<br><br><br>Level IV	(1) To explore the disruption of gender identity & sex role as a result of TBI<br>(2) Case series<br>(3) Residential facility<br>(4) IC: be own legal guardian, sustained HI btwn ages 18-30, be at least 1 yr post inj, cog level high enough to understand & articulate interview questions<br>EC: N.R	(1) N=4<br>(2) N/A<br>(3) Age:<br>Pt 1: 46<br>Pt 2: 33<br>Pt 3: 36<br>Pt 4: 46<br>(4) Sex: 50%<br>(5) TSI:<br>Pt 1: 18 yrs post-inj<br>Pt 2: 12 yrs post-inj<br>Pt 3: 10 yrs post-inj<br>Pt 4: 16 yrs post-inj<br>(6) N.R<br>(7) N/A<br>(8) N.R	(1) Grounded theory<br>Constant comparative analysis<br>Triangulation<br>Negative case analysis<br>(2) N/A	(1) N/A<br>(2) Med Intake:<br>*Mentions Pt 3 & 4 maintained medical care post-inj	(1) Perceived masculinity/ femininity, Intimate relationships, Gender roles & Activities that support gender roles<br>(2) Interview<br>(3) N/A	M expressed ↑ feelings of sex inadequacy post-inj than F & ↑ diff resolving rites of passage & developmental issues characteristic of the life stage at which they experienced their inj<br><br>M depended more heavily on traditional sex-specific activities before & after inj to define and support sex role; F relied more on cross-sex activities<br><br>F able to maintain more pre-inj activities post-inj than M
Mollayeva et al. (2019)<br><br>Canada<br><br>0.203 (43)<br><br><br>Level IV	(1) To explore knowledge of sex and gender, and their influences in TBI recovery<br>(2) Case series<br>(3) Rehabilitation<br>(4) IC: adult men & women at acute and chronic phases of injury, mild or mod-	(1) N=40<br>(2) N/A<br>(3) Age: Acute phase- M: 48.6 ± 19.9; W: 42.9 ± 22.7; Chronic- M: 50.8 ± 14; 44.1 ± 16.8.<br>(4) Sex: 45%<br>(5) TSI: M : Acute phase (n=12); chronic phase (n=10); W : Acute phase (n=8), chronic phase (n=10)	(1) Applied thematic analyses; comparative analysis<br>Triangulation<br>Negative case analysis<br>(2) N/A	(1) Age, ethnicity, education, level of femininity/masculinity, TBI severity, marital status, impairments mostly cognitive/physical/ both<br>(2) N/R	(1) Knowledhe of sex and gender effects in TBI; understanding roles& responsibilities' change from pre- to post TBI<br>(2) Semi-structured interview<br>(3) N/A	W: Declarative knowledge better than in M<br><br>M & F: Procedural knowledge equal<br><br>Roles & responsibilities of M&F are gendered

	severe TBI, able to understand & articulate interview questions<br>EC: pts who did not sign informed consent	(6) M: mild (n=9); mod-severe (n=12); W: mild (n=11); mod-severe (n=7)<br>(7) N/A<br>(8) High school or less: M (n=6); W (n=4); college, university (bachelor degree): M (n=10); W (n=10), above bachelor degree				Pre-disability gendered roles & responsibilities disturbed in both M&F<br><br>New roles not formed yet; many roles and responsibilities taken on by significant others<br><br>M: feel demasculated<br>F: feel quilt<br><br>Both M & F dependent on others<br><br>Age, ethnicity, injury severity link to dependence level
Williamson et al (2016)<br><br>USA<br><br>0.203 (43)<br><br>Level III	(1) To investigate the influence of race, sex, functional ability & an array of pre-inj, inj-related & sociodemographic variables on LS over 10yrs following mod- sev TBI<br>(2) Retrospective comparative study	(1) N= 3157<br>(2) N.R<br>(3) Age: 35.87 ± 16.14<br>(4) Sex: 70.5%<br>(5) TSI: 1yr post-inj<br>(6) IS: mod-sev TBI<br>GCS: 8.97 ± 4.46<br>D of PTA: 23.89 ± 21.86<br>(7) AT:<br>BL: 1 yr post-inj<br>$t_{1:}$ 2 yrs post-inj<br>t_2: 5 yrs post-inj<br>t_3: 10 yrs post-inj<br>(8) Education: 12.83 ± 4.03 yrs	(1) HLM<br>(2) P ≤ 0.05	(1) Time, Sex, Race, FIM, PTA, GCS, Rehab LOS, Education, Earnings, WEPCE, Age & Relationship status<br>(2) N.R	(1) LS<br>(2) SWLS<br>(3) N.R	LS trajectories NS effected by sex, PTA duration, GCS or by the interaction of sex-by-time.<br><br>Age at inj was a SS predictor of outcome

	(3) 16 level 1 trauma centers, part of TBIMS database<br>(4) IC: mod-sev TBI classification based on intracranial neuroimaging, GCS <13, LOC > 30mins, PTA >24h, at least 16 y.o, AD w/in 72h of inj to emerg, consecutive receipt of acute care & inpt rehab services, informed consent<br>EC: GCS <13 due to intoxication, intubation, or sedation; LOC>30 mins due to intoxication or sedation					
Shonberger et al. (2009)<br><br>Australia<br><br>0.120 (24)<br><br>Level IV	(1) To investigate the assoc btwn age, IS, & brain lesion volumes, as well as viable brain volumes, following TBI<br>(2) Case series<br>(3) Hospital<br>(4) IC: sustainability for MRI scanning,	(1) N=98<br>(2) 1%?<br>(3) Age: 34.5±14.0<br>(4) Sex: 75.5%<br>(5) TSI: 2.3±1.5 yrs post-inj<br>(6) IS: sev TBI<br>(7) N/A<br>(8) Education: 12.4±2.5	(1) LRM MRM<br>(2) P< 0.05	(1) Sex, Accident type, Age, Time from inj to MRI & PTA<br>(2) N.R	(1) Viable Grey Matter & White Matter volumes<br>(2) MRI<br>(3) N.R	Older age was assoc w/ ↓ viable Grey Matter volumes in most neo-cortical brain regions (except Whole brain, Limbic & Brainstem) & NS for White Matter volumes<br><br>Longer PTA was assoc w/ ↓ viable

	absence of neurological D/Os other than TBI<br>EC: N.R					White Matter volumes in most brain regions (except Occipital & Cerebellum) & only SS in Sublobar region of Grey Matter<br><br>Sex was NS predictive factor of all regions of White matter & all regions of Grey matter
Wagner et al. (2004)<br><br>USA<br><br>0.203 (43)<br><br>Level III	(1) To determine if the relationship btwn CSF markers of excitoxicity, ischemia, & oxidative damage are sex & age specific & the role of hypothermia in affecting these relationships<br>(2) Retrospective comparative study<br>(3) Level 1 trauma center<br>(4) IC: For 48 h of mod<br>Hypothermia tx: 16-65 y.o, initial GCS score ≤8, (+) CT scan for TBI based on Marshall	(1) N= 68 (39 received hypothermia tx)<br>(2) N/A<br>(3) Age (M;F): 31.4; 31.7<br>(4) Sex (hypothermia; normothermia): 53.85%; 46.15%<br>(5) TSI: 1d post-inj<br>(6) IS:<br>GCS (M;F): 6; 5<br>(7) AT:<br>BL: 1d post-inj<br>$t_{1:}$ 2d post-inj<br>t_2: 3d post-inj<br>(8) N.R	(1) Levene's test<br>Logarithmic data transformations<br>Student's t test<br>Chi-square test<br>Fisher's exact test<br>Repeated measures multivariate model<br>(2) P<0.05	(1) Age, Time, Sex & Hypothermia<br>(2) N.R	(1) CSF Isoprostane/ Glutamate ratios & Isoprostane/Lactate/Pyruvate ratios<br>(2) Commercial enzyme immunoassay kit<br>(3) F2-isoprostane/glutamate ratios (M;F):<br>D1= 47.1±12.5; 42.2±19.3<br>D2: 63.9±20.4; 19.8±4.7<br>D3: 78.5±25.2; 11.4±3.5<br>F2-isoprostane/lactate/pyruvate ratios (M;F):<br>D1: 4.6±0.9; 3.3±0.7<br>D2: 2.7±0.7; 1.5±0.4<br>D3: 3.1±0.6; 0.8±0.1	SS sex effect & sex*time interaction on F2 isoprostane/glutamate ratios & / F2-isoprostane/lactate/pyruvate<br><br>Age SS ↑ isoprostane/glutamate ratios<br><br>F had a sign inverse relationship btwn D1 isoprostane/ glutamate ratios & GOS & isoprostane/lactate/pyruvate ratio & GOS scores

	classification method, placement of an external ventricular drain for standard of care ICP monitoring, signed consent from next-of-kin<br>IC: For 24 h of hypothermia tx: GCS score of 5-8, <46 y.o<br>IC: For CSF collection- admitted to level 1 trauma center w/ sev TBI<br>EC: N.R					
Wagner et al. (2007)<br><br>USA<br><br>0.203 (43)<br><br>Level III	(1) To determine assoc btwn factors that affect DAT expression & CSF DA & metabolite levels after sev TBI<br>(2) Retrospective comparative study<br>(3) Level 1 trauma center<br>(4) IC: For 48 h of mod Hypothermia tx: 16-70 y.o, initial GCS score ≤8, (+) CT scan for TBI, placement of an external ventricular drain for standard	(1) N=63 (28= hypothermia tx; 35= normothermia)<br>(2) Attrition: 23.1%<br>(3) Age **(M;F)**: 31.42± 2.04; 31.73 ±3.99<br>(4) Sex: 76.2%<br>(5) TSI: 1d post-inj<br>(6) IS: sev TBI<br>(7) AT:<br>BL: 1d post-inj<br>t_1: 2d post-inj<br>t_2: 3d post-inj<br>t_3: 4d post-inj<br>t_4: 5d post-inj<br>(8) N.R	(1) Student t test<br>Chi-square test<br>Fisher exact test<br>Logarithmic data transformation s<br>Univariate & multivariate mixed-effects model<br>(2) P≤0.05	(1) Age, GCS, Time, Sex, tx, group & Inotrope<br>(2) N.R	(1) CSF DA, DOPAC, & HVA levels<br>(2) Sterile port of external ventricular drain & Electrochemical detection methods<br>(3) N.R	Log (DA) SS predictors of outcome: Sex* Group; Sex, Age & GCS NS<br>Log (DOPAC) predictive factor of outcome: Sex; Age & GCS NS<br><br>Log (HAV) Age, Sex & GCS NS predictors of outcome<br>Log (DOPAC/DA) GCS SS predictor of outcome; Age & Sex NS

	of care ICP monitoring, signed consent from next-of-kin<br>IC: For 24 h of hypothermia tx: GCS score of 5-8, <46 y.o, CT studies (+) for TBI, placement of external ventricular drain for ICP monitoring					Log (HVA/DA) GCS predictive factor of outcome; Age & Sex NS
Wang et al. (2018)<br><br>China<br><br>0.152 (36)<br><br>Level IV	(1) To estimate the role of sex in intrinsic functional connectivity after acute TBI<br>(2) Case series<br>(3) ED at local hospital<br>(4) IC: mTBI pts w/ GCS 13-15, LOC <30 min, PTA ≤24h, other transient neurological abnormalities & intracranial lesion not requiring surgery, ≥18 y.o<br>EC: Hx of previous brain inj, neurological D/O, long-standing	(1) N=54 (27 M; 27 W)<br>(2) N/A<br>(3) Age (M;F) (35.4±9.7; 35.6±9.4)<br>(4) Sex : 50%<br>(5) N.R<br>(6) IS : mTBI<br>(7) N.A<br>(8) Education in yrs (M; W) : 9.4±3.5; 9.5±4.3	(1)Shapiro-Wilk test<br>2 sample t-test<br>Mann-Whitney test<br>Kruskal-Wallis test<br>Chi-square<br>ANOVA<br>(2) P<0.05	(1) Sex<br>(2) N.R	(1) Abnormal intrinsic functional connectivity within resting-state networks<br><br>(2) Clinical assessments-neuroimaging, neuropsychological tests (RPCS, DSC, VF, ISI)<br><br>(3) Score (M;F)<br>RPCS: 8.4±5.8; 11.4±6.4<br>DSC: 34.7±13.7; 36.7±14.1<br>VF: 17.1±4.8; 15.6±4.8<br>ISI: 5.9±4.5; 8.8±7.1	SS sex diff in motor, executive functional, ventral stream, cerebellum & visual networks

	psychiatric D/O, or concurrent substance or alcohol abuse, structural abnormality neuroimaging, intubation and/or presence of a skull fracture & sedatives, medication effect, other (psychological trauma, language barrier, or coexisting medical D/Os, caused by penetrating inj					
Albrecht et al. (2016)<br><br>USA<br><br>0.203 (43)<br><br>Level II	(1) To determine if there are sex diff in Mortality following isolated TBI in older adults & compare to findings using all TBI<br>(2) Retrospective cohort study<br>(3) Level 1 trauma center<br>(4) IC: ≥65 y.o, Dx of TBI<br>EC: N.R	(1) N= 1320 Isolated TBI; 1320 All TBI<br>(2) N/A<br>(3) Age of Isolated TBI(M;F):<br>65-74: 41%; 32%<br>75-84: 42%; 43%<br>>85: 18%; 25%<br>Age of All TBI: N.R<br>(4) Sex (Isolated TBI; All TBI): 55.3%; 58%<br>(5) N.R<br>(6) IS:<br>GCS All TBI (M;F)-<br>14-15: 68%; 71%<br>9-13: 14%; 14%	(1) Chi-square test<br>Student's t test<br>Stepwise RM<br>(2) $P < 0.05$	(1) Sex, Age, AIS head score, GCS, Race, Cardiac arrhythmia, AD SBP, Transport to trauma center, ISS, Severity of other inj, AIS face score, AIS spine score, AIS thorax score, AIS abdominal score, AIS upper extremity score<br>(2) N.R	(1) Mortality<br>(2) Medical records<br>(3) Frequency of Mortality of Isolated TBI pts at DC (M;F): 17% (123/730); 16% (96/590)<br><br>Frequency of Mortality for All TBI group: N.R	Sex (W) was not SS assoc w/ ↓ odds of mortality in Isolated TBI vs All TBI group, sex (F) was SS assoc w/ ↓ odd of mortality<br><br>Age was SS predictor of outcome in Isolated TBI & All TBI cases<br><br>AIS 3,4, ≥5 SS in Isolated TBI; AIS ≥5 only SS in All TBI

		3-8: 18%; 15%<br>GCS Isolated TBI (M;F):<br><9: 18%; 15%<br><br>AIS head (M;F)-<br>1: 20%; 20%<br>2: 2%; 4%<br>3: 10%; 13%<br>4: 42%; 38%<br>≥5: 27%; 25%<br>AIS head Isolated TBI (M;F):<br>3.5±1.4; 3.4±1.4<br>(7) N/A<br>(8) N.R				All GCS scores SS in both groups<br><br>ISS SS in All TBI group
Berry et al. (2009)<br><br>USA<br><br>0.203 (43)<br><br>Level III	(1) To determine whether sex or menopausal status affects mortality in pts w/ mod-sev TBI<br>(2) Retrospective comparative study<br>(3) National Trauma Data Bank<br>(4) IC: isolated mod-sev TBI<br>EC: ≤13 y.o, dead on arrival, dead w/in 24 h, transferred out to another facility, admitted w/ a burn Dx, head AIS>5, & had any missing data including age, sex, & head AIS	(1) N= 72, 294<br>(2) N/A<br>(3) Age: 44.6±21.8<br>14-20: 15.0%<br>21-45: 39.7%<br>46-55: 13.1%<br>56-65: 8.8%<br>66-75: 8.0%<br>76-89: 13.1%<br>>89: 2.3%<br>(4) Sex: 69.2%<br>(5) N.R<br>(6) IS:<br>GCS in ED: 11.1±4.9<br>ISS: 21.7 ±9.7<br>Head AIS: 3.8±0.7<br>(7) N/A<br>(8) N.R	(1) Wilcoxon's test<br>Chi-square test<br>LRM<br>(2) P<0.05	(1) Head AIS, Age group, Any complication, SBP, Inj mode, ISS, Comorbidity & Sex<br>(2) N.R	(1) Mortality<br>(2) Medical records<br>(3) Frequency of mortality (M;F): 9.2%; 9.4%	Head AIS, all age groups, sex & ISS were all SS predictors of outcome<br><br>F SS ↓ Mortality risk than M after adjust for confoundes<br>After age stratification, perimenopausal W (46–55 years) & postmenopausal W (≥ 55) showed a SS ↓ risk in mortality<br><br>No diff in Mortality in premenopausal F compared w/ their M

						age-matched counterparts
Davis et al. (2006)<br><br>USA<br><br>0.203 (43)<br><br>Level III	(1) To explore the relationship btwn sex & outcome in adult pts w/ mod-sev TBI using large trauma registry<br>(2) Retrospective comparative study<br>(3) Country wide trauma registry<br>(4) IC: admitted for at least 24h to 1/5 designated adult trauma centers<br>EC: head/neck AIS defined by a neck inj	(1) N= 13437<br>(2) N/A<br>(3) Age (N) **(M;F)**:<br>15-49: 7891; 1881<br>≥50: 2368; 1297<br>(4) Sex: 76.3%<br>(5) N.R<br>(6) IS: mod-sev TBI<br>(7) N/A<br>(8) N.R	(1) LRM<br>(2) P < 0.05	(1) Age, Sex, Comorbid Dx, Mechanism of inj Pre-AD GCS score, Presence of pre-AD hypotension (SBP≤ 90 mm Hg), Head AIS, & ISS<br>(2) N.R	(1) Survival to hospital DC<br>(2) Medical records<br>(3) Frequency of mortality (M;F):<br>15-19: 19.0%; 20.7%<br>20-29: 19.9%; 22.1%<br>30-39: 21.0%; 21.0%<br>40-49: 21.9%; 20.3%<br>50-59: 25.1%; 16.2%<br>60-69: 29.0%; 25.0%<br>≥70: 37.5%; 30.3%	NS diff in outcomes observed for pre-menopausal W (15-49) vs M but outcomes SS better in postmenopausal F (≥50) vs M after adj
Fortuna et al. (2008)<br><br>USA<br><br>0.203 (43)<br><br>Level III	(1) To determine whether preinjury CAW use was an important predictor of mortality in pts ≥50 y.o w/ blunt, HBI<br>(2) Retrospective comparative study<br>(3) University hospital<br>(4) IC: ≥50 y.o, Dx of blunt head trauma	(1) N=416<br>(2) N/A<br>(3) Age: 69 ± 1<br>(4) Sex: 61%<br>(5) N.R<br>(6) IS:<br>ISS: 24 ± 0.5<br>GCS: 12 ± 0.2<br>(7) N/A<br>(8) N.R	(1) Chi-square test<br>t test<br>ANOVA<br>Tukey's post hoc analysis<br>MLRM<br>(2) P <0.05	(1) Age, sex, ISS, GCS, CAW<br>(2) Medication Intake (N):<br>Clopidogrel- 17<br>Aspirin- 91<br>CLO/ASA- 18<br>Warafin- 29<br>ASA/ Warafin- 10<br>Heparin- 1<br>None- 250	(1) Mortality<br>(2) Medical records<br>(3) N.R	SS predictors of death included Age, ISS, & GCS<br><br>Sex NS predictive factor of mortality

	EC: no HBI					
Ottochian et al. (2009)<br><br>USA<br><br>0.203 (43)<br><br>Level III	(1) To examine outcomes between the sexes after TBI<br>(2) Retrospective cohort study<br>(3) Medical center<br>(4) IC: suffered blunt trauma, isolated sev TBI<br>EC: transferred to another acute care facility before 1wk, nonsurvivable head injuries (head AIS 6)	(1) N= 1807<br>(2) N/A<br>(3) Age (M;F): 43.0±20.7; 50.8±26.6<br>Age >55 y.o (M;F): 26.6%; 45.2%<br>(4) Sex: 77.5%<br>(5) N.R<br>(6) IS: sev isolated TBI<br>(7) N/A<br>(8) N.R	(1) Chi-square 2-sided Fisher exact test<br>Student t test<br>Kruskal-Wallis test<br>Mann-Whitney test<br>Binary logistic regression<br>Adj odd ratio<br>(2) P <0.05	(1) Sex, Age, AD SBP, AD GCS, ISS, Ethnicity & Toxicology<br>(2) N.R	(1) Mortality<br>(2) Medical records<br>(3) Frequency of Mortality (M;F): 29.5 (172/582) %; 41.5% (244/582)	F TBI pts had a SS ↑ mortality rate compared w/ their M counterparts<br><br>Age group SS assoc w/ the ↑ mortality rate was ≥55 y.o
Selassie et al. (2014)<br><br>USA<br><br>0.203 (43)<br><br>Level III	(1) To determine the influence of a number of factors on mortality after DC from acute care facilities in TBI pts<br>(2) Retrospective cohort study<br>(3) Medical center data set<br>(4) IC: cases with ICD-9-CM Dx codes: 800-801, 803-804, 850-854 & 959.01<br>EC: Pts coded as late effects of intracranial inj (907.0) & repeat	(1) N= 33695 (5413= Deceased; 28282= Alive)<br>(2) N/A<br>(3) Age (Total): 42.8<br>(4) Sex (Total): 64.1%<br>Deceased: 52.7%<br>Alive: 66.3%<br>(5) N.R<br>(6) IS % (Total; Deceased; Alive):<br>Severe: 34.7%; 45.9%; 32.5%<br>Moderate: 19.6%; 19.3%; 19.7%<br>Mild: 45.7%; 34.8%; 47.8%<br>(7) N/A<br>(8) N.R	(1) t-test<br>Chi-square test<br>HR<br>Multivariable Cox regression<br>(2) P < 0.001	(1) Comorbidities, TBI severity, Concomitant inj, Age, Sex, Race, Insurance status, Trauma facility level<br>(2) N.R	(1) Mortality<br>(2) Medical records<br>(3) Frequency of Mortality (M; W): 52.7% (2851/ 5413) ; 47.3% (2562/ 5413)	TBI severity, Age & Sex all SS associate of mortality after DC

	encounters with the<br>same Dx					
Boutin et al.<br>(2017)<br><br>Canada<br><br>0.098 (18)<br><br>Level II	(1) To evaluate RBCT frequency, determinants of transfusions & assoc clinical outcomes<br>(2) Retrospective cohort study<br>(3) Multiple trauma centers<br>(4) IC: Adult pts (aged ≥18 y.o) w/ a mod or<br>sev TBI defined using<br>ICD-10 S06 &<br>GCS) scores <13 or intubated on AD,<br>admitted to one of the centers participating to the registry btwn<br>April 2005-March 2013<br>EC: pts w un linked records to database	(1) N= 7062 (1991= RBCT)<br>(2) N/A<br>(3) Age (Total; RBCT):<br>18–55: 61.4%; 67.6%<br>56–65: 11.7%; 12.7%<br>66–75: 9.9%; 8.7%<br>≥75: 17.0%; 11.0%<br>(4) Sex (Total; RBCT): 73.2%; 68.7%<br>(5) N.R<br>(6) IS (Total; RBCT):<br>GCS-<br>Mod: 21.4%; 14.0%<br>Sev: 25.3%; 25.4%<br>Missing: 53.3%; 60.7%<br>AIS≥3 Head: 94.6%; 92.4%<br>(7) N/A<br>(8) N.R	(1) RR<br>HR<br>(2) P< 0.05	(1) Sex, Age, Comorbidities, Severity & Extracerebral injuries<br>(2) N.R	(1) RBCT & Outcomes according to transfusion status<br>(2) Medical records<br>(3) Frequency of RBCT (M;F): 68.7% (1368/1991); 31.3% (623/1991)	Sex (W), Age (15-65 ≥75), mod TBI all SS predictors of / RBCT<br>Mortality outcome: Age (18-55) & sev TBI (GCS) SS predictors<br><br>Complications outcome: Age (18-55, 56-65, 66-75, ≥75), mod & sev TBI (GCS) SS predictors<br><br>ICU outcome: Age (56-65, 66-75, ≥75), mod & sev TBI (GCS) SS predictors<br><br>Hospital stay outcome: Age (18-55, 56-65, 66-75, ≥75) mod & sev TBI (GCS) SS predictors<br><br>DC home: Age (18-55, 56-65), mod & sev TBI (GCS) SS predictors
Brown et al.<br>(2012)<br><br>Canada	(1) To investigate the diff in DC destination following acute care btwn M & W survivors of TBI<br>(2) Retrospective comparative study	(1) N= 3480<br>(2) N/A<br>(3) Median Age (M;F): 70-74; 75-79<br>(4) Sex: 45.4%	(1) Chi-square test<br>Mann-Whitney test	(1) Sex, Age, ISS, LOS, comorbidities, mechanism of inj<br>(2) N.R	(1) DC destination (care facility)<br>(2) Minimal Data Set of the Ontario Trauma Registry	Sex, Age & IS all SS associate wof DC destination

0.098 (18)<br><br>Level III	(3) Multiple acute care hospitals<br>(4) IC: >65 y.o, sustained a mod–sev TBI & were DC to a LTC facility, home w/ or w/o support services<br>EC: N.R	(5) N.R<br>(6) IS:<br>ISS (M;F): 9.4±6.7; 9.6±7.0<br>(7) N/A<br>(8) N.R	Binary logistic regression Multiple Logistic Regression<br>(2) P <0 .05		(3) DC Destination (M;F):<br>Home: 83% (1311/1581); 76% (1435/1899)<br>Away: 17% (270/1581); 24% (464/1899)	Following TBI, W were SS more likely than M to be sent to LTC facilities rather than home settings controlling for age, IS, mechanism of inj, & comorbidities
Côte et al. (2013)<br><br>Canada<br><br>0.098 (18)<br><br>Level IV	(1) To identify factors assoc w/ decisions to withdraw life-sustaining therapies in pts w/ sev TBI<br>(2) Case series<br>(3) 6 level 1 trauma centers<br>(4) IC: mechanically ventilated pts,<br>≥16 y.o, admitted to an ICU following a sev blunt TBI documented in emerg or at ICU AD<br>EC: N.R	(1) N= 720 (228=non-survivors)<br>Non-survivors: Withdrawal of life-sustaining therapies= 160<br>No withdrawal= 68<br>(2) N/A<br>(3) Age >55 (Withdrawal, No Withdrawal): 55.0%; 33.8%<br>Mean age: 50.7±21.7<br>(4) Sex (Withdrawal, No Withdrawal): 68.8%; 73.5%<br>(5) N.R<br>(6) IS: sev TBI<br>(7) N/A<br>(8) N.R	(1) MLRM<br>Pearson chi-square test<br>(2) P < 0.05	(1) Sex, Age, Clinical factors, CT scan & Interventions<br>(2) N.R	(1) Factors assoc w/ decision to withdraw life-sustaining therapies<br>(2) Medical records<br>(3) Frequency of Withdrawal of Life-Sustaining Therapies (M;F): 68.8% (88/160); 31.2% (72/180)<br><br>Frequency of No Withdrawal (M;F): 33.8% (23/68); 66.2% (45/68)	Sex & Age not SS predictors of decision to withdrawal life-sustaining therapies<br><br>CT scan Epidural Hematoma/hemorrhage & Herniation SS predictors of outcome
Hoffman et al. (2012)<br><br>USA	(1) To evaluate the impact of Medicare's inpt rehab facility PPS on use of rehab for individuals w/ TBI	(1) N= 135842 (71561=Pre-PPS; 64281=Post-PPS)<br>(2) N/A<br>(3) Age (Pre-; Post-	(1) t test<br>Mann-Whitney U test<br>MLRM<br>(2) P < 0.05	(1) Age, Sex, Race, Cause of injury, GCS, AIS head, ISS nonhead, Insurance, Facility & PPS status	(1) DC location<br>(2) Medical records<br>(3) N.R	Age, Sex & IS all SS associate wof outcome

0.203 (43)<br><br>Level III	(2) Retrospective comparative study<br>(3) 123 level I and II trauma centers<br>(4) IC: AIS head score of ≥2<br>EC: N.R	PPS): 35.0±22.5; 37.0±23.5<br>(4) Sex (Pre-; Post-PPS): 70.0%; 69.8%<br>(5) N.R<br>(6) IS (Pre-; Post-PPS):<br>GCS: 12.7±3.8; 13.2±3.4<br>AIS head: 3.21±0.94; 3.23±0.93<br>(7) N/A<br>(8) N.R		(2) N.R		After controlling for pt characteristics, inj characteristics, insurance type, & facility, odds of being DC to an Inpt Rehab facility after TBI ↓ 16% after Medicare's PPS system was enacted
Kisat et al. (2012)<br><br>USA<br><br>0.203 (43)<br><br>Level III	(1) To determine pt & inj characteristics that predict a (+) CT scan or need for a Neurosurgical Procedure in pts w/ blunt head inj & a normal GCS<br>(2) Retrospective comparative study<br>(3) National Trauma Data Bank<br>(4) IC: ≥16 y.o, presented to ED w/ minor HI<br>EC: missing head CT scan results	(1) N= 83566 Total Pop<br>(59153= (-) CT scan; 24424= (+) CT scan)<br>(3476= Underwent Neurosurgical Procedure; 80090= No Procedure)<br>(2) N/A<br>(3) Age (Total Pop):<br>16-25: 26.7%<br>26-35: 16.2%<br>36-45: 16.1%<br>46-55: 13.6%<br>56-65: 8.4%<br>66-75: 6.6%<br>76-84: 6.8%<br>≥85: 2.9%	(1) Student's t test<br>Chi-square test<br>Univariate analysis<br>MLRM<br>(2) P < 0.05	(1) Age, Sex, Hypotension, Race, Insurance coverage, Mechanism of inj & CT scan<br>(2) N.R	(1) (+) head CT scan & Neurosurgical Procedure<br>(2) Medical records<br>(3) Frequency of (+) CT scan (M;F): 66.5% (16232/ 24414); 33.5%(8182/24414)<br><br>Frequency of Neurosurgical Procedure (M;F): 72.3% 2514/3476; 27.7% 937/3476	Older pts (≥36) more likely to have a (+) finding on a head CT scan – Sex not SS factor<br><br>Sex (M), Age (46-55, 56-65, 76-84) & (+) CT scan SS predictors Neurosurgical Procedure

		(4) Sex (Total Pop): 66.7%<br>(5) TSI: ED, N.R<br>(6) IS (Total Pop): AIS-<br>1: 19.9%<br>2: 44.6%<br>3: 19.2%<br>4: 14.2%<br>5: 2.2%<br>(7) N/A<br>(8) N.R				

Li et al. (2018)<br><br>USA<br><br>0.203 (43)<br><br>Level II	(1) To investigate the effects of age & sex on 30-, 60-, & 90d hospital readmission after acute hospital DC for individuals w/ TBI<br>(2) Retrospective cohort study<br>(3) Nationwide Readmissions Database<br>(4) IC: pts w/ TBI based on the ICD codes (800.XX, 802.XX, 803.XX, 804.XX, 851.XX-854.XX, >18 y.o, EC: ICD code 850.XX (Concussion), those who dies during index hospitalization	(1) N= 52, 877 (30d Readmission=52877; 60d Readmission=48300; 90d Readmission=43408)<br>(2) N/A<br>(3) Age (30d; 60d; 90d Readmission):<br>18-40: 21.5%; 21.6%; 21.7%<br>41-65: 27.5%; 27.5%; 27.7%<br>66-75: 14.1%; 14.0%; 14.0%<br>≥76: 37.0%; 36.8%; 36.7%<br>(4) Sex (30d; 60d; 90d Readmission): 59.1%; 59.3%; 59.3%<br>(5) N.R<br>(6) IS (30d; 60d; 90d Readmission): DRG-<br>Minor: 26.9%; 27.0%; 27.2%<br>Mod: 42.3%; 42.3%; 42.1%<br>Major: 23.5%; 23.5%; 23.5%<br>Extreme: 7.3%; 7.35; 7.2%<br>(7) N/A<br>(8) N.R	(1) Multivariate logistic regression<br>(2) P ≤0.05	(1) Age, Sex, Severity, DC Destination & Teaching status<br>(2) N.R	(1) Hospital Readmission<br>(2) Medical records<br>(3) Frequency of Hospital Readmissions (age-by-sex weekly):<br>M (1st; 2nd; 3rd; 4th; 5th week)<br>18-40: 34.92; 17.01; 7.56; 6.76; 5.45<br>41-65: 27.56; 15.78; 11.81; 8.20; 6.53<br>66-75: 29.91; 15.29; 10.46; 7.75; 6.49<br>≥76: 29.02; 16.67; 9.48; 8.41; 6.22<br><br>W (1st; 2nd; 3rd; 4th; 5th week)<br>18-40: 35.15; 14.05; 7.22; 6.79; 5.98<br>41-65: 26.57; 17.88; 9.59; 8.80; 7.11<br>66-75: 26.00; 18.14; 13.35; 6.27; 4.98<br>≥76: 27.12; 15.80; 10.31; 8.16; 6.61	Sex diff in 30-, 60-, & 90d hospital readmission were found in all age groups- all SS predictors of outcome<br><br>The largest sex diff in hospital readmission were in the 2 oldest groups (66-75 & ≥76)<br><br>For both sexes, the oldest group (≥76) had the highest adj 90d readmission risk for M<br><br>In those readmitted w/in 90d, the youngest group (18-40) had the ↑ cumulative readmission % (35% for both sexes) w/in the 1st week of hospital DC

Rush et al. (2016)<br><br>USA<br><br>0.203 (43)<br><br>Level III	(1) To characterize the surgical management of ASDH & to determine the independent assoc of DC for ASDH compared to Craniotomy<br>(2) Retrospective comparative study<br>(3) Nationwide inpt sample database<br>(4) IC: ≥18 y.o, primary Dx of traumatic Subdural Hemorrhage (ICD9 codes 852.20–852.29) from the 2006–2011 NIS sample, underwent Craniotomy or Craniectomy<br>EC: pts w/ codes for both surgeries	(1) N= 302 propensity matched cohort (151=Craniotomy; 151= Craniectomy)<br>(2) N/A<br>(3) Age (Craniotomy; Craniectomy): 52.73±21.5; 52.37±20.2<br>(4) Sex (Craniotomy; Craniectomy): 69.9%; 71.7%<br>(5) N.R<br>(6) N.R<br>(7) N/A<br>(8) N.R	(1) Chi-square test<br>Independent t test<br>MLRM<br>(2) P < 0.05	(1) Age, Time, Sex, Zip code Income, Race, Hospital teaching status, Hospital location, Hospital size, Hospital region, Comorbidities & APRDRG severity index<br>(2) N.R	(1) Undergoing a Craniectomy<br>(2) Medical records<br>(3) Frequency of Craniotomy (M;F): 69.9% (106/151); 30.1% (45/151)<br><br>Frequency of Craniectomy (M;F): 71.7% (108/151); 28.3% (43/151)	In the USA in pts w/ ASDH, craniotomy is performed 10x more often than decompressive craniectomy<br><br>Age & APDRG Severity Index SS associate w undergoing a Craniectomy<br>Sex NS predictor of outcome

Abbreviations: AD, admission; adj, adjusted; AIS, Abbreviated Injury Scale; ANKK1, Ankyrin repeat and kinase domain containing 1; ANOVA; analysis of variance; AP, arterial pressure; APOE, apolipoprotein E; APRDRG, All Pts Revised Diagnosis Related Groups; ASA, aspirin; ASDS, Acute Stress D/O Scale; ASDS-Diss, Acute Stres D/O Scale-Dissociative Score; assoc, association; AT: assessment time points; AUC, area under curve; AUD, Alcohol Use D/O; BAI, Beck's anxiety inventory; BDI-II, Beck Depression Inventory II; BCVI, Blunt cerebrovascular injury; BL, baseline; btwn, between; BP, blood pressure; CAW, clopidogrel, aspirin,or warfarin; CCI, Charlson Comorbidity Index; CDR, Clinical Dementia Rating; CES-D, Center for Epidemiological Studies-Depression; CHART-SF, Craig Handicap Assessment and Reporting Technique–Short Form; CHI, closed head injury; CI, confidence interval, CIQ, Community Integration Questionnaire; CLO, Clopidogrel; CLTR, Consistent Longer Term Retrieval Score; CNS, central nervous system; cog, cognition; COMT, catechol-o-methyltransferase; conc, concentration; COWAT, Controlled oral word association test; CPP, cerebral perfusion pressure; cRTW, complete return to work; CT, computed tomography; CSF, cerebrospinal fluid; CVLT, California Verbal Learning Test; d, day; DA, dopamine; DAT, DA transporter; DC, discharge; DOPAC, dihydroxyphenylacetic acid; diff, differences; DRD2, Dopamine Receptor D2; DRG, Diagnosis Related Groups; D/O, disorder; DSC, WAIS- III Digit Symbol Coding Score; DSM, Diagnostic and Statistical Manual of Mental D/Os; DST, digit span test; Dx, D/O/Diagnosis; D-KEFS, Delis–Kaplan Executive Function System; EC, exclusion criteria; ED, emergency department; est, estimate; ESS, Epworth Sleepiness Scale; F, female; FIM, Functional Independence Measure; FiO_2, oxygen inspired fraction; FSE, functional status examination; FSIQ, Full Scale Intelligence Quotient; F/U, follow-up; GADGMC, Generalized Anxiety D/O due to General Medical D/O; GCS, Glasgow Coma Scale; GED, General Equivalency Diploma; GOSEGB; Glasgow Outcome Scale Extended Good/Bad outcome; GOS-E, Glasgow Outcome Scale- Extended; gov, government; GMHR,

General Medical Health Rating; GRS; gene risk score; GSW, gunshot wounds; h, hour; HADS, Hospital Anxiety and Depression Scale; Hb, hemoglobin; HBI, hemorrhagic brain injury; hetero, heterozygous; HI, head injury; HISC, Head Injury Symptom Checklist; Hg, Mercury; HLM, Hierarchical linear modelling; HMO, Health Maintenance Organization; homo, homozygous; HR, hazard regression; HSQ, Health Status Questionnaire; HVA, homovanillic acid; Hx, history; IC, inclusion criteria; ICF, intermediate care facility; ICD, International Statistical Classification of D/Os and Related Health Problems; ICP, intracranial pressure; ICU, intensive care unit; inj, injury; IS, injury severity; ISI; Insomnia Severity Index; ISS, injury severity score; IQ, intelligence quotient; Junior-ES, junior elderly subjects; LOC, loss of consciousness; LOS, length of stay; LRM, Logistic regression model; LS, life satisfaction; LTC, long term care; M, man/male; MANOVA, multiple analysis of variance; MANCOVA, Multivariate Analysis of Covariance; MDGMC, Major Depressive-like Episode due to General medical D/O; medication, med; MHI, mild head injury; MI, multiple injuries; min., minimum; MINI, Mini International Neuropsychiatric Inventory; mins, minutes; MLRM, Multivariate Logistic/Linear Regression Model; mm, millimeter; mmol; millimole; MMSE, mini-mental status examination; mod, moderate; mos, months; MRI, magnetic resonance imaging; MRM, Multivariate/multivariable regression model; MSI, multisensory impairment; mTBI, mild traumatic brain injury; MVA, motor vehicle accident; NFI, Neurobehavioral Functioning Inventory; nRTW, no return to work; NS, not significant; N.R, not reported; N/A, not applicable; OR, Odds Ratio; PaO_2, oxygen arterial pressure; PATH, Patient and Total Health; $PbrO_2$, Brain tissue oxygen pressure; PCS, Postconcussion Syndrome; PCSC, Postconcussion Syndrome Checklist; PHQ, Patient Health Questionnaire; pt, patient; PIQ, Performance Intelligence Quotient; pop, population; post-inj, post-injury; PPS, prospective payment system; PR, postresuscitation; pRTW, partial return to work; PR-M, postresuscitation-motor; PSG, Polysomnography; PTA, post-traumatic amnesia; PTCI, Posttraumatic cerebral infarction; PTSD, Post-Traumatic Stress D/O; pre-inj; pre-injury; pts, pts; P-VAS, Pain Visual Analogue Scale; RBCT, red blood cell transfusion; RDI, Respiratory disturbance index; ref, reference; REM, rapid eye movement; rehab, rehabilitation; retro, retrospective; RLAS, Ranchos Los Amigos Levels of Cognitive Functioning Scale; RM, Regression Model; ROCF, Rey–Osterrieth complex figure test; RPCS, Rivermead Post Concussion Symptom Questionnaire; RR, risk ratio; RTS, Revised trauma score; RTW, return to work; SBP, systolic blood pressure; SCI, spinal cord injury; SCZ, schizophrenia; SD, standard deviation; SDMT, Symbol Digit Modalities Test; SES, socioeconomic status; Senior-ES, senior elderly subjects; sev, severe; SEQRT1, Sequential Reaction Time Test; sign, significant; SIP, Symptom impact profile; SIP-AB, Symptom impact profile- alertness behavior; SNF, skilled nursing facility; SNSB, Seoul Neuropsychological Screening Battery; SOBHA, Shortness of Breath/ Headache; SS, statistically significant; SSRI, Selective serotonin reuptake inhibitor; STW. Spot-the-Word test; SUB, Substance Use D/O; SWLS, Satisfaction with life scale; Sx, symptom; TBI, traumatic brain injury; TBIMS, Traumatic Brain Injury Model Systems; TMT, trail making test; TOI, time of injury; TRISS, Trauma Injury Severity Score; TSI, time since injury; tx, treatment; VA-DOD; Veteran Affairs- Department of Defense; VF, Verbal Fluency Test; VIQ, Verbal Intelligence Quotient; VMAT, vesicular monoamine transporter; vs, versus; WAIS, Wechsler Adult Intelligence Scale; WEPCE, weeks in paid competitive employment; wks, weeks; WMS, Wechsler Memory Scale; w/, with; w/in, within; w/o, without; yrs, years; y.o, years old

Supplementary Table 5: Summary of study characteristics, samples, and results pertaining to sex/gender and outcomes.

Study Outcome-<br>Sex or gender?	Age (y)/<br>Stage, time since injury, and time of post-injury assessment	Analysis controls for PROGRESS or related variables	Sex/gender related results
Stroke[53]<br>Sex/gender	Mean (SD): 46.1 (20.1)/<br>-Chronic<br>-Mean (SD) time of assessment: 1.94 (1.18) y	-Urbanization<br>-Socioeconomic status	Male persons have a higher risk of stroke after TBI.
PCS[61]<br>Sex/gender	Mean (SD):<br>34.7 (13.7)/<br>-Acute<br>-Mean (SD):<br>4.9 (2.63) d	-None	Female persons more likely to experience PCS.
Alcohol use disorder (AUD)[67]<br>Sex/gender	Mean (SD):<br>29.75 (7.71)/<br>NR	None	<u>Male veterans</u><br>-Positively associated with AUD: PTSD<br>-Negatively associated with AUD: Age<br><u>Female veterans</u><br>None
Insomnia[63]<br>Potentially sex	Mean (SD):<br>45.2 (9.9)/<br>-Chronic<br>-Median (IQR) time since injury:<br>197 (139 – 416) d	-Education<br>-English as a first language<br>-Weekly income<br>-Working status<br>-Having close friends<br>-Family difficulties<br>-Marital status<br>-Children in household	Sex not associated with insomnia frequency and severity.
Chronic pain[64]<br>Potentially sex	Mean (SD):<br>45.2 (9.94)/<br>-Chronic<br>-Median (IQR) time since injury:<br>197 (139 – 416) d	-Education<br>-English as a first language<br>-Working status<br>-Working > 40 hours/week	<u>Male persons and Female persons combined</u><br>Sex is not associated with pain.<br><br>Differing higher pain scores' associations based on sex

Sleep architecture deviations from normative data[65]<br><br>Sex/gender	Mean (SD): 47.5 (11.3)/<br>-Chronic<br>-Median (IQR) time since injury: 799 (135-9008) d	-Education	Sex/gender not associated with deviations in sleep stage distribution.
Multisensory impairment[54]<br><br>Sex/gender	Mean (SD):<br>30.6 (8.2)/<br>NR	-Branch of service<br>-Rank	Veteran Female persons more likely to report symptoms of multisensory impairments.
Red blood cell transfusion[104]<br><br>Sex/gender	-Acute<br>-NR (during hospital stay)	None	Female persons more likely to have red blood cell transfusion.
Pneumonia[80]<br><br>Sex/gender	Mean (SD):<br><u>Male persons</u><br>44.8 (21.6)<br><u>Female persons</u><br>40.7 (17.3)/<br>-Acute<br>-NR (during hospital stay)	None	Factors associated with pneumonia are the same in Male persons and Female persons, with the exception of advanced age, only significant in Male persons.
Cerebral oxygenation after red blood cell transfusion[73]<br><br>Sex/gender	Mean (SD):<br><u>Entire sample</u><br>35.4 (15.9)<br><u>Male persons</u><br>32.8 (13.9)<br><u>Female persons</u><br>45.5 (19.4)/<br>-Acute<br>-NR (during hospital stay)	None	Female persons exhibit greater cerebral oxygenation in response to red blood cell transfusion.
Cognition[58]<br><br>Potentially sex	Age group frequency<br>-16-24 (27%)<br>-25-34 (18.6%)<br>-35-44 (18.8%)<br>-45-54 (12.3%)<br>-55-64 (7.1%)<br>-65+ (16.2%)/	-Relationship status<br>-Ethnicity<br>-Governmental funding<br>-Employment at injury	Female persons have worse outcomes with respect to cognitive symptomology

	-Chronic<br>-1 y		
Craniotomy vs. craniectomy[77]<br><br>Sex/gender	Mean (SD):<br>-68.9 (17.1) for craniotomy<br>-49.5 (20.8) for craniectomy/<br>-Acute<br>- < 1 d (during hospital stay)	-Race<br>-Insurance status<br>-Zip code income quartile<br>-Urban vs. rural hospital status	Sex/gender not associated with propensity score of receiving craniectomy vs. craniotomy.
Cerebral infarction[74]<br><br>Sex/gender	-Acute<br>-During hospital stay<br>-Mean (range) length of stay:<br>18 (2 – 124) d	None	Sex/gender not associated with incidence of post-traumatic cerebral infarction.
Hospital readmission[106]<br><br>Sex/gender	Frequency of age groups:<br><u>30 d readmission</u><br>-18-40 (21.5%)<br>-41-65 (27.5%)<br>-66-75 (14.1%)<br>- ≥76 (37%)<br><u>60 d readmission</u><br>-18-40 (21.6%)<br>-41-65 (27.5%)<br>-66-75 (14%)<br>- ≥76 (36.8%)<br><u>90 d readmission</u><br>-18-40 (21.7%)<br>-41-65 (27.7%)<br>-66-75 (14%)<br>- ≥76 (36.7%)/<br>-Acute<br>-30 – 90 d	-Primary payer<br>-Country of residence by urban status<br>-Country population<br>-Zip code income quartile	Older groups are more likely to be readmitted to hospitals 30-, 60-, and 90-days post-injury.<br><br>No adjusted comparison of odds for Male persons relative to Female persons.
Symptom impact[92]<br><br>Sex/gender	Mean (SD):<br>40.6 (19.9)<br>-Chronic<br>-1 y	-Race<br>-Employment status at injury<br>-Funder	Female persons have worse outcomes with respect to cognitive symptomology.

Sleep disturbances, daytime sleepiness, and sleep index[86]<br><br>Sex/gender	Mean (SD):<br>43.2 (17.7)/<br>-Acute<br>-Trauma unit	None	Female sex associated with shortness of breath and headache on univariate analysis, but not multivariate.
Pituitary function[94]<br><br>Sex/gender	Mean (SD):<br>Female persons<br>55.1 (18.9)<br>Male persons<br>46.4 (18.6)/<br>-Acute<br>-12 w	None	Sex/gender not associated with pituitary insufficiency.
Brain lesions[107]<br><br>Sex/gender	Frequency of age groups (at injury):<br>-17-30 (54.1%)<br>-30-40 (12.2%)<br>-40-50 (14.3%)<br>-50-60 (13.3%)<br>-60-73.3 (6.1%)/<br>-Chronic<br>-Mean (SD) time since injury:<br>2.3 (1.5) y<br>-Median (IQR) time since injury:<br>2.2 (0.3-5.7) y	None	Sex/gender not associated with white and gray matter lesion volumes.<br><br>Male persons have larger volumes of viable gray and white matter.
DeMale personstia[83]<br><br>Sex/gender	Median (range):<br>44.00 (27.00-64.00)/<br>-Acute<br>-Median (IQR) follow up time: 52 months	-Income level	Male persons have a greater relative risk of dementia.<br><br>Female persons develop dementia earlier after an injury.
Alcohol use disorder (AUD)[67]<br><br>Sex/gender	Mean (SD):<br>29.75 (7.71)/<br>NR	None	In male veterans, AUD diagnosis was associated with:<br>-Greater PTSD severity<br>-Younger age<br>-mTBI not predictive

			In female veterans, none of the assessed factors distinguished between those with an alcohol use disorder diagnosis and those without.
Schizophrenia[82]<br><br>Non-affective psychosis<br><br>Sex/gender	Frequency of age groups (at diagnosis):<br>Schizophrenia<br>-16-20 (36.8%)<br>-21-25 (51.6%)<br>-26+ (11.6%)<br>Non-affective psychosis<br>-16-20 (36.8%)<br>-21-25 (52%)<br>-26+ (11.2%)/<br>-Chronic<br>- < 3 y	-Childhood SES<br>-Area of birth (urban, rural, suburban)	In both sexes, severe head injury is associated with subsequent non-affective psychoses, but not subsequent schizophrenia.
Depression[81]<br><br>Sex/gender	Mean (range, SD):<br>43.6 (18.2 – 74.8, 15.2)/<br>-Chronic<br>-Mean time since injury (range, SD):<br>41.4 (4.3 – 260.2, 58.9) m	None	Female persons more likely to experience depression across severities, as indicated by a BDI-II score > 13.<br><br>No difference between the sexes for moderate and severe depression, as indicated by a BDI-II score > 19.
Return to work (RTW) between 6 and 12 months[60]<br><br>Potentially sex	Mean (SD):<br>-49.1 (11.0) under no RTW<br>-44.6 (13.8) under partial RTW<br>-44.7 (12.1) under complete RTW/<br>-Chronic<br>-Time since injury:<br>6 m or 12 m	-Education<br>-Occupational category<br>-Workload	Sex/gender not associated with RTW.
Community integration[62]<br><br>Potentially sex	Mean (SD):<br>45.2 (9.9)/<br>-Chronic<br>-Median<br>(IQR) time since injury: 197 (139 – 416) d	-Education<br>-Marital status<br>-English as first language	Sex/gender not associated with community integration
Disability[66]<br><br>Sex/gender	Mean (SD):<br>45.1 (9.9) y/<br>-Chronic	-Work status	Sex/gender not associated with work, social, family/home life, or global disability.

	-Median (IQR) time since injury: 196 (138 – 412) d		
Cognitive functioning[70]<br><br>Sex/gender	-Chronic<br>-Mean (SD) time since injury:<br><u>Male persons</u><br>22.37 (20.42) m<br><u>Female persons</u><br>25.39 (23.88) m	-Education	Sex/gender, and sex/gender and time since injury not associated with performance on the neuro-psychological test measures.<br><br>Sex/gender and age associated with performance:<br>-Category test<br>-Trail making test<br><br>Females ≤ 30 performed better than males of the same age, but males ≥ 30 performed better.
Return to work (RTW)[68]<br><br>Sex/gender	Median (range): 32 (16 – 55)/<br>-Chronic<br>-12 m	None	Sex/gender not associated with RTW.
Work-related disability[85]<br><br>Sex/gender	Mean (SD):<br>35.6 (12.6)/<br>-Chronic<br>-1 y	-Race<br>-Education<br>-Marital status<br>-Payer group<br>-Pre-injury employment	Female persons of all age groups more likely to work fewer hours after injury.<br><br>Female persons of all age groups, except the oldest (55-64 y), more likely than male to stop working after injury.<br><br>Female persons of all age groups, except the oldest (55-64 y), less likely than male to keep the same work hours after injury. In the oldest age group, male less likely than female to keep the same hours.<br><br>Female's likelihood of decreasing hours or stopping work more pronounced in married females.<br><br>Divorced male and female persons have a comparable likelihood of decreasing hours, while continuing working, but divorced females more likely to stop working than divorced males.

Disability[87]<br><br>Potentially sex	Mean (SD):<br>42 (20)/<br>-Acute<br>-Rehab discharge<br>-Mean (SD) interval between injury and rehab admission: 14 (7) d	-Marital status<br>-Race<br>-Insurance<br>-SES<br>-Education<br>-Employment status	Female persons have worse functional outcomes, as measured by the FIM at discharge from rehab and 1 y post injury.<br><br>Sex not associated with functional outcomes, as measured by the DRS at discharge from rehab and 1 y post injury or the GOSE 1 y post injury.
Disability[88]<br><br>Potentially sex	Mean (SD) (at injury):<br>30.9 (11.2)/<br>-Chronic<br>-1, 2, 5 y	-Relationship status<br>-Education<br>-Pre-injury employment status<br>-Occupation	Sex not associated with disability at 1, 2, and 5 y post injury, as measured by the GOSE.
Favorable outcome by GOS[91]<br><br>Sex/gender<br><br>OR (CI)	Mean (SD):<br><u>Male persons</u><br>50.4 (21.5)<br><u>Female persons</u><br>61.4 (22.6)/<br>-Chronic<br>-6 m	None	Sex/gender not associated with ICU survival, hospital survival, or favourable outcome (GOS).
Life satisfaction[98]<br><br>Potentially sex	Mean (SD):<br>35.87 (16.14)/<br>-Chronic<br>-1, 2, 5, 10 y	- Race<br>-Relationship status<br>-Employment status<br>-Annual earnings<br>-Education	Sex not associated with life satisfaction post injury or over time.
Social participation[58]<br><br>Potentially sex	Frequency of age groups:<br>-16-24 (27%)<br>-25-34 (18.6%)<br>-35-44 (18.8%)<br>-45-54 (12.3%)<br>-55-64 (7.1%)<br>-65+ (16.2%)/<br>-Chronic<br>-1 y	-Relationship status<br>-Ethnicity<br>-Governmental funding<br>-Employment at injury	Female persons have greater overall disability in social participation and in the domains of cognitive independence, mobility, and occupation.<br><br>Sex not associated with physical independence, social integration, and economic self-sufficiency.
Cognitive functioning[56]	Mean (SD):<br>34.37 (13.80)/	-Education	When considering weighted GRS, female persons display poorer cognitive functioning.

Sex/gender	-Chronic<br>-6, 12 m		Sex/gender not associated with cognitive functioning in the unweighted and semi-weighted GRS models.
Disability[75]<br><br>Sex/gender	Mean (SD):<br>Male persons<br>43.0 (18.6)<br>Female persons<br>53.7 (22.8)/<br>-Chronic<br>-6 m	None	Sex/gender not associated with poor outcome likelihood, but<br>when stratified by age, Female persons younger than 60 have poorer outcomes than Male persons, as measured by GOS.<br><br>Sex/gender not associated with outcome in those 60 and older.
Disability[29]<br><br>Sex/gender	Mean (SD):<br>Male persons<br>33.8 (16.3)<br>Female persons<br>43.3 (23.1)/<br>-Chronic<br>-6 m	None	Female persons less likely to have a good outcome, as measured by the GOSE.
Disability[109]<br><br>Life satisfaction<br><br>Potentially sex	Mean (range, SD):<br>Whole sample<br>26.85 (16-50, 9.11)<br>Male persons<br>27.06 (16-50, 8.97)<br>Female persons<br>26.30 (16-50, 9.49)/<br>-Chronic<br>-1 y	-Race<br>-Family income<br>-Education<br>-Martial status	Sex not associated with life satisfaction or motor functioning autonomy.<br><br>Female persons have better outcomes for cognitively medicated activities.
Cognitive functioning[89]<br><br>Sex/gender	Mean (SD):<br>65.02 (6.34)/<br>-Acute<br>-Chronic<br>Mean (SD) time since injury:<br>20.90 (34.92) m	-Education<br>-Residence (urbanization)	Male persons associated with better clinical dementia rating scores and performance in some cognitive functioning domains: confrontational and generative naming.<br><br>Sex/gender not associated with functional outcome, as measured by the Clinical Dementia Rating.

Disability[90]<br><br>Sex/gender	Mean (SD):<br><u>Male persons</u><br>36.2 (17.4)<br><u>Female persons</u><br>39.9 (20.7)/<br>-Chronic<br>-6 m	None	Female persons more likely to experience a bad outcome, as measured by GOSE.<br><br>Sex/gender by age effect:<br><br>Sex/gender not associated with outcome for people aged less than 30, as measured by GOSE.<br><br>Female persons 30 and older more likely to experience a bad outcome than Male persons.<br><br>Sex/gender not associated with functional status, as measured by the FSE, in any of the models, except for one involving people 30 or older. Here, female persons displayed poorer outcomes.
Disability[92]<br>Sex/gender	Mean (SD):<br>40.6 (19.9)<br>-Chronic<br>-1 y	-Race<br>-Employment status at injury<br>-Funder	Female persons have worse outcomes with respect to physical independence, community integration, and health perception.
Disability[93]<br><br>Sex/gender	Mean (range, SD):<br><u>At injury</u><br>39.8 (15-84, 14.9)<br><u>At assessment</u><br>49.1 (17-92, 15.1) /<br>-Chronic<br>-Mean (range, SD) time since injury: 9.3 (1-55, 8.4) y	-Education	Sex/gender not associated with outcome, as measured by GOSE.
Disability[94]<br><br>Sex/gender	Mean (SD):<br><u>Female persons</u><br>55.1 (18.9)<br><u>Male persons</u><br>46.4 (18.6)/<br>-Acute<br>-12 w	-Pre-injury living situation<br>-Pre-injury employment	Sex/gender not associated with outcome, as measured by GOS, living situation, or employment

Disability[95]<br><br>Quality of life[95]<br><br>Sex/gender	Mean (SD):<br>At injury<br>35.6 (13.1)<br>At assessment<br>38.8 (13.2)/<br>-Chronic<br>-1 – 6 y	-Change vs. no change in social participation post injury<br>-Suitability of family support<br>-Psychosocial reintegration	Female persons have worse physical function outcomes as related to quality of life.<br><br>Sex/gender not associated with social quality of life components.
Return to work[96]<br><br>Sex/gender	Mean (range, SD):<br>Whole sample<br>39.2 (15-78, 17.8)/<br>-Chronic<br>-Mean (SD) time since injury:<br>Minor/mild TBI<br>6 m (2 w)<br>Moderate-severe TBI<br>12 m (4 w)	None	Sex/gender associated with RTW only in minor TBI.
Cognitive functioning[103]<br><br>Sex/gender	Mean (SD) (baseline):<br>20s: 22.61 (1.51)<br>40s: 42.62 (1.49)<br>60s cohort: 62.51 (1.51)/<br>-Chronic<br>-NR	None	Male persons in their 20s performed worse than female in the same age group in verbal ability.<br><br>Sex/gender not associated with verbal ability in 40s and 60s.
Positive head CT scan[59]<br><br>Sex/gender	Median (IQR):<br>39 (24-55)<br>-Acute<br>-NR (during hospital stay)	-Race<br>-Insurance coverage	Sex/gender not associated with positive CT scan findings.
Neck injury comorbidity with concussion[71]<br><br>Sex/gender	Frequency of neck injury in concussion across age groups:<br>Male persons<br>58.13%<br>Female persons<br>41.87%/<br>-Acute<br>-NR (at hospital entry)	-Income<br>-Rurality	Female persons of reproductive ages more likely than Male persons to sustain comorbid neck injury with concussion.

Intrinsic functional connectivity of resting-state networks[69]<br><br>Potentially sex	Mean (SD) (baseline):<br><u>Male persons</u><br>35.4 (9.7)<br><u>Female persons</u><br>35.6 (9.4)/<br>-Acute<br>-NR (at hospital entry)	-Education	Male persons had greater intrinsic functional connectivity in motor/<br>executive functions, ventral stream, and cerebellum networks post- mTBI<br><br>Female persons had greater connectivity in the visual network post mTBI.
Systolic blood pressure[80]<br><br>Sex/gender	Mean (SD):<br><u>Male persons</u><br>44.8 (21.6)<br><u>Female persons</u><br>40.7 (17.3)/<br>-Acute<br>-NR (during hospital stay)	None	Factors related to changes in systolic blood pressure increase in male persons.
Cerebral oxygenation after red blood cell transfusion[73]<br><br>Sex/gender	Mean (SD):<br><u>Whole sample</u><br>35.4 (15.9)<br><u>Male persons</u><br>32.8 (13.9)<br><u>Female persons</u><br>45.5 (19.4)/<br>-Acute<br>-NR (during hospital stay)	None	Female persons exhibit greater cerebral oxygenation in response to red blood cell transfusion.
CSF markers of damage[78]<br><br>Sex/gender	Mean (SD):<br>31.5 (1.7)/<br>-Acute<br>-1-3 d	None	Male persons exhibit greater oxidative damage and an<br>increase in oxidative damage markers over three days, while female persons exhibit a decrease.
CSF markers of damage[79]<br><br>Sex/gender	Mean (SD):<br><u>Whole sample</u><br>31.49 (1.80)<br><u>Male persons</u><br>31.42 (2.04)<br><u>Female persons</u>	None	Sex/gender interacts with DAT (DA transporter) genotype in Female persons:<br>Those with 10/10 genotype have higher DA (potential marker of oxidative injury) levels than 9/10 and 9/9 genotypes (no interaction in Male persons).

	31.73 (3.99)/<br>-Acute<br>-1-5 d		Female persons have higher levels of DOPAC (product of DA), but levels do not differ from normative values.<br><br>Sex/gender not associated with HVA (DA metabolite).
Neurosurgical intervention[59]<br>Sex/gender	Median (IQR):<br>39 (24-55)<br>-Acute<br>-NR (during hospital stay)	-Race<br>-Insurance coverage	Male persons more likely than female to undergo neurosurgical intervention.
Discharge destination[72]<br>Sex/gender	Mean age (SD)<br>Pre-Medicare's inpatient rehab facility prospective payment system: 35 (22.5)<br>Post: 37.0 (23.5)/<br>Acute	-Race<br>-Insurance<br>- Prospective payment system status	Male persons less likely to be discharged to inpatient rehabilitation.
Discharge destination[105]<br>Sex/gender	Median age group:<br>Male persons<br>70-74<br>Female persons<br>75-79/<br>-Acute (at hospital discharge)<br>-Mean (SD) stay length<br>Male persons<br>17.0 (25.2)<br>Female persons<br>17.0 (26.5)	None	Female persons more likely to be discharged to a care facility rather than their home.
Care[92]<br>Sex/gender	Mean (SD):<br>40.6 (19.9)<br>-Chronic<br>-1 y	-Minority status<br>-Pre-injury working status<br>-Funding	Female persons more likely to receive rehabilitation and use services in the community.<br>Sex/gender not associated with admission to LTC.
Mortality[80]<br>Sex/gender	Mean (SD):<br>Male persons<br>44.8 (21.6)<br>Female persons<br>40.7 (17.3)/<br>-Acute	None	Factors associated with mortality are the same for both sexes with TBI, with the exception of SBP ≥ 160 mmHg for males.

	-NR (during hospital stay)		
Mortality[100]<br><br>Sex/gender	Mean (SD):<br>44.6 (21.8)<br>Median (range):<br>42 (14-89)<br>-Acute<br>-NR (during hospital stay)	None	Female persons associated with reduced mortality and fewer complications.
Mortality[101]<br><br>Sex/gender	Frequency of age groups:<br>Whole sample<br>-15-49 (73%)<br>-50+ (27%)<br>Male persons<br>-15-49 (77%)<br>-50+ (23%)<br>Female persons<br>-15-49 (59%)<br>-50+ (41%)/<br>-Acute<br>-NR (during hospital stay)	None	Sex/gender not associated with mortality in all but two age groups: 50-59 and 70+.<br>In these two age groups, being a female was associated with reduced mortality.
ICU survival, hospital survival[91]<br>Sex/gender	Mean (SD):<br>Male persons<br>50.4 (21.5)<br>Female persons<br>61.4 (22.6)/<br>-Acute<br>-In hospital	None	Sex/gender not associated with ICU or hospital survival.
Withdrawal of life sustaining therapies[76]<br>Sex/gender	Mean (SD):<br>50.7 (21.7)/<br>-Acute<br>-Median (IQR):<br>3 (1 – 7) d	None	Sex/gender not associated with withdrawal of life-sustaining therapies.

Mortality[75]<br>Sex/gender	Mean (SD):<br><u>Male persons</u><br>43.0 (18.6)<br><u>Female persons</u><br>53.7 (22.8)/<br>-Chronic<br>-6 m	None	Sex/gender not associated with mortality.
Mortality[55]<br>Sex/gender	Mean (SD):<br><u>Whole sample</u><br>45.3 (22.1)<br><u>Male persons</u><br>43.0 (20.7)<br><u>Female persons</u><br>50.8 (26.6) /<br>-Acute (during hospital stay)<br>-1-75 days	None	Being a female associated with increased mortality.
Mortality[29]<br>Sex/gender	Mean (SD):<br><u>Male persons</u><br>33.8 (16.3)<br><u>Female persons</u><br>43.3 (23.1)/<br>-Chronic<br>-6 m	None	Sex/gender not associated with mortality, except for those females > 60 (increased mortality).
Mortality[99]<br>Sex/gender	Mean (SD):<br><u>Male persons</u><br>76.8 (7.5)<br><u>Female persons</u><br>78.9 (7.7)<br>-Acute (at hospital discharge)<br>-Stay lengths in days	-Race	<u>Isolated TBI (no injury to other body regions):</u><br>Sex/gender not associated with mortality.<br><br><u>All TBIs:</u><br>Being a female associated with reduced mortality.
Mortality 12 years after TBI[108]<br><br>Sex/gender	Mean (SD):<br>42.8 (25.3)/<br><br>Up to 12 years from hospital discharge	-Race<br>-Insurance status	Female persons have a lower risk of death with TBI.
Mortality[102]	Mean (SD): 69 (1)/	None	Sex/gender not associated with mortality.

Sex/gender	-Acute (during hospital stay)<br>-Mean (SD) length of stay:<br>8 (0.4) d		
Perceived inadequacy[17]<br><br>Sex/gender	Range:<br>33-46/<br>-Chronic<br>-Residential facility	None	Male persons expressed more feelings of sex inadequacy post-injury and depended more heavily on traditional sex-specific activities before and after injury to define and support sex role.<br><br>Female persons relied more on cross-sex activities and were able to maintain more pre-injury activities post-injury.
Perceived dependence, self-identity, and esteem[84]<br><br>Gender<br><br>TBI severity frequencies:<br>-Mild (50%)<br>-Moderate-severe (50%)	Mean (SD)<br>Acute:<br>Male persons<br>48.6 (19.9)<br>Female persons 42.9 (22.7)<br>Chronic:<br>Male persons<br>50.8 (14)<br>Female persons<br>44.1 (16.8)/<br>-Acute and chronic (<6 months post-injury)<br>-Rehab hospital	-Ethnicity<br>-Education<br>-Level of femininity/ masculinity<br>-Marital status	Gendered pre-injury roles and responsibilities are disturbed post-injury in both genders. Female persons feel guilt and male persons feel emasculated.<br>New roles and responsibilities not formed yet and many are taken on by significant others. Both genders are dependent on others.

Abbreviations: AIS: Abbreviated Injury Scale; ASDS: Acute Stress D/O Scale; ASDS-DISS: Acute Stres D/O Scale-Dissociative Score; AUD: Alcohol Use D/O; BDI-II: Beck Depression Inventory II; CI: confidence interval; CPP: cerebral perfusion pressure; CT: computed tomography; DAT: DA transporter; DSM: Diagnostic and Statistical Manual of Male personstal D/Os; FIM: Functional Independence Measure; GCS: Glasgow Coma Scale; GOSE: Glasgow Outcome Scale Extended; GRS: gene risk score; ICU: intensive care unit; ISS: injury severity score; LOC: loss of consciousness; LOS: length of stay; MRI: magnetic resonance imaging; mTBI: mild traumatic brain injury; MVA: motor vehicle accident; NR: not reported; PPS: prospective payment system; PTA: post-traumatic amnesia; PTSD: Post-Traumatic Stress D/O; REM: rapid eye moveMale personst; RTW: return to work; SBP: systolic blood pressure; SCI: spinal cord injury; SD: standard deviation; SDMT: Symbol Digit Modalities Test; SES: socioeconomic status; SSRI: Selective serotonin reuptake inhibitor; SUB: Substance Use D/O; and TBI: traumatic brain injury.

Supplementary Figure 1: Effect sizes of factors/variance explained by factors investigated in relation to post-TBI outcomes in multivariate regression analyses.

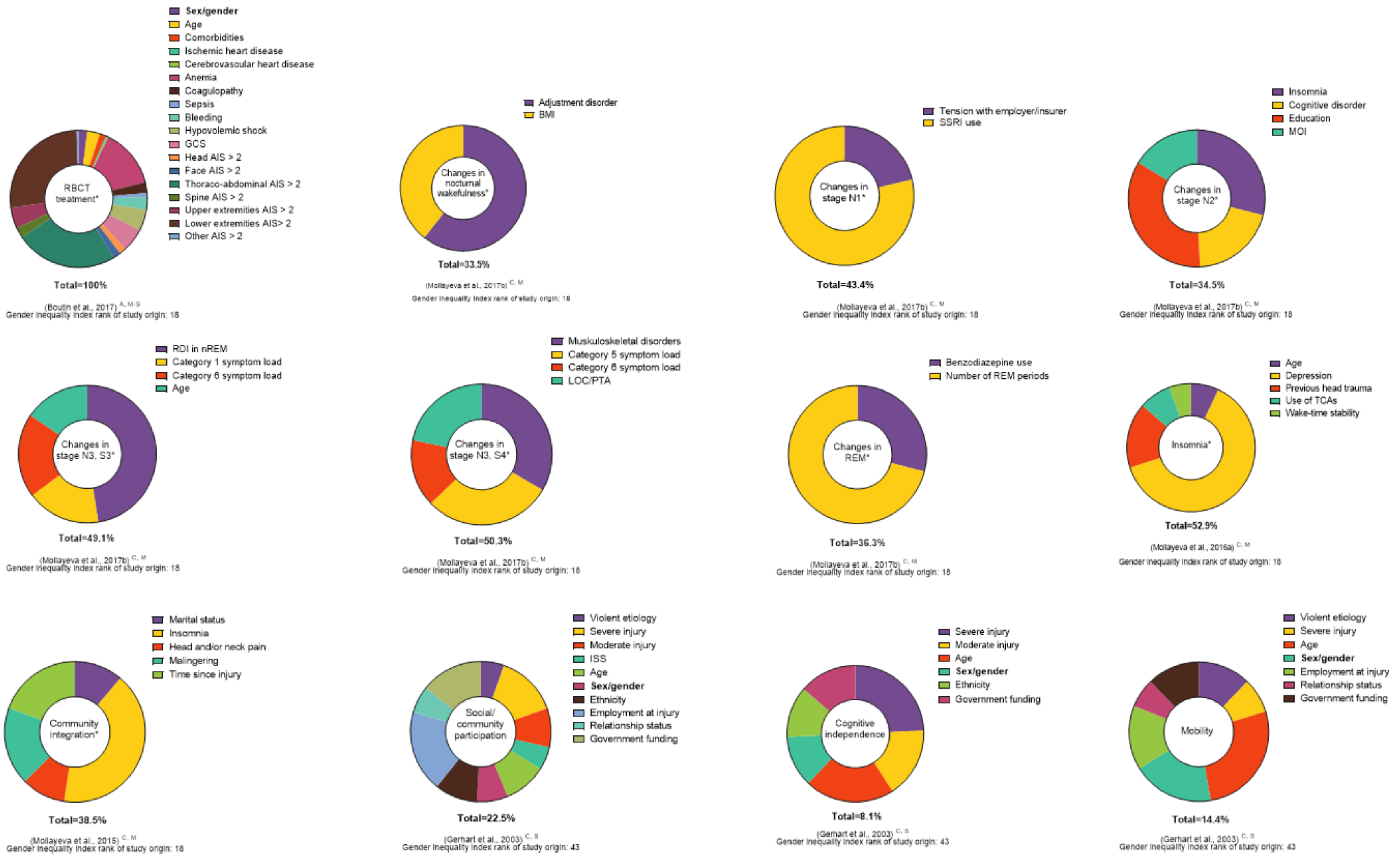

Sex/gender
Age
Comorbidities
Ischemic heart disease
Cerebrovascular heart disease
Anemia
Coagulopathy
Sepsis
Bleeding
Hypovolemic shock
GCS
Head AIS > 2
Face AIS > 2
Thoraco-abdominal AIS > 2
Spine AIS > 2
Upper extremities AIS > 2
Lower extremities AIS> 2
Other AIS > 2
RBCT treatment*
Total=100%
(Boutin et al., 2017) A, M-S
Gender inequality index rank of study origin: 18
Adjustment disorder
BMI
Changes in nocturnal wakefulness*
Total=33.5%
(Mollayeva et al., 2017b) C, M
Gender inequality index rank of study origin: 18
Tension with employer/insurer
SSRI use
Changes in stage N1*
Total=43.4%
(Mollayeva et al., 2017b) C, M
Gender inequality index rank of study origin: 18
Insomnia
Cognitive disorder
Education
MOI
Changes in stage N2*
Total=34.5%
(Mollayeva et al., 2017b) C, M
Gender inequality index rank of study origin: 18
RDI in nREM
Category 1 symptom load
Category 6 symptom load
Age
Changes in stage N3, S3*
Total=49.1%
(Mollayeva et al., 2017b) C, M
Gender inequality index rank of study origin: 18
Muskuloskeletal disorders
Category 5 symptom load
Category 6 symptom load
LOC/PTA
Changes in stage N3, S4*
Total=50.3%
(Mollayeva et al., 2017b) C, M
Gender inequality index rank of study origin: 18
Benzodiazepine use
Number of REM periods
Changes in REM*
Total=36.3%
(Mollayeva et al., 2017b) C, M
Gender inequality index rank of study origin: 18
Age
Depression
Previous head trauma
Use of TCAs
Wake-time stability
Insomnia*
Total=52.9%
(Mollayeva et al., 2016a) C, M
Gender inequality index rank of study origin: 18
Marital status
Insomnia
Head and/or neck pain
Malingering
Time since injury
Community integration*
Total=38.5%
(Mollayeva et al., 2015) C, M
Gender inequality index rank of study origin: 18
Violent etiology
Severe injury
Moderate injury
ISS
Age
Sex/gender
Ethnicity
Employment at injury
Relationship status
Government funding
Social/ community participation
Total=22.5%
(Gerhart et al., 2003) C, S
Gender inequality index rank of study origin: 43
Severe injury
Moderate injury
Age
Sex/gender
Ethnicity
Government funding
Cognitive independence
Total=8.1%
(Gerhart et al., 2003) C, S
Gender inequality index rank of study origin: 43
Violent etiology
Severe injury
Age
Sex/gender
Employment at injury
Relationship status
Government funding
Mobility
Total=14.4%
(Gerhart et al., 2003) C, S
Gender inequality index rank of study origin: 43

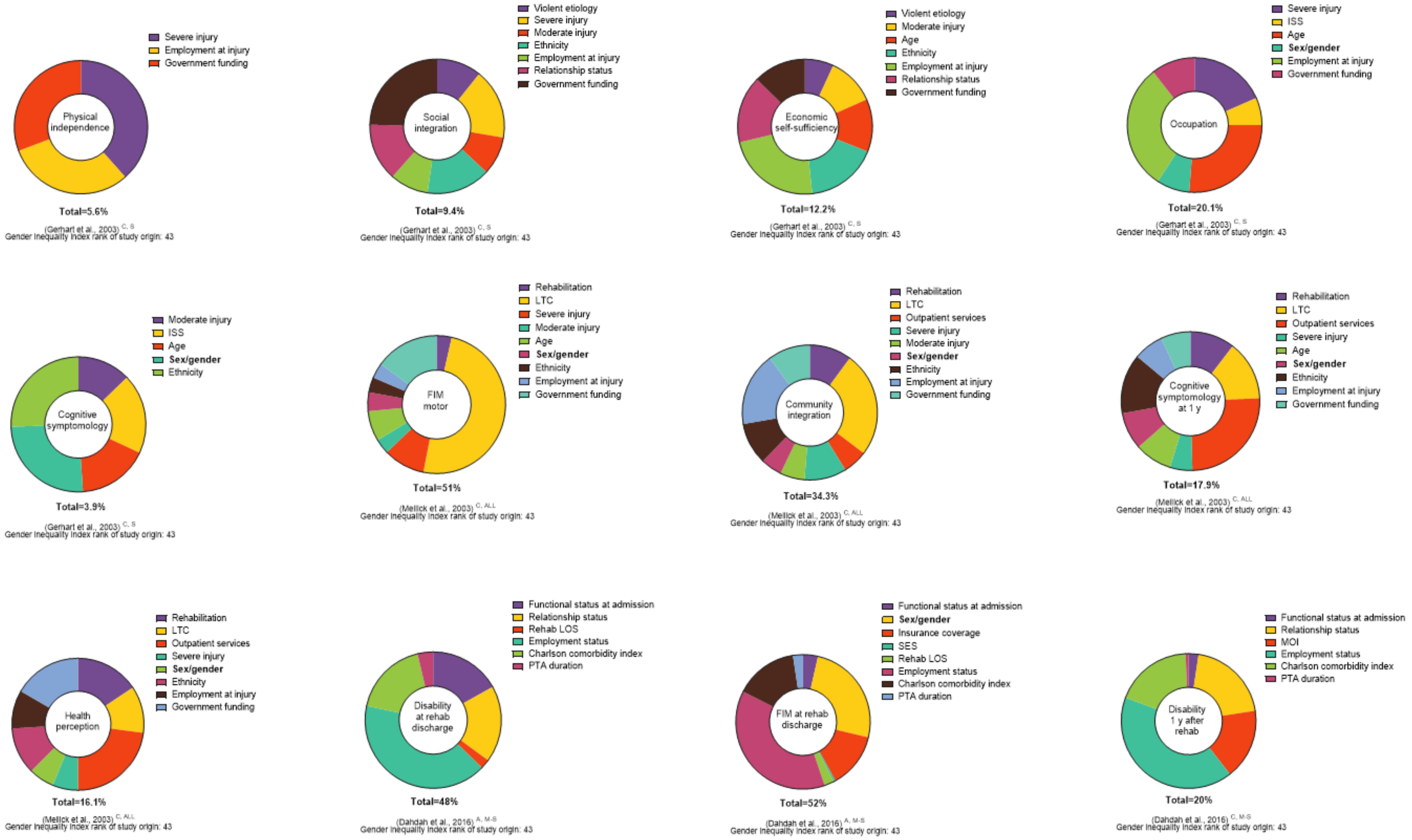
Physical independence
Severe injury
Employment at injury
Government funding
Total=5.6%
(Gerhart et al., 2003) C, S
Gender Inequality Index rank of study origin: 43
Social integration
Violent etiology
Severe injury
Moderate injury
Ethnicity
Employment at injury
Relationship status
Government funding
Total=9.4%
(Gerhart et al., 2003) C, S
Gender Inequality Index rank of study origin: 43
Economic self-sufficiency
Violent etiology
Moderate injury
Age
Ethnicity
Employment at injury
Relationship status
Government funding
Total=12.2%
(Gerhart et al., 2003) C, S
Gender Inequality Index rank of study origin: 43
Occupation
Severe injury
ISS
Age
Sex/gender
Employment at injury
Government funding
Total=20.1%
(Gerhart et al., 2003) C, S
Gender Inequality Index rank of study origin: 43
Cognitive symptomology
Moderate injury
ISS
Age
Sex/gender
Ethnicity
Total=3.9%
(Gerhart et al., 2003) C, S
Gender Inequality Index rank of study origin: 43
FIM motor
Rehabilitation
LTC
Severe injury
Moderate injury
Age
Sex/gender
Ethnicity
Employment at injury
Government funding
Total=51%
(Mellick et al., 2003) C, ALL
Gender Inequality Index rank of study origin: 43
Community integration
Rehabilitation
LTC
Outpatient services
Severe injury
Moderate injury
Sex/gender
Ethnicity
Employment at injury
Government funding
Total=34.3%
(Mellick et al., 2003) C, ALL
Gender Inequality Index rank of study origin: 43
Cognitive symptomology at 1 y
Rehabilitation
LTC
Outpatient services
Severe injury
Age
Sex/gender
Ethnicity
Employment at injury
Government funding
Total=17.9%
(Mellick et al., 2003) C, ALL
Gender Inequality Index rank of study origin: 43
Health perception
Rehabilitation
LTC
Outpatient services
Severe injury
Sex/gender
Ethnicity
Employment at injury
Government funding
Total=16.1%
(Mellick et al., 2003) C, ALL
Gender Inequality Index rank of study origin: 43
Disability at rehab discharge
Functional status at admission
Relationship status
Rehab LOS
Employment status
Charlson comorbidity index
PTA duration
Total=48%
(Dahdah et al., 2016) A, M-S
Gender Inequality Index rank of study origin: 43
FIM at rehab discharge
Functional status at admission
Sex/gender
Insurance coverage
SES
Rehab LOS
Employment status
Charlson comorbidity index
PTA duration
Total=52%
(Dahdah et al., 2016) A, M-S
Gender Inequality Index rank of study origin: 43
Disability 1 y after rehab
Functional status at admission
Relationship status
MOI
Employment status
Charlson comorbidity index
PTA duration
Total=20%
(Dahdah et al., 2016) C, M-S
Gender Inequality Index rank of study origin: 43

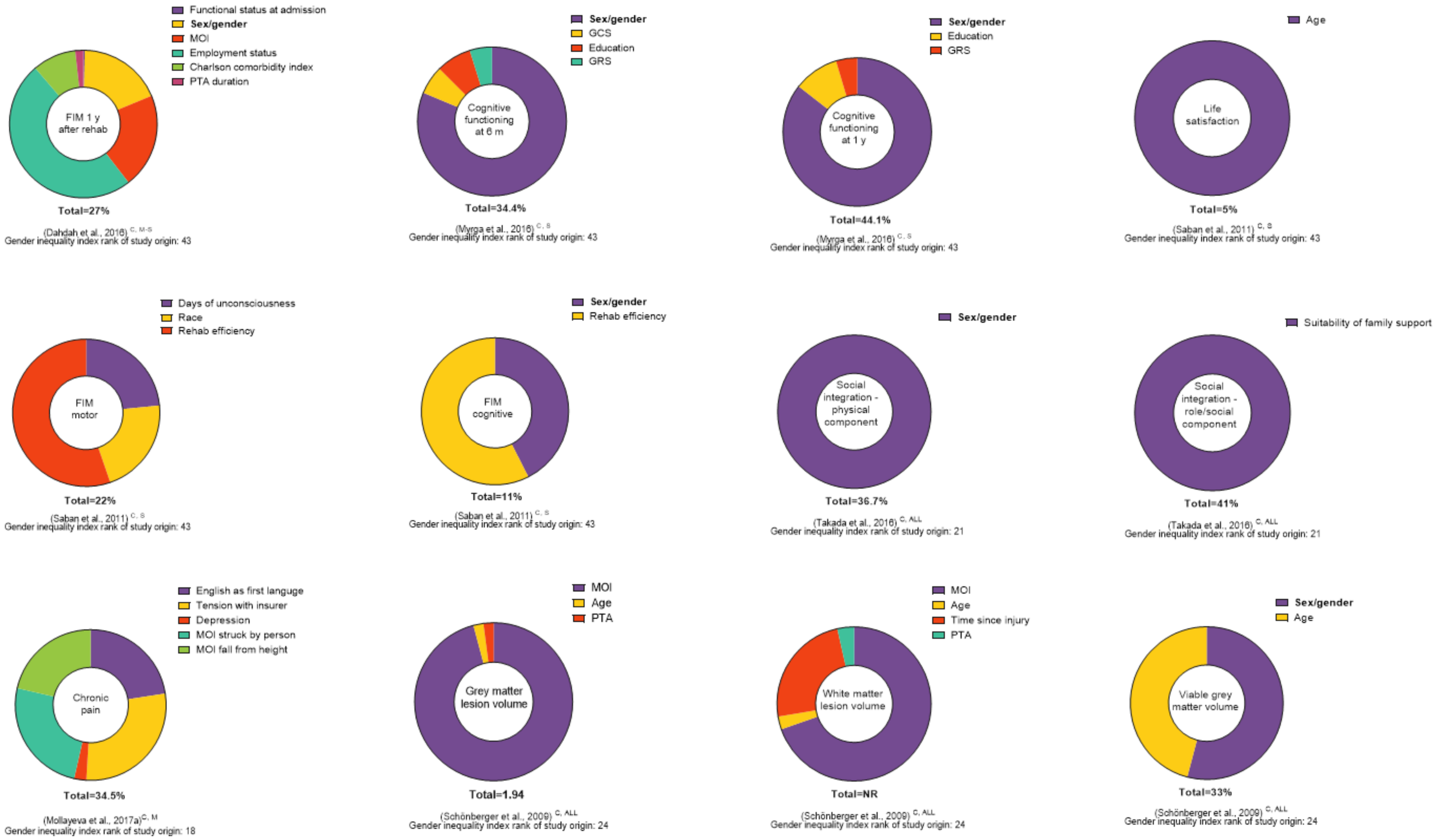
Functional status at admission
Sex/gender
MOI
Employment status
Charlson comorbidity index
PTA duration
FIM 1 y after rehab
Total=27%
(Dahdah et al., 2016) C, M-S
Gender inequality index rank of study origin: 43
Sex/gender
GCS
Education
GRS
Cognitive functioning at 6 m
Total=34.4%
(Myrga et al., 2016) C, S
Gender inequality index rank of study origin: 43
Sex/gender
Education
GRS
Cognitive functioning at 1 y
Total=44.1%
(Myrga et al., 2016) C, S
Gender inequality index rank of study origin: 43
Age
Life satisfaction
Total=5%
(Saban et al., 2011) C, S
Gender inequality index rank of study origin: 43
Days of unconsciousness
Race
Rehab efficiency
FIM motor
Total=22%
(Saban et al., 2011) C, S
Gender inequality index rank of study origin: 43
Sex/gender
Rehab efficiency
FIM cognitive
Total=11%
(Saban et al., 2011) C, S
Gender inequality index rank of study origin: 43
Sex/gender
Social integration - physical component
Total=36.7%
(Takada et al., 2016) C, ALL
Gender inequality index rank of study origin: 21
Suitability of family support
Social integration - role/social component
Total=41%
(Takada et al., 2016) C, ALL
Gender inequality index rank of study origin: 21
English as first languge
Tension with insurer
Depression
MOI struck by person
MOI fall from height
Chronic pain
Total=34.5%
(Mollayeva et al., 2017a) C, M
Gender inequality index rank of study origin: 18
MOI
Age
PTA
Grey matter lesion volume
Total=1.94
(Schönberger et al., 2009) C, ALL
Gender inequality index rank of study origin: 24
MOI
Age
Time since injury
PTA
White matter lesion volume
Total=NR
(Schönberger et al., 2009) C, ALL
Gender inequality index rank of study origin: 24
Sex/gender
Age
Viable grey matter volume
Total=33%
(Schönberger et al., 2009) C, ALL
Gender inequality index rank of study origin: 24

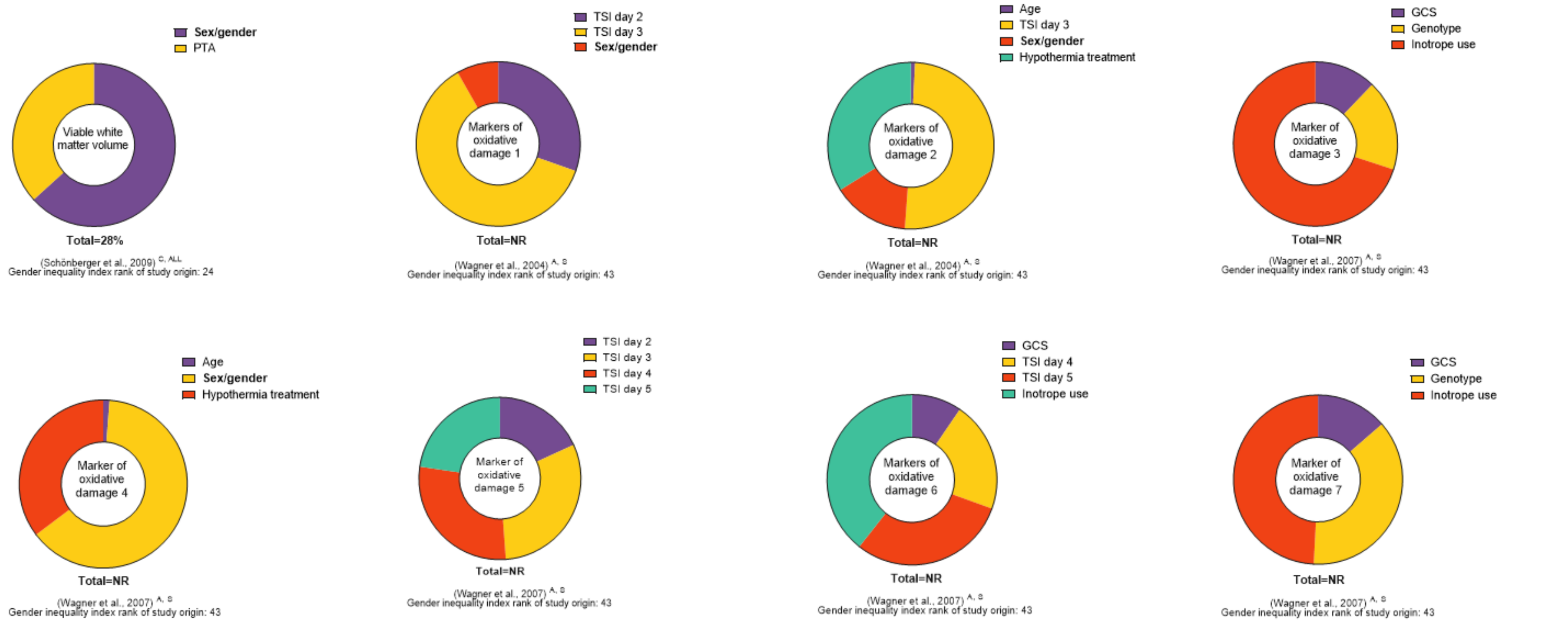

Notes: For outcomes marked with *, proportions correspond to partial R2. For all other outcomes, the proportions correspond to β coefficients. Only statistically significant factors included as explaining variance given that cut-off p-values not consistently reported. Superscripts indicate post injury phase (acute (A), chronic (C), not reported (NR)), and injury severity (mild (M), moderate-severe (M-S), severe (S), all severities (ALL)).

Oxidative damage markers: 1: Isoprostane/lactate/pyruvate; 2: isoprostane/glutamate; 3: dopamine (DA); 4: 3,4-dihydroxyphenylacetic acid (DOPAC, DA product); 5: homovanillic acid (HVA, DA metabolite); 6: DOPAC/DA (indicator of metabolic turnover); 7: HVA/DA (indicator of metabolic turnover).
